# The effect of shotgun barrel extension chokes on pellet dispersion and shooting distance determinations

**DOI:** 10.1007/s00414-026-03858-2

**Published:** 2026-06-08

**Authors:** Can Yücel Şahan, Nergis Cantürk, Ercan Seyhan

**Affiliations:** 1 Ministry of Environment, Urbanization and Climate Change, Ankara, Türkiye; 2 Faculty of Medicine, Department of Forensic Medicine, Ankara University, Ankara, Türkiye; 3 Post Graduate School of Forensic Sciences, Hasan Kalyoncu University, Gaziantep, Türkiye

**Keywords:** Barrel Length, Extension Choke, Pellet Dispersion, Shotgun, Shooting Distance, Statistics

## Abstract

**Background:**

Determining the shooting distance is one of the most crucial factors in firearm injuries and fatalities. When determining distance, it is important to carefully consider the barrel length, cartridge and fuse structures, extension, and environmental conditions. This study aims to measure the distance and ascertain the effects of extensions on pellet dispersal.

**Results:**

It was determined that there were noticeable differences in the pellet distributions between rounds fired at the same distance without extensions and those fired with 5-10-15 cm extensions. The diameter of the pellet dispersion decreased statistically as the choke length increased at distances greater than 100 cm (*p* < 0,05). It was also seen that the diameter of the pellet entry hole shrank in rounds fired between 200 and 400 cm due to an increase in extension length. Statistics showed that the differences were significant (*p* < 0.05). The largest satellite entrance hole distance decreased with extension length at 200 and 300 cm. There were statistically significant differences in satellite entrance hole distance (*p* < 0.05).

**Conclusions:**

The study’s findings showed that the shotgun extensions directly impact pellet dispersal. It was acknowledged that the shot distance should not be calculated solely on the basis of target features without carefully analyzing the weapon and cartridge used in the incident.

**Supplementary Information:**

The online version contains supplementary material available at 10.1007/s00414-026-03858-2.

## Background

In firearm injuries and related deaths, the distance of the shot is as important as the person who fired the shot and the weapon with which the shot was fired. For forensic scientists, the determination of the shooting distance in death cases is an important criterion in determining whether the incident is an accident, suicide, or homicide.

When a shotgun is discharged, the pellets emerge from the barrel’s muzzle as a compact mass that moves quickly. The entrance wound’s diameter grows smaller when the shotgun’s muzzle is positioned farther away from the body until the pellets start to separate from the main mass. Shotguns are regarded as the most lethal and destructive small weapons when fired at close range. The number of pellets that enter the body, the organs they strike, and the degree of tissue damage all affect how deadly a shotgun wound is [1].

Numerous variables affect the pellet dispersion on a target surface and how it relates to distance. The shotgun type, barrel length, choke length, shotshell pressure, and environmental circumstances are a few of these. The patterns for a given shotgun charge and range vary with choke level [2].

To determine the firing distance, some characteristics of the weapon must be known. Regardless of the length of the barrel in rifling weapons, the distance at which the gunshot residues (GSR) are found on the skin or clothing is considered to be close range. In shotguns, in the absence of a single bullet, if there are no powder combustion products on the target, it is possible to determine the distance, even if the shot was fired from a long distance, when the pellet distribution is considered. Apart from pellet distribution on the target, shotgun barrel length, the number and weight of cartridges, the type of extension, and environmental factors should also be considered when determining the distance [3, 4].

Shotguns, which are easy to obtain legally or illegally, are frequently used in hunting activities as well as in homicides, suicides, and accidents. It has been reported that the number of homicides and suicides has increased in deaths due to shotgun injuries and that there is also an increase in accidental cases [5, 6].

The process of determining whether a shotgun injury is a homicide, suicide, or accident takes place from the crime scene investigation to the court stage. The most important evidence in determining the origin is obtained during the crime scene investigation [7, 8].

In homicidal shotgun wounds, the fatal shot may have been fired from any distance. The most important point that increases the likelihood of homicide is the presence of more than one gunshot wound on the body. In homicide cases, wounds are usually found on the back of the head and neck, chest and sides, back, abdomen, and in places inaccessible to the limbs. If the incident involves a long-distance shooting and there is no weapon at the scene, if the location of the wound on the body is not suitable for suicide, the distinction between accident and homicide is made through a forensic investigation. If there are multiple gunshot wounds, the origin is likely to be a homicide, since in accidental situations, there is likely to be a single wound [7, 8, 9, 10].

In the case of an accidental death, the first step is to assume that the death was not an accident. It may be difficult to determine whether the cause of death was accidental or homicide if the injury was to the neck, head, or chest. It is impossible to make a definite distinction from the body’s findings. In both cases, shots may have been fired near or far. In suicides, a specific location is preferred, whereas in accidents, there is no specific location. Accidental or homicidal wounds can appear on any random part of the body. However, if the wound is caused by an contact or near-contact shot, the likelihood of an accident is reduced [8, 10, 11].

In suicides with shotguns, the barrel is long and the person may pull the trigger with one hand while holding the end of the barrel with the other hand to stabilize the barrel. In these cases, gunpowder combustion products, smoke, or soot may be visible on the hand holding the barrel. However, in cases of homicide, the victim may hold the end of the gun pointed at him/her and try to change its direction. When shooting in this position, residual products of the shot can be seen in the hands of the person shot [7, 12, 13].

Multiple shots can sometimes be seen in suicide cases, albeit with low probability. In such cases, attention should be paid to the density of the shot residue on the hand. Shotguns that are called single or double-barreled shotguns, which cannot change the cartridge inside themselves, do not have gunshot residues on the shooting hand. If the barrel is held with both hands and the trigger is pulled with the foot, it is possible to find gunpowder residues on both hands. If the weapon is held against the shoulder and fired, gunshot residues may also be found on the face [8, 10, 12].

**In our study**, the effects of barrel lengthening extension chokes used in smoothbore shotguns on pellet distribution were investigated. 3 different lengths (5 cm, 10 cm, and 15 cm) extension chokes were used, and shots were fired at different distances. After each shot, the pellet distributions on the target were examined, and the effects of the extension chokes were analyzed, with the aim of determining the shooting distance. The shotguns with 71 cm barrels, 12 gauge, and 30 gr (No. 6) shotshells were fired at targets at 13 different ranges. Before firing the rounds with 5, 10, and 15 cm extensions, cartridges were fired without extensions. Each shot was fired vertically twice in succession toward the fabric ground target. Analysis of the acquired pellet distributions revealed that extensions had no impact on pellet dispersal up to a distance of 100 cm (*p* > 0.05). It was found that the width of the pellet distribution decreased with increasing choke length at distances greater than 100 cm.

This study aims to determine the effects of extension length on pellet distribution and shooting distance by using the same cartridge at different distances with shotguns fitted with different extension lengths.

## Methodology

### Materials

**Fig. 1:**
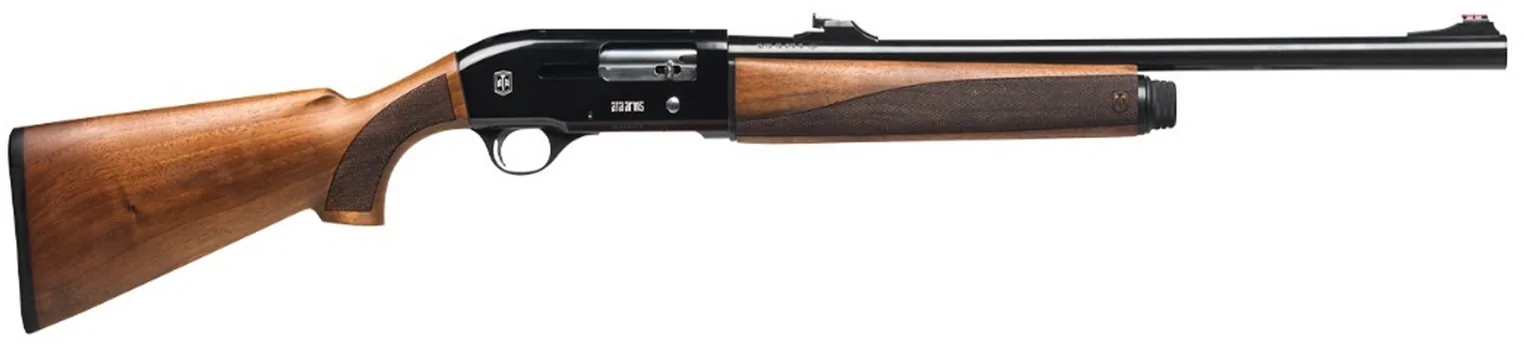
Ata arms CY semi-automatic shotgun

**Fig. 2:**
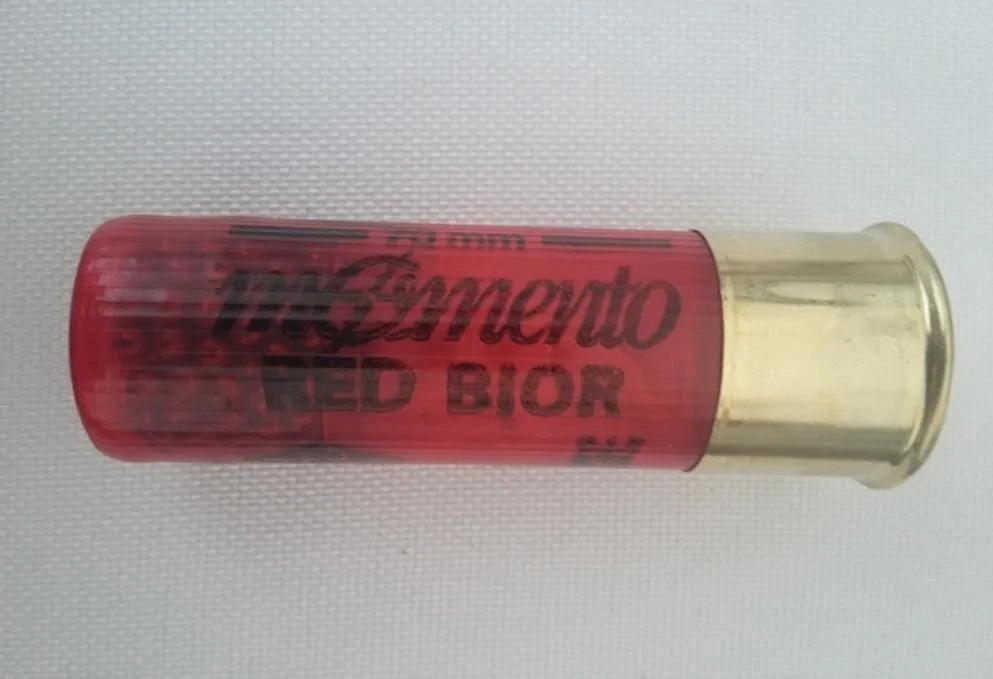
Momento No 6 30 gr cartridge

**Fig. 3:**
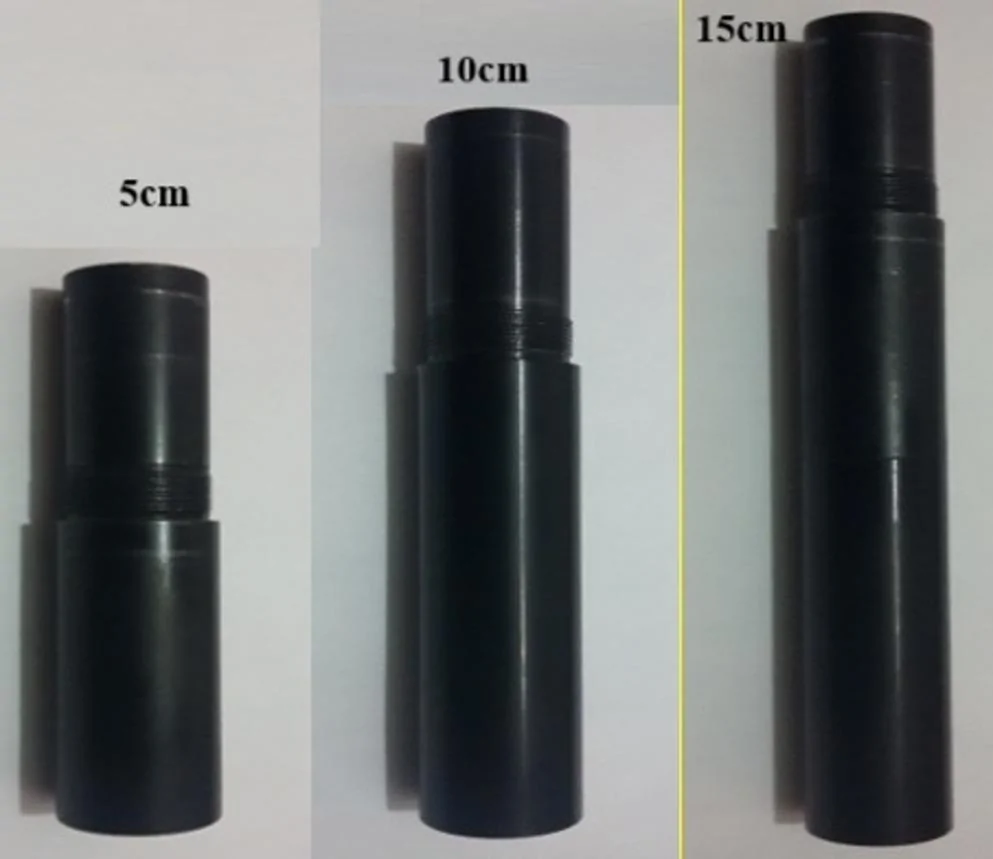
Ata Arms 5 cm,10 cm, and 15 cm extension chokes

**Fig. 4:**
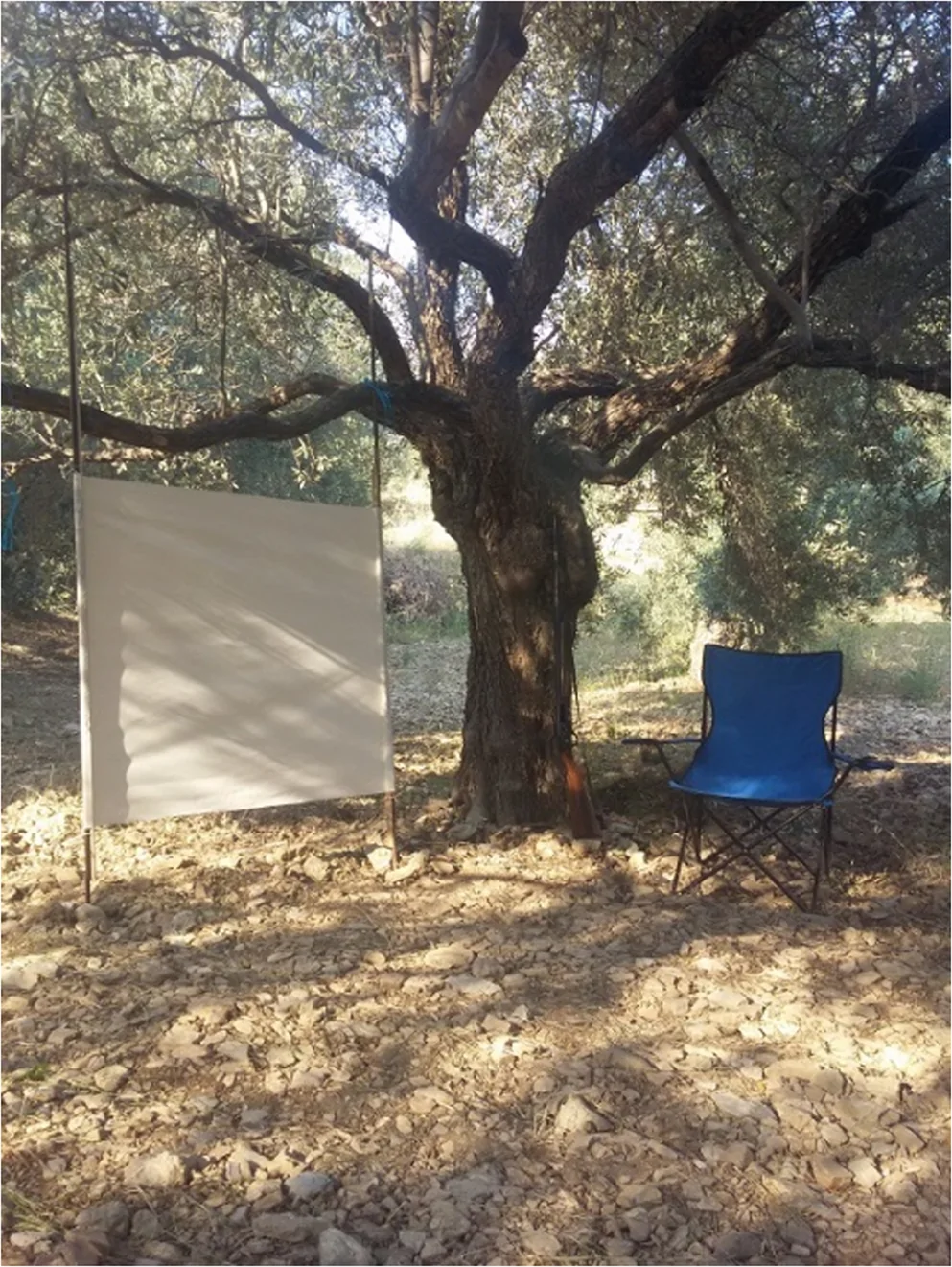
1,5 × 1,5 m nettle cloth

**Fig. 5:**
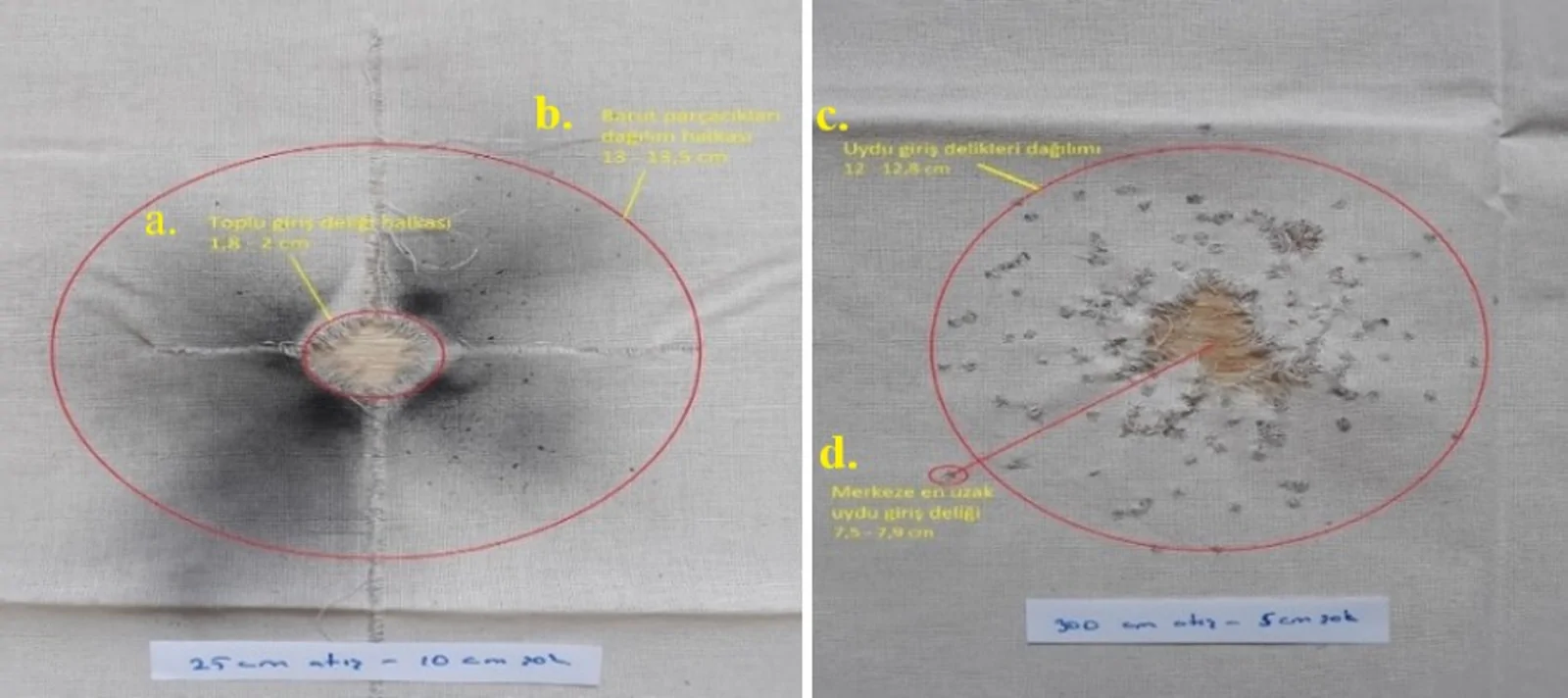
Examples of: (**a**) mass entry hole (**b**) particle distribution rings (**c**) distribution of satellite entry holes (**d**) satellite entry holes furthest from the center

In our study;

- Ata Arms brand 12 caliber semi-automatic shotgun (barrel length 71 cm) (Fig. 1) and No 6 cartridges (2.75 mm diameter, 12 calibers, 30 gram, 70 mm long) (Fig. 2),

(b) Ata Arms brand 5, 10, and 15 cm barrel extension chokes (Fig. 3).

(c) 1,5 × 1,5 m nettle cloth was used as a target (Fig. 4).

(d) The photographs used in the study were taken with a Samsung Galaxy J7 Pro mobile phone.
(e) Examples of mass entry holes *(from a 25 cm distance with a 10 cm extension choke AND a 300 cm distance with a 5 cm extension choke)*, powder particle distribution rings, distribution of satellite entry holes, and satellite entry holes furthest from the center are shown in Fig. 5.

(f) On May 2–6, 2018, the shootings took place in the Eşme Neighborhood (Datça District of Mula Province) under the weather that featured an average air temperature of 27 °C and an average wind speed of 10 km/h. The official authorities issued the necessary licenses for the firings.

### Method

In our study, shots were fired at cloth targets at 13 distances (contact, 5, 25, 50, 75, 100, 200, 300, 400, 500, 700, 800, and 1000 cm) without extension, and at 5, 10, and 15 cm extensions. Each shot was repeated 3 times, for a total of 156. The shots made are shown in Table 1.

**Table 1:** Distribution of shots fired

	Shot distance	Number of shots	Number of shots per distance	Extension length	Number of shots per extension type
1	Contact	3	3 × 13 = 39	No extension	3 × 4 = 12
2	5				3 × 4 = 12
3	25				3 × 4 = 12
4	50		3 × 13 = 39	5 cm	3 × 4 = 12
5	75				3 × 4 = 12
6	100				3 × 4 = 12
7	200		3 × 13 = 39	10 cm	3 × 4 = 12
8	300				3 × 4 = 12
9	400				3 × 4 = 12
10	500		3 × 13 = 39	15 cm	3 × 4 = 12
11	700				3 × 4 = 12
12	800				3 × 4 = 12
13	1000				
TOTAL 156					

Shots fired at each distance produced different pellet distributions, which were each measured separately. *The diameters of the soot dispersion*,* pellet distribution*,* satellite entrance hole distance from the bulk entrance hole*,* and powder particle diameter were measured and reported.* The measurements were taken by a single person twice each, and the average was then calculated to prevent deviations from the standardization. Using the measurements and computations, it was possible to evaluate, for fixed calibers and cartridges, the connection between the length of the extension and the distribution of pellets at a distance.

Changes in pellet distribution by choke extension length were assessed using a one-way ANOVA, and multiple comparisons were performed using the Tukey HSD test. As a supplementary file, the results are displayed in tables, and *p* < 0.05 was considered significant.

## Findings

12 bullets altogether, 3 without extension and 3 with 5–10–15 cm extensions, were fired at the target from each distance. Our results are below: (all related tables (Tables 2–109)) are presented as a Supplementary File)

###  Shots fired from contact (0 cm) distance

**Fig. 6:**
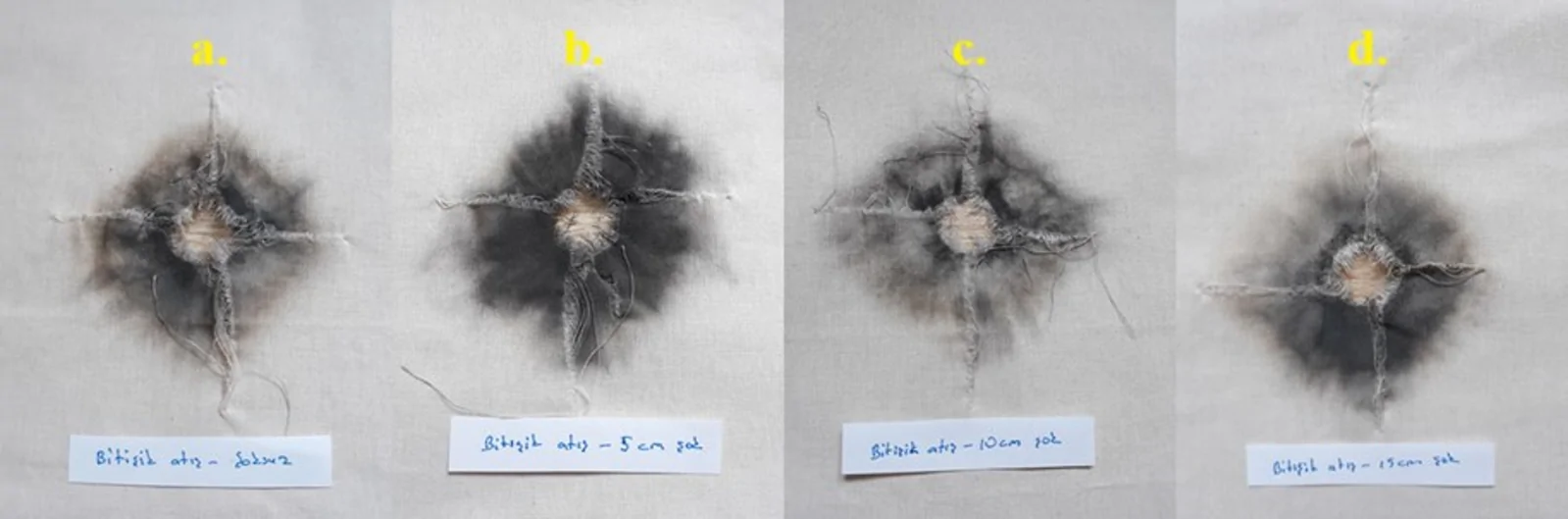
Contact distance shots: **a**.without extension **b**. with 5cm extension **c**. with 10cm extension **d**. With 15cm extension

Shots fired from a contact(0 cm) distance are shown in Fig. 6. Except for the mass entry hole of the pellets, all shots were seen to be homogeneous, and no satellite entry holes were found.

Entry hole diameters based on extension length at contact ranges in Table 2 compare the center entrance hole diameters at contact distances based on choke length. In all shots, it was noted that the center entrance holes’ average diameters (2.33–2.37 cm) were fairly close to one another. The comparisons led to the conclusion that the choke length had no discernible impact on the diameter of the center entrance hole at proximity.

- One-way analysis of variance was used to examine the sizes of the bullet entrance holes created by rounds fired from close ranges (Table 3). There was no statistically significant variation in the diameter of the entrance hole (*p* > 0,05). Multiple comparisons of pellet entry hole diameters across choke lengths were performed using the Tukey HSD test (Table 4). Since *p* > 0.05 in all of the comparisons, the differences between the pellet entry hole diameters were not statistically significant.
- The distribution of the muzzle soot on the target is compared (Table 5). The average soot distribution diameters (8.62–8.71 cm) were seen to be remarkably similar in all shots. One-way analysis of variance was used to compare the soot diameters obtained from rounds fired from contact ranges (Table 6), and the results showed that there was no statistically significant difference between the soot diameters (*p* > 0.05). The Tukey HSD test was used to compare soot sizes across various choke lengths (Table 7). The variations between the soot distribution diameters on the target surface were not statistically significant because *p* > 0.05 was used in all comparisons.

### Shots from 5 cm distance

**Fig. 7:**
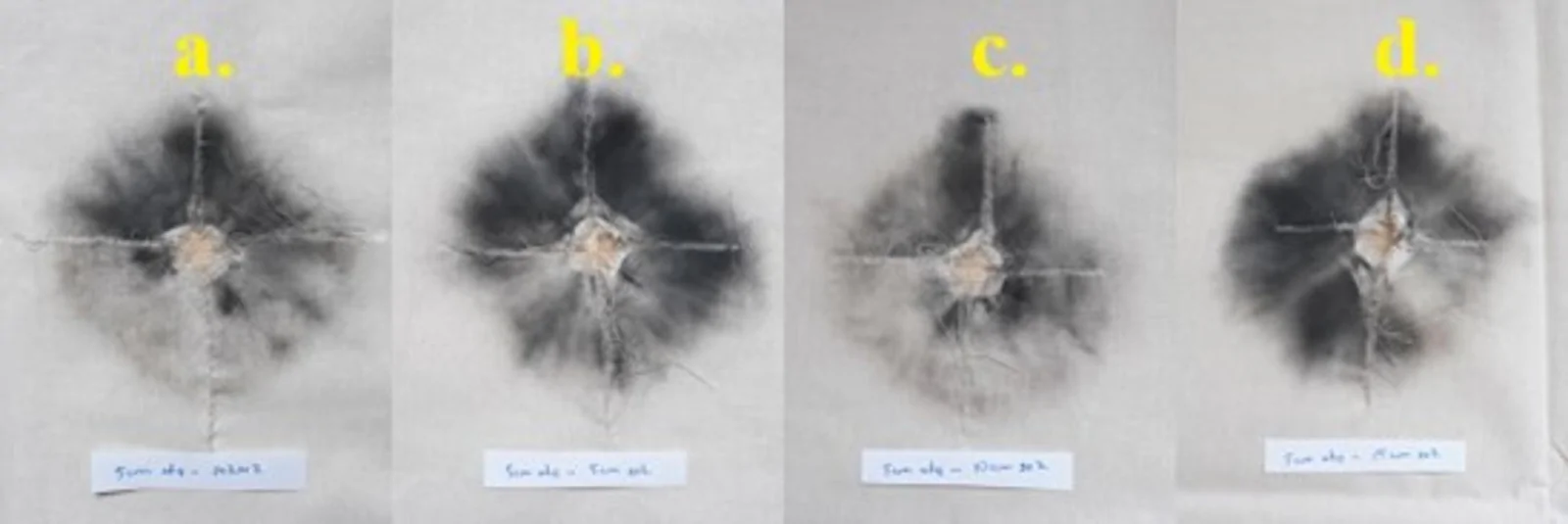
Shots from 5 cm distance: **a**.without extension **b**. with 5cm extension **c**. with 10cm extension **d**. With 15cm extension

Shots from 5 cm distance are shown in Fig. 7. All shots were observed to be homogeneous, and no satellite entry hole was detected except for the mass entry hole of the pellets.

The center entrance hole diameters for different extension lengths are compared in Table 8. It was determined that the average diameters of the center entrance holes (2.91–2.98 cm) were very close to each other in the shots made without extension and in the shots made with all extensions. As a result of these comparisons, it was determined that the extension length did not have a significant effect on the center entrance hole at a shooting distance of 5 cm. When compared with shots fired at the contact firing distance, it was found that the entrance holes of the pellets enlarged. The reason for this is not that the pellets reach the target by opening slightly due to the distance. The main reason is that the flame and gas exiting the barrel’s exit hole affect a larger area of the target. When fired from a distance of 5 cm, the flame and gas pressure coming out of the muzzle of the barrel have a strong effect on the cloth target. Due to this effect, a larger entrance hole is created on the target than that created by the pellets.

- One-way analysis of variance was used to determine the diameter of the entrance hole produced by rounds fired from a distance of 5 cm (Table 9). There was no statistically significant variation in the diameter of the entrance hole (*p* > 0.05). The Tukey HSD test was used to compare several mass entrance hole sizes based on extension length (Table 10). The differences between the mass entrance hole diameters were not statistically significant because *p* > 0.05 was used in all comparisons.
- The distribution of the muzzle soot on the target was compared (Table 11). It was found that the average diameter of the soot distribution was very similar (12.5–12.7 cm) in shots fired without chokes and in shots with all extension chokes. The soot diameters obtained at 5 cm distance were analyzed by one-way analysis of variance (Table 12), and the differences between the soot diameters were not statistically significant (*p* > 0.05). The differences in soot diameters on the target surface were not statistically significant, as determined by multiple comparisons of soot diameters by choke length using the Tukey HSD test (Table 13).
- On the target, the distribution of powder particles that had burned and hadn’t was compared (Table 14). For bullets fired with and without extension, it was noted that the mean diameter of the powder particle distribution was extremely close to one another (4.89–4.99 cm). One-way analysis of variance was used to assess the distribution of diameters (Table 15), but no statistical significance was detected (*p* > 0.05). With the Tukey HSD test, multiple comparisons of the distribution diameters of gunpowder particles to extension lengths were made (Table 16), but no statistically significant differences between the distribution diameters of gunpowder particles were found because *p* > 0.05 in all comparisons.

### Shots from 25 cm distance

**Fig. 8:**
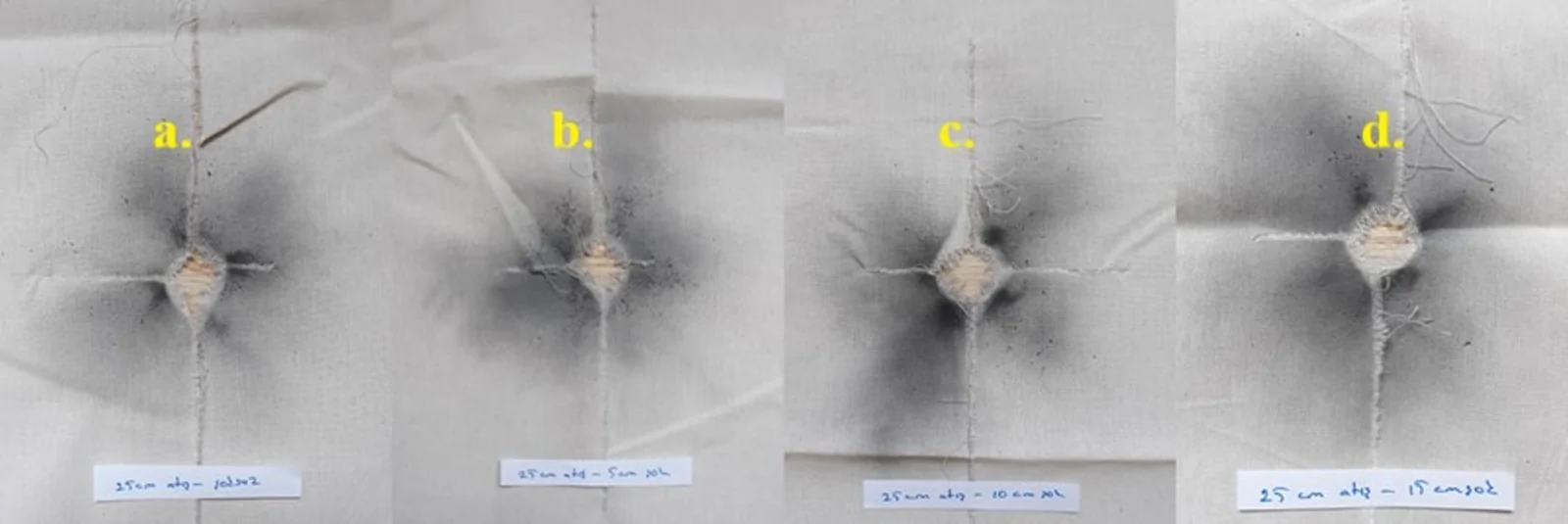
Shots from 25 cm distance: **a**.without extension **b**. with 5cm extension **c**. with 10cm extension **d**. With 15cm extension

Shots from 25 cm distance are shown in Fig. 8. All shots were observed to be homogeneous, and no satellite entry hole was detected except for the mass entry hole of the pellets.

The center entrance hole diameters for different extension lengths are compared in Table 17. It was found that the average diameter of the center entrance holes was very similar (1.94–2.01 cm) in shots without extension and in shots with all extensions. As a result of the comparisons, it was determined that the choke length had no significant effect on the center entrance hole at a distance of 25 cm. Compared to the contact shots and shots fired at a distance of 5 cm, it was observed that there was a narrowing of the entrance hole of the pellets. It was found that the pellets did not show any dispersion along the distance between the target and the exit hole of the barrel. The reason for the shrinkage in the entrance hole is that the flame and gas pressure from the muzzle exit hole is not effective on the target at a distance of 25 cm. The flame and gas pressure did not affect the entrance hole formed on the cloth target. Only the pellets’ impact caused the entrance hole.

- The one-way analysis of variance revealed that the mean diameter of the pellet entrance hole (1.94–2.01 cm) was relatively similar depending on the extension length (Table 18). It was determined that there was no statistically significant difference between the diameters (*p* > 0.05). The Tukey HSD test was used to compare pellet entrance hole diameters across barrel lengths (Table 19). The variations in pellet entry hole widths were not statistically significant because *p* > 0.05 was used in all comparisons.
- **(b)** The mean distribution of soot from the muzzle on the target (Table 20). 15.9–16.1 cm was found to be very close to each other and analyzed by one-way analysis of variance (Table 21) and was not found to be statistically significant (*p* > 0.05). Multiple comparisons of soot diameters by choke length were performed using the Tukey HSD test (Table 22). Since *p* > 0.05 in all comparisons, the differences in soot distribution diameters were not statistically significant.
- **(c)** The distribution of burnt and unburnt powder particles on the target is compared in (Table 23). It was found that the average particle diameter (13.2–13.4 cm) was very similar in shots fired without chokes and in shots fired with all extension chokes. As a result of the comparisons, it was determined that the extension chokes did not have a significant effect on the distribution of powder particles when fired at a distance of 25 cm. Due to the distance between the target and the muzzle, it was determined that the burnt powder particles were distributed over a larger area compared to the shots fired at a distance of 5 cm. One-way analysis of variance revealed that the average diameter of the distribution of burned and unburned gunpowder particles on the target was very similar (13.2–13.4 cm) and that there was no statistically significant difference between the distribution diameters (*p* > 0.05) between them (Table 24). Multiple comparisons of the powder particle distribution diameters according to barrel length were performed using the Tukey HSD test (Table 25). Since *p* > 0.05 in all comparisons, the differences were not statistically significant.

### Shots from 50 cm distance

**Fig. 9:**
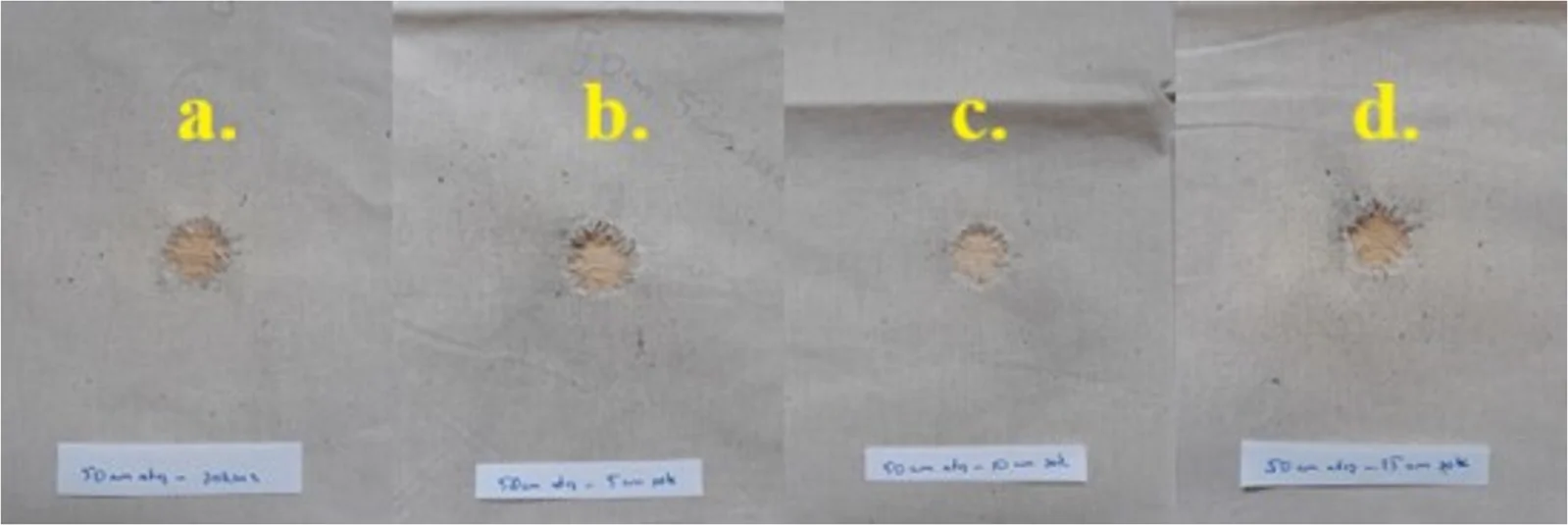
Shots from 50 cm distance: **a**. without extension **b**. with 5cm extension **c**. with 10cm extension **d**. With 15cm extension

Shots from 50 cm Distance were shown at Fig. 9 All shots were found to be homogeneous, and no satellite entry hole was detected except for the mass entry hole of the pellets.

The center entrance hole diameters for the different extension lengths are compared (Table 26). It was determined that the average diameter of the center entrance holes (1.99–2.07 cm) was very close to each other in the shots without extension and with all extensions. As a result of the comparisons, it was determined that the extension length had no significant effect on the center entrance hole at a firing distance of 50 cm. It was determined that the entrance hole characteristics of the pellets had similar characteristics to the entrance hole in the shots fired at a distance of 25 cm.

- The average diameter of the pellet entrance hole was found to be very similar across extension lengths (1.99–2.07 cm), and one-way analysis of variance did not reveal any statistically significant differences between the diameters (*p* > 0.05) (Table 27). Multiple comparisons of the pellet entry hole diameters according to the extension lengths were made with the Tukey HSD test (Table 28), and since *p* > 0.05 in all comparisons, the differences between the pellet entry hole diameters were not found statistically significant.
- All choke lengths were found to have almost the same soot distribution. It was found that the smoke spread more on the cloth target compared to rounds fired closer to the target because of the distance between the target and the muzzle of the barrel. The spread of the smoke is greater as the distance between the barrel and the cloth target grows. However, as the distance increased, the smoke’s blackness diminished until it was nearly undetectable at a 50 cm distance (Table 29). When the differences between the distribution diameters were examined using one-way analysis of variance (Table 30), it was noted that the mean diameter of the soot distribution from the muzzle on the target (19.9–20.1 cm) was very similar to one another. Multiple comparisons of soot diameters according to choke lengths were performed using the Tukey HSD test (Table 31). Since *p* > 0.05 in all comparisons, the differences in soot distribution diameters were not statistically significant.
- One-way analysis of variance revealed that the distribution of burned or unburned gunpowder particles on the target (Table 32) had a mean diameter that was very similar to one another (17.6–17.8 cm) and that there was no statistically significant difference between the distribution diameters (*p* > 0.05) between them (Table 33). Multiple comparisons of the powder particle distribution diameters according to the choke lengths were performed using the Tukey HSD Test (Table 34). Since *p* > 0.05 in all comparisons, the differences between the powder particle distribution diameters were not statistically significant.

### Shots from 75 cm distance

**Fig. 10:**
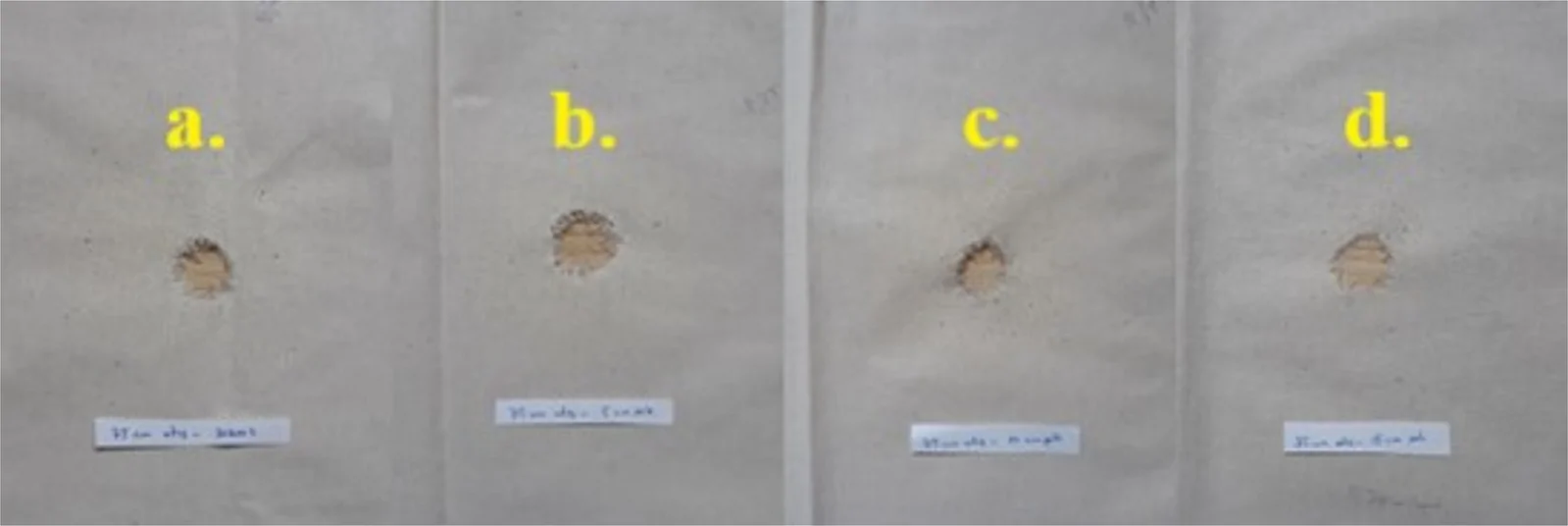
Shots from 75 cm distance: **a**.without extension **b**. with 5cm extension **c**. with 10cm extension **d**. With 15cm extension

Shots from 75 cm Distance were shown in Fig. 10. All shots were observed to be homogeneous, and no satellite entry hole was detected except for the mass entry hole of the pellets.

The center entrance hole diameters for different extension lengths are compared in Table 35. It was observed that the average diameter of the center entrance holes (2.98–3.04 cm) was very close to each other in the shots without extension and in the shots with all extensions. Based on the comparisons, it was determined that the extension length did not affect the center entrance hole at a firing distance of 75 cm. Compared to shots fired at 25 and 50-cm distances, there was a significant increase in the diameter of the entrance hole. The reason is the increased distance between the muzzle and the target. During this distance, the pellets scatter after leaving the barrel until they reach the target. However, it was determined that none of the pellets formed any satellite entrance holes other than the mass entrance hole. All pellets reached the target in an orderly manner and formed only a circular mass entry hole.

- The mean diameter of the pellet entrance hole was found to be relatively similar over the extension length range (2.98–3.04 cm), and one-way analysis of variance (Table 36) did not reveal any statistically significant variations between the diameters (*p* > 0.05). Multiple comparisons of the pellet entry hole diameters across choke lengths were performed using the Tukey HSD test (Table 37). Because *p* > 0.05 in all comparisons, the differences were not statistically significant.
- The distribution of burnt and unburnt powder particles on the target is compared in Table 38. It was found that the average particle diameter distribution was very similar (22.9–23.1 cm) in shots fired without chokes and in shots fired with all extension chokes. As a result of the comparisons, it was determined that the extension chokes had no significant effect on the distribution of powder particles when fired at 75 cm. Due to the distance between the target and the muzzle, it was determined that the burnt powder particles were distributed over a larger area compared to the shots fired from close distances. One-way analysis of variance (**Table 39)** revealed that the average diameter of the distribution of burned or unburned powder particles on the target was very similar to one another (22.9–23.1 cm) and that there was no statistically significant difference between the diameters (*p* > 0.05). Multiple comparisons of the powder particle distribution diameters according to extension length were performed using the Tukey HSD test (Table 40). Because *p* > 0.05 in all comparisons, the differences were not statistically significant.
- It was found that when fired from a distance of 75 cm, the soot did not properly reach the target regardless of the extension length. Extension chokes had no effect on soot distribution at 75 cm.

### Shots from 100 cm distance

**Fig. 11:**
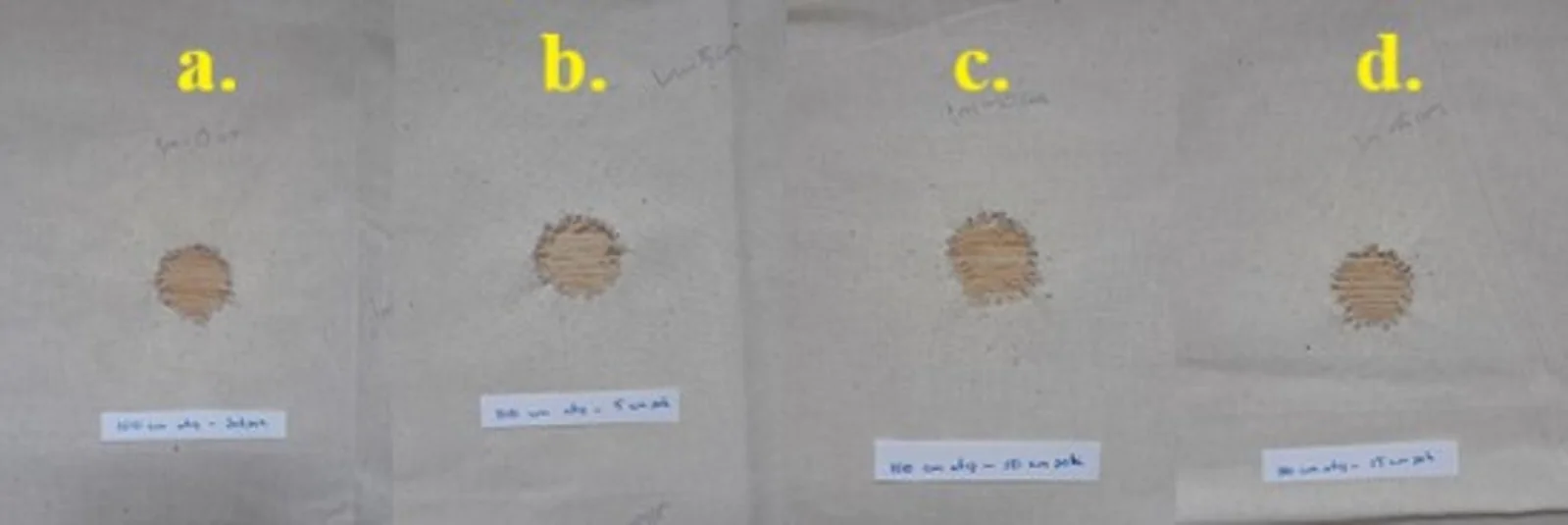
Shots from 100 cm distance: **a**. without extension **b**. with 5cm extension **c**. with 10cm extension **d**. with 15cm extension

Shots from 100 cm distance were shown in Fig. 11. All shots were observed to be homogeneous, and no satellite entry hole was detected except for the mass entry hole of the pellets.

The center entrance hole diameters for different extension lengths are compared in Table 41. It was observed that the average diameter of the center entrance holes was very close to each other (3.12–3.17 cm) in the shots without extension and in the shots with all extensions. Resulting from the comparisons, it was determined that the choke length had no significant effect on the center entrance hole at a distance of 100 cm. Compared to the 75 cm shot distances, there was a slight increase in the diameter of the entrance hole. This is due to the 25 cm increase in the distance between the muzzle and the target. As with the shots fired at 75 cm, no pellets were observed to create any satellite entrance hole other than the entrance hole. All pellets from the cartridge hit the target through a circular entrance hole.

- The average diameter of the pellet entrance hole, which ranges from 3.12 to 3.17 cm, was found to be quite similar throughout choke lengths, and one-way analysis of variance revealed no statistically significant differences between the diameters (*p* > 0.05) (Table 42). Multiple comparisons of the pellet entry hole diameters according to the extension lengths were made with the Tukey HSD test (Table 43), and since *p* > 0.05 in all comparisons, the differences between the pellet entry hole diameters were not found statistically significant.
- The distribution of burnt and unburnt powder particles on the target is compared in Table 44. It was observed that the average diameter of the powder particle distribution was very close to each other (26.9–27.3 cm) in the shots fired without chokes and in the shots fired with all extension chokes. As a result of the comparisons, it was determined that extension chokes had no significant effect on the distribution of powder particles when fired at a distance of 100 cm. Due to the distance between the target and the muzzle, it was determined that the burnt gunpowder particles were distributed over a larger area compared to the shots fired from close distances. However, the powder particles on the target were too small to be detected without sensitive observation. One-way analysis of the variance test revealed that the average diameter of the distribution of burned and unburned powder particles on the target was very similar (26.9–27.3 cm) and that there was no statistically significant difference between the diameters (*p* > 0.05) (Table 45). Multiple comparisons of the powder particle distribution diameters according to the choke lengths were performed using the Tukey HSD test (Table 46). Since *p* > 0.05 in all comparisons, the differences between the powder particle distribution diameters were not statistically significant.
- It was noted that, regardless of the extension length, soot could not successfully reach the target in rounds fired from a distance of 100 cm. It was determined that extension chokes had no impact on the soot distribution at a distance of 100 cm.

### Shots fired from a distance of 200 cm

**Fig. 12:**
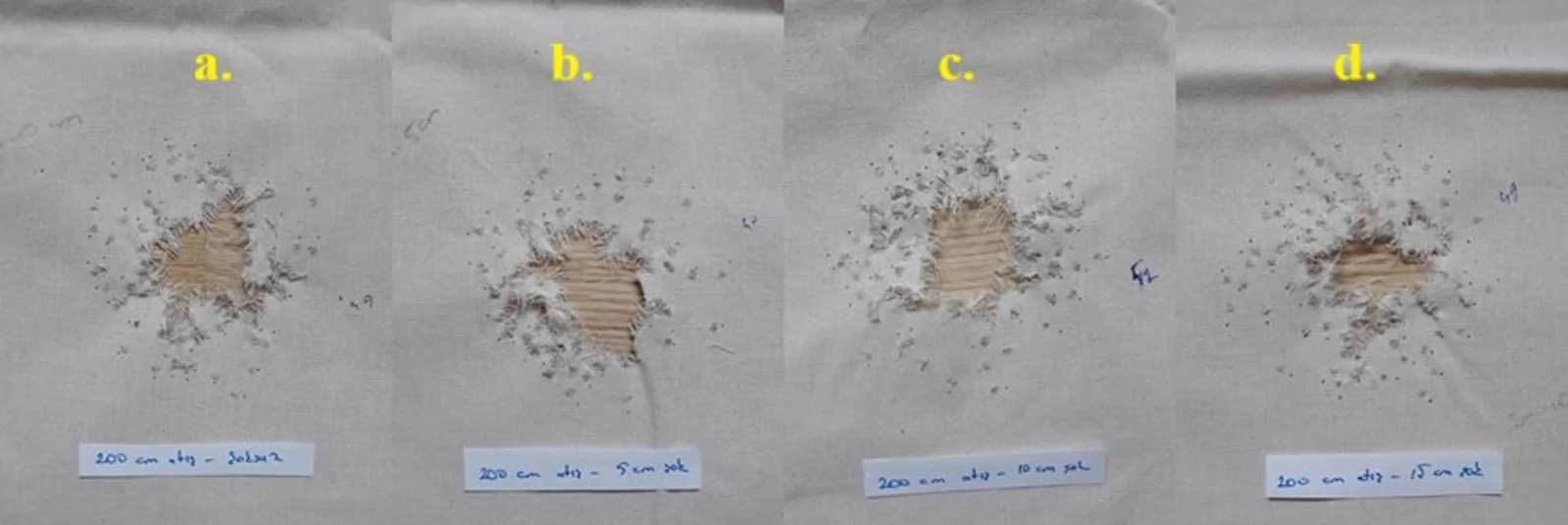
Shots from 200 cm distance: **a**.without extension **b**. with 5cm extension **c**. with 10cm extension **d**. with 15cm extension

Shots fired from a Distance of 200 cm are shown in Fig. 12. It was observed that not all the shots fired were homogeneous, and many satellite entry holes were detected, other than the mass entry hole of the pellets.

When the entry holes were examined, it was observed that the entry hole diameter decreased with increasing choke length. The average diameter of the entrance hole was measured to be 4.02 cm in shots fired without extension. This diameter fell by 0.10 cm to 3.92 cm when using a 5 cm extension, by 0.33 cm to 3.33 cm when using a 10 cm extension, and by 0.34 cm to 2.9 cm when using the longest extension, a 15 cm extension. The comparisons undertaken led to the conclusion that, when fired from a distance of 200 cm, the extensions significantly affected the diameter of the central pellet entry hole (Table 47).

- One-way analysis of variance was used to examine the diameters of the pellet entrance holes, and the differences between the diameters were determined to be statistically significant (*p* < 0,05) (Table 48). Multiple comparisons of the diameters of the pellet entry holes across choke lengths were performed using the Tukey HSD test (Table 49). The differences were not statistically significant (*p* > 0.05), even though the pellet entry hole sizes in the shots without extension were greater than those in the shots using a 5 cm extension. The diameters of 10 cm and 15 cm chokes were found to decrease as the choke length increased, and the differences in diameter sizes were found to be statistically significant (*p* < 0.05).
- The tests revealed that, at a distance of 200 cm, the choke length had very little impact on the dispersion of the pellets on the target. Between the mean pellet distribution diameters of the rounds fired without extension and the shots fired with the longest extension, the 15 cm extension, at a distance of 200 cm, there was a difference of 0.96 cm. The diameter of the pellet dispersion in shots without extension was 8.43 cm, and it was understood that this diameter shrunk as the choke length lengthened. The diameter length for an extension of 5 cm was measured at 8.03 cm, for an extension of 10 cm at 7.8 cm, and for an extension of 15 cm at 7.47 cm. It was observed that the distribution width of the pellets decreased as the choke length increased (Table 50). One-way analysis of variance was used to examine the pellet diameters (Table 51). The examination revealed that the variations in particle distribution diameters were statistically significant (p < 0.05). It was statistically shown that the particle dispersion diameter decreased with increasing choke length. The Tukey HSD test was used to compare the pellet dispersion diameters across extension lengths (Table 52). The pellet dispersion diameters were found to decrease as the extension length increased in the rounds fired with no extension, 5 cm, 10 cm, and 15 cm extension, and the decreases in diameter sizes were determined to be statistically significant (*p* < 0,05).
- The comparisons made led to the conclusion that the extension chokes affected the pellets that produced the furthest satellite entrance holes in the rounds fired at a distance of 200 cm (Table 53). In shots without extension, the distance between the farthest satellite entrance hole and the center entrance hole was 4.83 cm; in shots with a 5 cm extension, this distance was 4.53 cm, a decrease of 0.3 cm. With the 10 cm extension, the distance between the farthest satellite entrance hole and the center entrance hole was 4.3 cm, and with the 15 cm extension, it was 4.1 cm. The satellite entry hole distances shrank as the choke length grew. One-way analysis of variance was used to examine the satellite entrance holes furthest away (Table 54). The evaluation revealed statistically significant differences (*p* < 0.05) between the satellite entry holes furthest apart. Multiple comparisons of the farthest satellite entry holes by choke length were performed using the Tukey HSD test (Table 55). Although the distance of the farthest satellite entry hole was greater for shots with 5 cm extension than for shots with 10 cm extension, the differences between them were not statistically significant (*p* > 0,05). Similarly, although there was a decrease in the farthest satellite entry hole distance between shots made with 10 cm choke and those made with 15 cm choke, the difference was not statistically significant (*p* > 0, Type="Italic">05). In other comparisons, it was observed that as the extension length increased, the farthest satellite entry hole distance decreased and these differences were found to be statistically significant (*p* < 0.05).

### Shots from 300 cm distance

**Fig. 13:**
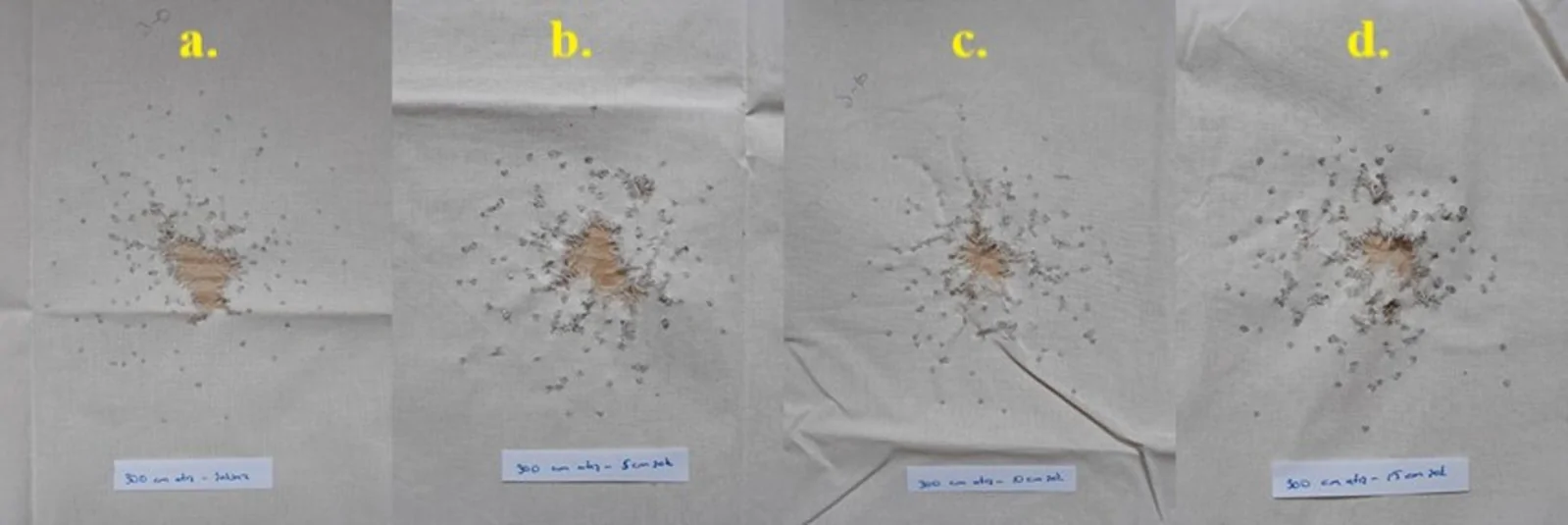
Shots from 300 cm distance: **a**.without extension **b**. with 5cm extension **c**. with 10cm extension **d**. with 15cm extension

Shots from 300 cm Distance are shown in Fig. 13. It was observed that all the shots fired were not homogeneous, and many satellite entry holes were detected other than the mass entry hole of the pellets. When the entry holes were examined, it was discovered that as the choke length rose, the entry hole’s width dropped. The entrance hole measured 3.93 cm in diameter in shots without extension, 3.17 cm in shots with a 5 cm extension, 2.33 cm in shots with a 10 cm extension, and 1.73 cm in shots with a 15 cm extension. The comparisons undertaken led to the conclusion that the diameter of the central entrance hole in rounds fired from a distance of 300 cm was significantly affected by the extension chokes (Table 56).

- The diameter of the entry holes was analyzed by one-way analysis of variance (Table 57). The differences in diameter were statistically significant (*p* < 0.05). Multiple comparisons of the diameter of the entry holes across choke lengths were performed using the Tukey HSD test (Table 58). As a result of the shots made with no choke, 5 cm, 10 cm, and 15 cm chokes, it was observed that the diameter of the entrance hole decreased as the choke length increased, and the differences in diameter sizes were found to be statistically significant (*p* < 0.05).
- The comparisons led to the conclusion that the extension length had an impact on the pellet distribution on the target at a 300 cm distance. Between the mean pellet distribution diameters of the rounds fired without extension and the shots fired with the longest extension, the 15 cm extension, there was a difference of 3.23 cm at a distance of 300 cm. Compared to shots made without the use of an extension, which had an average diameter of 14.1 cm, shots made with a 5 cm extension had an average diameter of 12.5 cm, shots made with a 10 cm extension had an average diameter of 12.1 cm, and shots made with a 15 cm extension, which is the longest extension, had the smallest diameter with a diameter length of 10.87 cm. As the choke length increased, the dispersion diameters of the pellets decreased (Table 59). One-way analysis of variance was used to examine the distribution of pellet diameters (Table 60). The analysis revealed that the variations in scatter diameters were statistically significant (*p* < 0,05). Multiple comparisons of the scatter diameters by choke length were performed using the Tukey HSD test (Table 61). Even though the diameters of the pellet distribution in the shots produced by the 5 cm extension were greater than those produced by the 10 cm extension, the differences were not statistically significant (*p* > 0.05). As the extension length increased, it was observed that the pellet dispersion diameters produced by rounds fired without extension and with a 15 cm extension decreased, and the changes in diameter were determined to be statistically significant (*p* < 0,05).
- The comparisons made led to the observation that the extension had an impact on the pellets that, when shot from a distance of 300 cm, produced the furthest satellite entrance hole. In shots without extension, the distance between the farthest satellite entrance hole and the center entrance hole was 8.8 cm; in shots with a 5 cm extension, this distance shrank by 1.13 cm to 7.67 cm. The farthest satellite entrance hole distance was measured to be 7.33 cm with the 10-cm extension and 7.1 cm with the 15-cm extension. The satellite entry hole distances shrank as the choke length grew (Table 62). One-way analysis of variance was used to examine the satellite entrance holes furthest away (Table 63). The evaluation revealed statistically significant differences (*p* < 0.05) between the satellite entry holes furthest apart. The Tukey HSD test was used to compare the furthest satellite entrance holes across choke lengths (Table 64). The differences were not statistically significant, even though the distance to the farthest satellite entry hole was shorter for extensions with a 10 cm extension than for those with a 5 cm extension (*p* > 0,05). Similar to the previous observation, it was found that the increase in extension length caused a decrease in the distances between the farthest satellite entrance hole lengths for extensions produced with a 10 cm choke and shots made with a longer 15 cm extension, but the differences between them were not statistically significant (*p* > 0,05). Other comparisons revealed that the furthest satellite entry hole distance reduced as the choke length grew, and these differences were statistically significant (*p* < 0,05).

### Shots from 400 cm distance

**Fig. 14:**
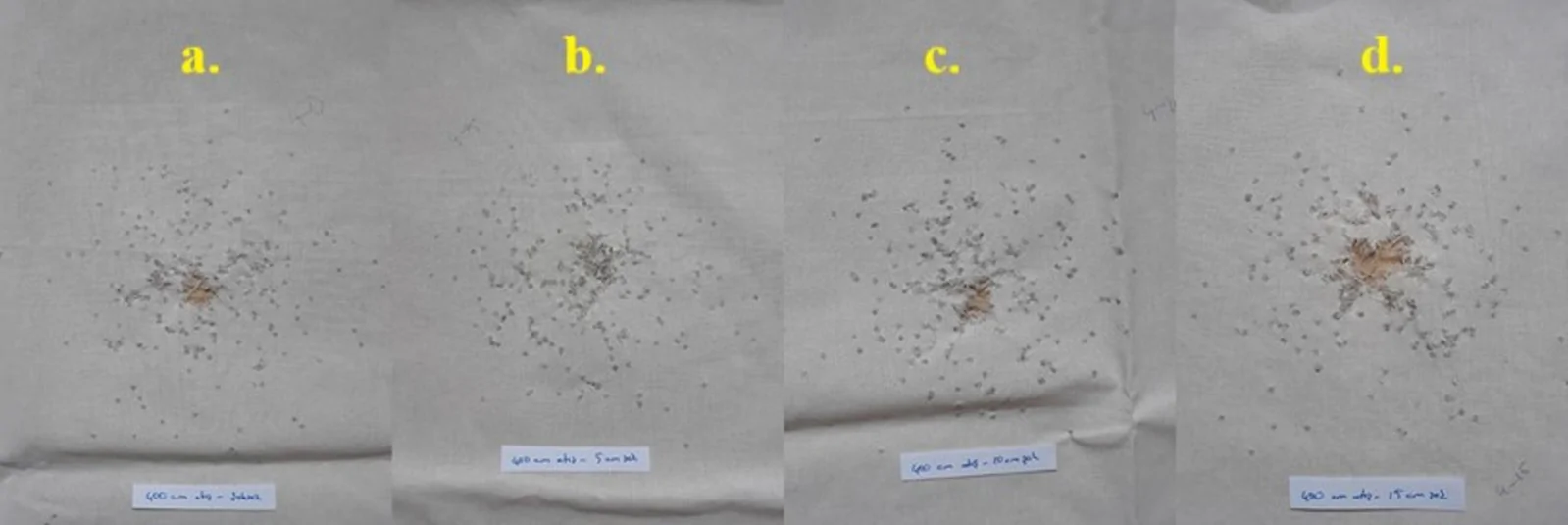
Shots from 400 cm distance: **a**.without extension **b**. with 5cm extension **c**. with 10cm extension **d**. with 15cm extension

Shots from 400 cm Distance are shown in Fig. 14. It was observed that none of the fired shots were uniform, and several satellite entry holes, in addition to the pellets’ bulk entry hole, were found. When the bulk entrance holes were examined, it was found that the entrance hole diameter decreased with increasing choke length. The comparisons led to the conclusion that, when shot at a distance of 400 cm, the extension affected the diameter of the center-pellet entry hole. In shots without extension, the entrance hole’s average diameter was 2.33 cm, but in shots with a 5 cm extension, it shrank to 1.97 cm. The average entrance hole diameter with a 10 cm extension was 1.77 cm, and with a 15 cm extension, it was 1.57 cm. It was determined that the diameter of the pellet entry hole decreased as the choke length increased (Table 65).

- The diameters of the pellet entry holes were analyzed by one-way analysis of variance (Table 66). The examination revealed that the variations in the mass entrance holes’ widths were statistically significant (*p* < 0,05).

Multiple comparisons of the diameters of the batch entry holes across choke lengths were performed using the Tukey HSD test (Table 67). The diameter of the pellet entry hole was observed to decrease with increasing extension length for the shots fired without extension using 5 cm, 10 cm, and 15 cm chokes. The variations in diameter sizes were found to be statistically significant (*p* < 0,05).

(b) The comparisons led to the conclusion that the choke length had an impact on the pellet distribution on the target at a 400 cm distance. The mean pellet distribution diameters of the rounds fired without extension and the shots fired with the longest extension, the 15 cm extension choke, differed by a total of 4.93 cm at a distance of 400 cm. In rounds without extension, the average pellet distribution diameter was 21 cm; in shots with a 5 cm extension, it was 18.07 cm; in shots with a 10 cm extension, it was 17.17 cm; and in shots with a 15 cm extension, it was 16.07 cm. The dispersion widths of the pellets decreased as the choke length increased (Table 68). One-way analysis of variance was used to examine the diameters of the pellet distribution (Table 69 Pellet Distribution Diameter Variance Analysis at 400 cm Distances Based on Extension Lengths). The evaluation revealed that the variations in pellet dispersion widths were statistically significant (*p* < 0,05). Multiple comparisons of the scatter diameters by choke length were performed using the Tukey HSD test (Table 70). Following the firing of rounds with extensions of 5 cm, 10 cm, and 15 cm, it was discovered that the pellet dispersion diameters reduced as the extension length grew, and the decreases in diameter sizes were found to be statistically significant (*p* < 0,*05).*
(c) The distances to the centers of the pellets that formed the satellite entry holes spread on the target were compared as a result of the bullets fired. The comparisons revealed that the extension chokes had no discernible impact on pellets fired from 400 cm, producing the furthest satellite entrance holes. It was found that increasing the choke length did not affect the pellets with the farthest satellite entry hole (Table 71). One-way analysis of variance was used to examine the satellite entrance holes furthest away (Table 72). The examination revealed no statistically significant difference between the satellite entry holes that were the farthest apart (*p* > 0,05).

Multiple comparisons of the farthest satellite entry holes by choke length were performed using the Tukey HSD test (Table 73). The most remote satellite entry pellets’ proximity to the center entry hole changes together with the choke length. Inconsistent differences did exist between the measured distances, but they were not statistically significant (*p* > 0,05).

### Shots fired from a distance of 500 cm

**Fig. 15:**
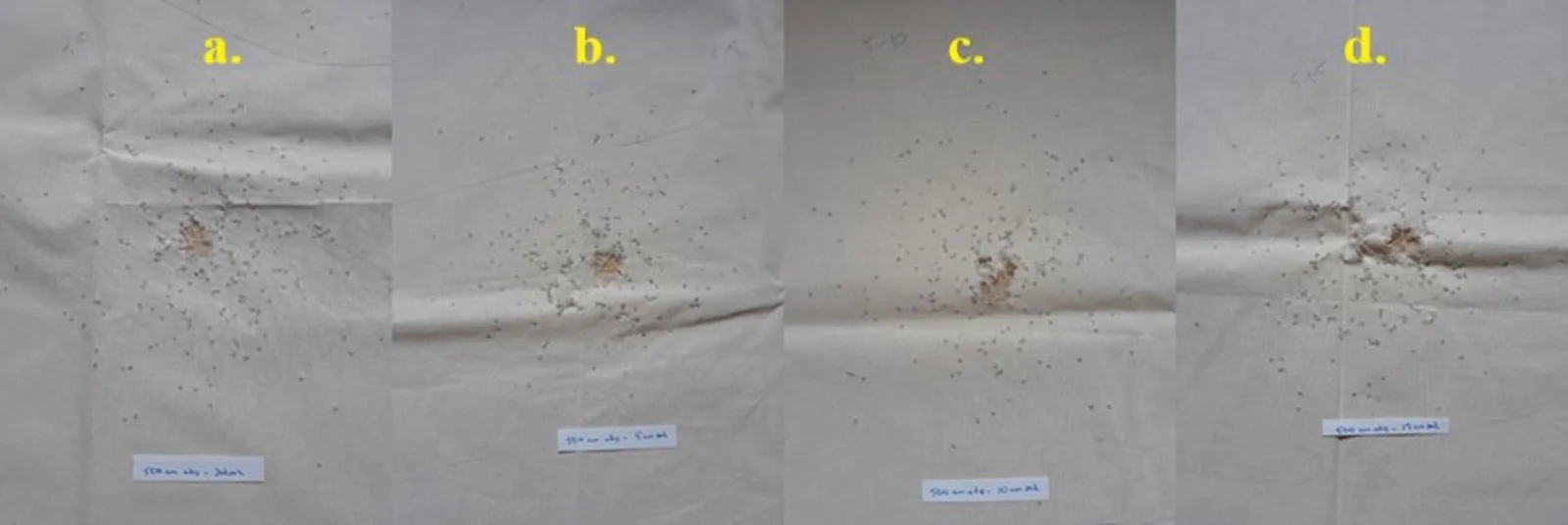
Shots from 500 cm distance: **a**.without extension **b**. with 5cm extension **c**. with 10cm extension **d**. with 15cm extension

Shots fired from a distance of 500 cm are shown in Fig. 15. It was observed that not all the shots fired were homogeneous, and many satellite entry holes were detected, other than the mass entry hole of the pellets. When the bulk entrance holes were examined, it was found that the entrance hole diameter did not decrease significantly as the choke length increased. The comparisons conducted led to the conclusion that, when fired at a distance of 500 cm, the extension had no discernible effect on the diameter of the central pellet entry hole. The difference in the diameter of the pellet entry hole between the shots fired without choke and the shots fired with the longest choke, the 15 cm extension choke, is only 0.25 cm (Table 74).

- One-way analysis of variance was used to examine the entrance hole diameters (Table 75). The evaluation revealed that the diameter variations between the mass entrance holes were statistically significant (*p* < 0,05). Multiple comparisons of the diameters of the pellet entry holes across choke lengths were performed using the Tukey HSD test (Table 76). As a result of the shots fired without extension and with a 5 cm extension, it was observed that the diameter of the pellet entry hole decreased as the choke length increased, but the differences between them were not statistically significant (*p* > 0,*05).* Comparisons between 5 cm, 10 cm, and 15 cm extensions showed that the diameter of the pellet entry holes decreased with increasing extension length, but the differences were not statistically significant (*p* > 0,05). The diameter of the pellet entry holes was found to be statistically significant in a comparison of shots done without extension and with 10 cm and 15 cm extensions (*p* < 0,05).
- The comparisons led to the conclusion that the extension length had an impact on the pellet distribution on the target at a 500 cm distance. Between the mean pellet distribution diameters of the bullets fired without extension and the ones fired with the longest extension, the 15 cm extension choke, there was a difference of 5.65 cm at a distance of 500 cm. In the bullets that were fired without extension, the average diameter of the pellet distribution was 25.57 cm. The average pellet distribution diameter measured 23.2 cm with a 5 cm extension, 21.6 cm with a 10 cm extension, and 19.92 cm with a 15 cm extension. The dispersion width of the pellets decreased as the extension length increased (Table 77). One-way analysis of variance was used to examine the pellet diameters (Table 78). The evaluation revealed that the variations in scatter diameters were statistically significant (*p* < 0,05). Multiple comparisons of pellet distribution diameters across choke lengths were performed using the Tukey HSD test (Table 79). The pellet dispersion diameters were found to decrease as the extension length increased in the shots fired with no extension, 5 cm, 10 cm, and 15 cm extensions. The declines in diameter sizes were found to be statistically significant (*p* < 0,05).
- The distances to the centers of the pellets that formed the satellite entry holes spread on the target were compared as a result of the bullets fired. The comparisons revealed that the extension chokes had no impact on the pellets that produced the farthest satellite entry hole in shots fired from a distance of 500 cm, and that the distances between the entry holes were uneven. It was determined that increasing the choke length did not affect the pellets making the farthest satellite entry hole (Table 80). One-way analysis of variance was used to examine the satellite entrance holes furthest away (Table 81). The examination revealed no statistically significant difference between the satellite entry holes that were the farthest apart (*p* > 0,05). Multiple comparisons of the farthest satellite entry holes by extension length were performed using the Tukey HSD test (Table 82). Depending on the length of the extension, the distance between the furthest satellite entry pellets and the center entry hole changes. However, there was no statistically significant difference between the satellite entrance hole distances (*p* > 0,05).

### Shots from 700 cm distance

**Fig. 16:**
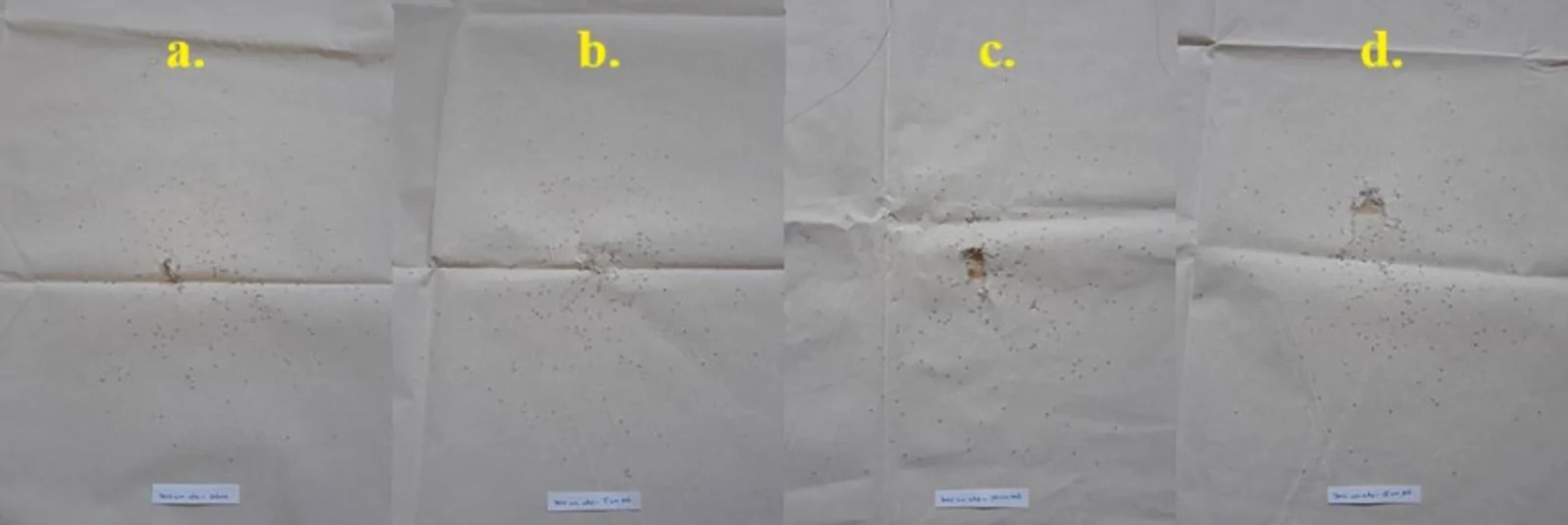
Shots from 700 cm distance: **a**.without extension **b ** with 5cm extension **c**. with 10cm extension **d**. with 15cm extension

Shots from 700 cm Distance were shown in Fig. 16 It was noted that none of the fired shots were uniform, and several satellite entry holes, in addition to the pellets’ bulk entry hole, were found. When the bulk entrance holes were examined, it was found that the entrance hole diameter did not decrease significantly with increasing extension length. The comparisons conducted led to the conclusion that, when fired at a distance of 700 cm, the extension had no effect on the diameter of the central pellet entry hole. The average diameter of the pellet entry hole was found to be between 2.2 cm and 2.3 cm, and it did not differ substantially between shots with or without extensions of 5–10-15 cm (Table 83).

- One-way analysis of variance was used to examine the borehole diameters (Table 84). The study revealed no statistically significant difference in the diameters of the mass entrance holes (*p* > 0,05). Multiple comparisons of the diameters of the pellet entry holes according to the extension lengths were made with the Tukey HSD test (Table 85). The diameter of the pellet entry holes did not change significantly as the extension length increased, and the differences between the no extension, 5 cm, 10 cm, and 15 cm extensions were not statistically significant (*p* > 0,05).
- The comparisons led to the conclusion that the extension length had an impact on the pellet distribution on the target at a 700 cm distance. The average diameter of the pellet distribution in rounds fired without extension and the average diameter of the pellet distribution in shots fired with the longest choke, a 15 cm extension, were found to differ by 8.51 cm at a distance of 700 cm. In bullets fired without extension, the average diameter of the particle distribution was 37.58 cm. The average diameter of the pellet dispersion for a 5 cm extension was 33.48 cm, for a 10 cm extension, it was 31.42 cm, and for a 15 cm extension, it was 29.07 cm. The dispersion diameters of the pellets decreased as the extension length increased (Table 86). One-way analysis of variance was used to examine the pellet diameters (Table 87). The evaluation revealed that the variations in pellet dispersion widths were statistically significant (*p* < 0,05). Multiple comparisons of pellet distribution diameters according to extension lengths were made with the Tukey HSD test (Table 88). The pellet dispersion diameters decreased as the choke length increased, and the decreases were determined to be statistically significant, as a result of rounds fired without extension and with 5 cm, 10 cm, and 15 cm extensions (*p* < 0,05).

The distances to the centers of the pellets that formed the satellite entry holes spread on the target were compared as a result of the bullets fired. After comparing the results, it was found that the extension chokes had no impact on pellets fired from 700 cm, and that the furthest satellite entry hole was formed. The pellets with the furthest satellite entry hole were found to be unaffected by the length of the extension (Table 89). One-way analysis of variance was used to examine the satellite entrance holes furthest away (Table 90).

(c) ). The examination revealed that there was no statistically significant difference between the satellite entry holes that were the farthest apart (*p* > 0,05). Multiple comparisons of the farthest satellite entry holes by extension length were performed using the Tukey HSD test (Table 91). Depending on the length of the extension, the distance between the furthest satellite entry pellets and the center entry hole changes. However, there was no statistically significant difference between the satellite entrance hole distances (*p* > 0,05).

### Shots from 800 cm distance

**Fig. 17:**
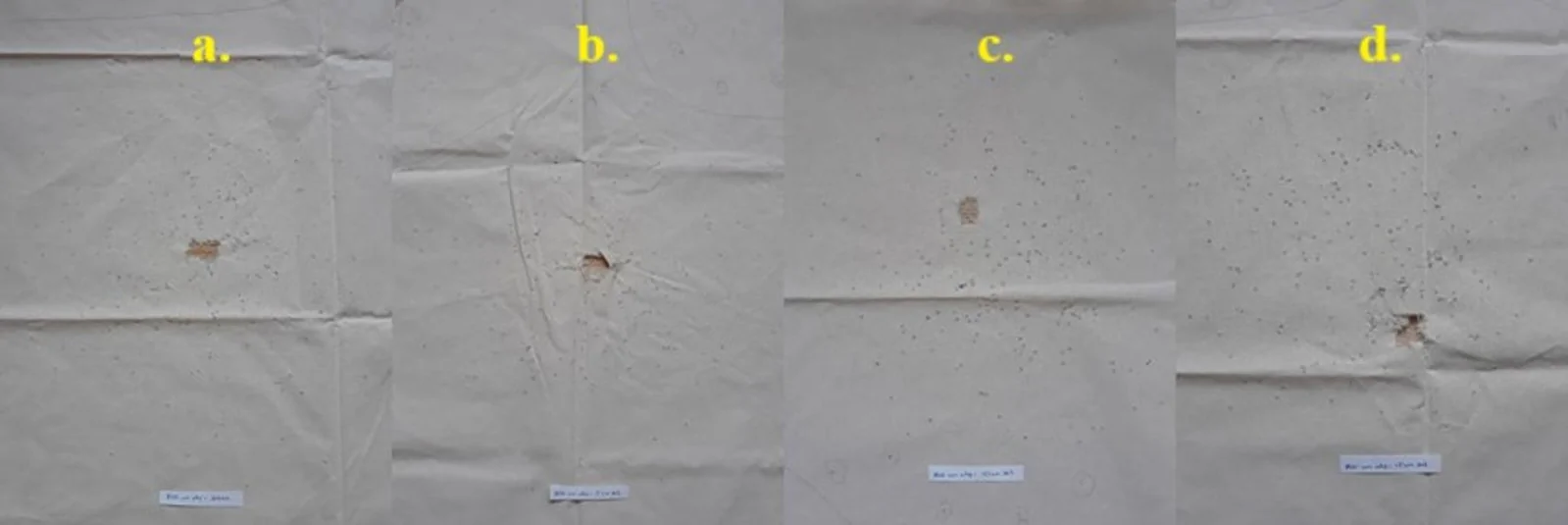
Shots from 800 cm distance: **a**.without extension **b**. with 5cm extension **c**. with 10cm extension **d**. With 15cm extension

Shots from 800 cm Distance are shown in Fig. 17. It was observed that not all shots fired were homogeneous, and many satellite entry holes were detected, in addition to the mass entry hole of the pellets. When the bulk entrance holes were investigated, it was found that as the extension length increased, the entrance hole width did not change appreciably. The comparisons revealed that when fired from a distance of 800 cm, the extension had no impact on the diameter of the center pellet entry hole. In shots made both with and without any extensions of various lengths, it was found that the average diameter of the pellet entry hole was 2.23 cm (Table 92).

- One-way analysis of variance was used to examine the borehole diameters (Table 93). The study revealed no statistically significant difference in the diameters of the mass entrance holes (*p* > 0,05). Multiple comparisons of the diameters of the pellet entry holes according to the extension lengths were made with the Tukey HSD test (Table 94). The diameter of the pellet entry holes did not change significantly as the choke length increased, and the differences between the no extension, 5 cm, 10 cm, and 15 cm extensions were not statistically significant (*p* > 0,05).
- The comparisons led to the conclusion that the extension length had an impact on the pellet distribution on the target at an 800 cm distance. Between the mean pellet distribution diameters of the rounds fired without extension and the ones fired with the longest extension, the 15 cm extension, there was a difference of 9.08 cm at a distance of 800 cm. In bullets fired without chokes, the average diameter of the particle distribution was 45.48 cm. The diameter of the pellet dispersion was 42.63 cm with a 5 cm extension, 41.13 cm with a 10 cm extension, and 36.4 cm with a 15 cm extension. It was determined that as the choke length increased, the dispersion diameters of the pellets decreased (Table 95). Pellet distribution diameters were analyzed by one-way analysis of variance (Table 96). The evaluation revealed that the variations in scatter diameters were statistically significant (*p* < 0,05). Multiple comparisons of the scatter diameters by extension length were performed using the Tukey HSD test (Table 97). As a result of the shots fired without extension, using 5 cm, 10 cm, and 15 cm extensions, it was observed that as the extension length increased, the pellet distribution diameters decreased, and the decreases in diameter were statistically significant (*p* < 0,05).
- The distances to the centers of the pellets that formed the satellite entry holes spread on the target were compared as a result of the bullets fired. As a result of the comparisons made, it was determined that the extensions did not affect the pellets that made the farthest satellite entry hole when fired from a distance of 800 cm. It was found that increasing the extension length did not affect the pellets with the farthest satellite entry hole (Table 98). One-way analysis of variance was used to examine the satellite entrance holes furthest away (Table 99). The examination revealed no statistically significant difference between the satellite entry holes that were the farthest apart (*p* > 0,05). Multiple comparisons of the farthest satellite entry holes by choke length were performed using the Tukey HSD test (Table 100). It was discovered that the length of the extension employed affected the distance between the furthest satellite entrance pellets and the central entry hole. However, there was no statistically significant difference between the locations of the satellite entrance holes (*p* > 0,05).

### Shots from 1000 cm distance

**Fig. 18:**
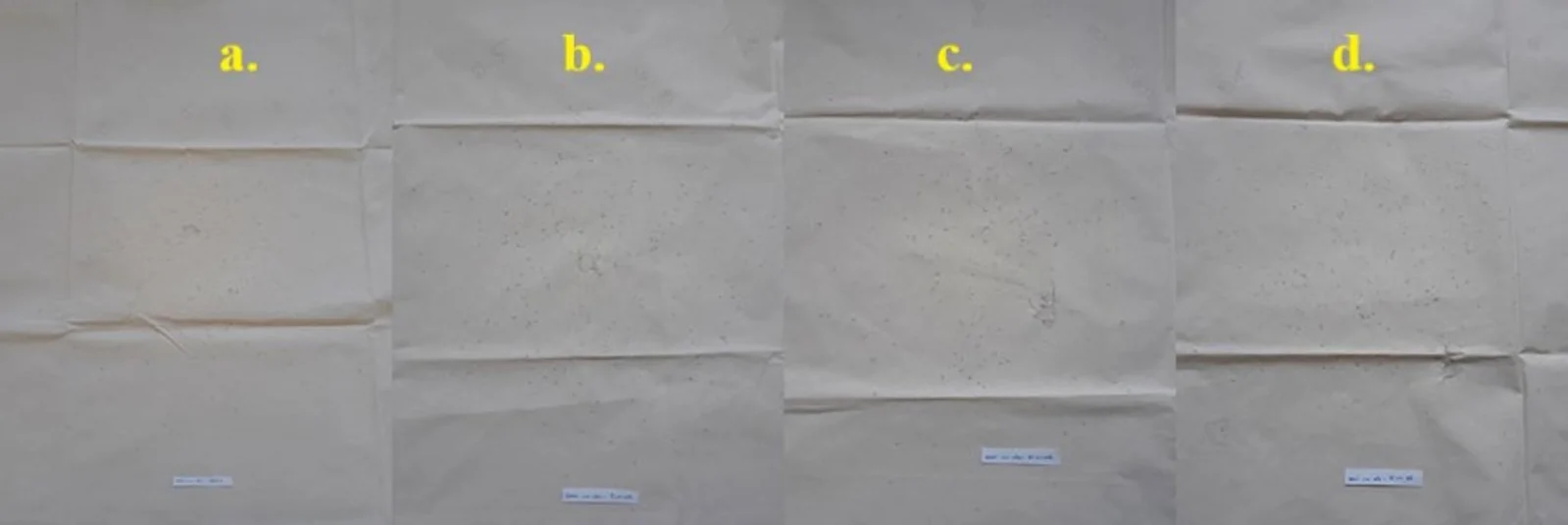
Shots from 1000 cm distance: **a**.without extension **b**. with 5cm extension **c**. with 10cm extension **d**. with 15cm extension

Shots from 1000 cm Distance are shown in Fig. 18. It was noted that none of the fired shots were uniform, and several satellite entry holes, in addition to the pellets’ bulk entry hole, were found. When the bulk entrance holes were investigated, it was found that as the extension length increased, the entrance hole width did not change appreciably. The comparisons made allowed it to be determined that, when fired from a distance of 1000 cm, the extensions had no impact on the diameter of the center pellet entry hole The average diameter of the pellet entry hole, which had an average diameter of 2.2 cm, was found to be roughly constant throughout all shots fired with and without extensions (Table 101).

- One-way analysis of variance was used to examine the borehole diameters (Table 102). The study revealed no statistically significant difference in the diameters of the mass entrance holes (*p* > 0,05).

Multiple comparisons of the diameters of the pellet entry holes were made with the Tukey HSD test (Table 103). The diameter of the pellet entry holes did not change significantly as the extension length increased, and the differences between the no extension, 5 cm, 10 cm, and 15 cm extensions were not statistically significant (*p* > 0,05).

(b) In all shots, it was also determined that there were satellite entry holes around the center mass entry hole, formed by the pellets fired from the center. As a result of the comparisons, it was determined that the extension length affects the pellet distribution on the target at a distance of 1000 cm. Between the mean pellet distribution diameters of the rounds fired without extension and the ones fired with the longest extension, the 15 cm extension, there was a difference of 10.15 cm at a distance of 1000 cm. In the shots that were fired without extension, the average diameter of the pellet distribution was 52.55 cm. The diameter of the pellet dispersion was 48 cm with a 5 cm extension, 46.27 cm with a 10 cm extension, and 42.4 cm with a 15 cm extension. The dispersion width of the pellets shrank as the extension length increased (Table 104).

One-way analysis of variance was used to examine the pellet diameters (Table 105). The evaluation revealed that the variations in scatter diameters were statistically significant (*p* < 0,05). Multiple comparisons of the scatter diameters by extension length were performed using the Tukey HSD test (Table 106). The pellet dispersion diameters decreased with increasing extension length following 5 cm and 10 cm chokes; however, the decreases were not statistically significant (*p* > 0,05). In other comparisons, it was found that the pellet dispersion diameter decreased as the extension length increased, and the changes in diameter were statistically significant (*p* < 0,05).

(c) The distances to the centers of the pellets that formed the satellite entry holes spread on the target were compared as a result of the bullets fired. The comparisons revealed that the extensions had no impact on pellets fired from 1000 cm, which produced the furthest satellite entrance holes. The pellets with the farthest satellite entrance hole (Table 107) are unaffected by lengthening the extension. One-way analysis of variance was used to examine the satellite entrance holes furthest away (Table 108). The examination revealed no statistically significant difference between the satellite entry holes that were the farthest apart (*p* > 0,05). Multiple comparisons of the farthest satellite entry holes by extension length were performed using the Tukey HSD test (Table 109). It was discovered that the length of the extension employed affected the distance between the furthest satellite entrance pellets and the central entry hole. However, there was no statistically significant difference between the locations of the satellite entrance holes (*p* > 0,05).

**Fig. 19:**
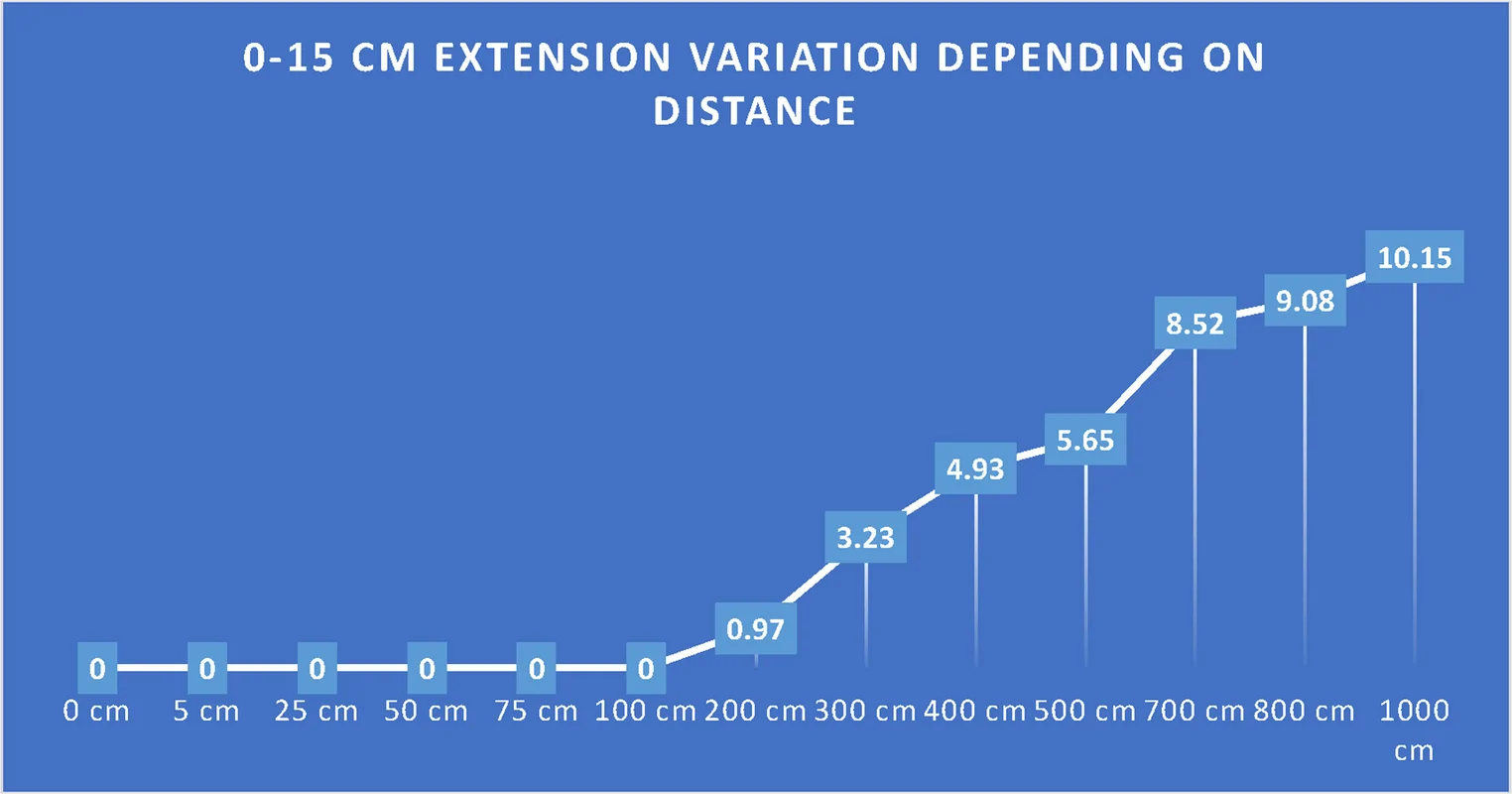
The amount of reduction in pellet dispersion diameters in shots fired without choke and with a 15 cm extension choke, depending on the distance

The extent of the reduction in pellet dispersion diameters observed in shots fired without a choke and with a 15 cm extension choke, as a function of shooting distance, is shown in Fig. 19.

## Discussion

The type of firearm used in the incident, the type of bullet or cartridge used, and the distance at which the shot was fired must all be identified to complete the judicial procedure in situations of damage and death brought on by the use of guns. Determining the firing distance is not always easy. If the products of gunpowder combustion can be detected on the target, it can be concluded that the shot was fired at close range. However, if such findings cannot be obtained, it is understood that the shot was fired from a distance and that it is not possible to determine exactly how far it was fired.

Shotguns, which are long-barreled weapons among firearms, are widely used in cases of wounding, homicide, and suicide because they can be easily obtained legally in our country. In cases of suicide with shotguns, it is determined that the shooting distance is usually contact or close to contact shots based on the shot residues. In homicides, on the other hand, it is seen that shots are fired at close range in addition to the usual shooting distance.

420 instances were reported to have involved gun injuries in research by Cantürk et al. [14] that covered 2410 autopsies conducted between January 1, 2002, and December 31, 2004, in the Department of Morgue Specialization of the Ankara Forensic Medicine Institute. It is reported that 138 of the deaths due to firearm injuries were suicides, 96 of which were caused by short-barreled firearms, and 42 of which were caused by long-barreled firearms. In 116 cases, gunshot wounds were inflicted from contact or near-contact firing distances, 95 of which were inflicted with short-barreled and 21 with long-barreled firearms. It is reported that 27 bullet entry wounds, 6 of which were caused by short-barreled and 21 by long-barreled firearms, were caused by shots fired at close range.

Ozdes et al., [15] examined 50 cases sent to the 4th Specialized Board of the Forensic Medicine Institute by the judicial authorities, who were alleged to have committed crimes with firearms, and a forensic psychiatric examination was requested in terms of criminal responsibility. When the firearms used by 48 male (17–77 years old) and 2 female (42 and 49 years old) respondents were analyzed, it was determined that 27 used pistols, 23 used smoothbore shotguns, and none used rifled hunting shotguns. 33 cases used unlicensed firearms and only 6 of them had a gun license. In 22 cases, perpetrators had no penal responsibility.

When using a shotgun rather than other firearms, it is possible to estimate the shooting distance by observing how the pellets are distributed on the target. The dispersion of pellets is affected by a wide range of conditions, though. The distribution of pellets on the target is influenced by several variables, including the shotgun’s barrel length, shot condition, pellet structure, and shot cartridge qualities. Without knowing these details, determining a reliable shooting distance is quite challenging.

Asirdizer et al. [16] reviewed 36,971 autopsy reports issued by the Forensic Medicine Institute Morgue Specialization Department between 1998 and 2007 and determined that 73 cases of suicidal deaths in Istanbul occurred with hunting shotguns. When evaluated by firing distance, 63 cases were from contact and 10 from near-contact distances.

Agir, [17] evaluated the autopsies of 114 cases of shotgun-related deaths that were submitted to the Istanbul Forensic Medicine Institute for 4 years starting from 1990. He determined that 11 of these cases were caused by shotguns, and 1/3 of the total number of cases were caused by distant shooting. He reported that 5 of these cases were not contact shots, and 17 were cases where the distance could not be determined.

To determine distance from pellet distribution in shotgun shots, it is necessary to conduct experimental studies that account for all possible variables. In this study, the effects on pellet distribution were examined by using extension chokes attached to the end of the barrel in shotguns. Thus, it is aimed to form a part of the experimental studies to be carried out using all variables and it is thought that the results obtained can be used in practice.

A 12-gauge, semi-automatic shotgun measuring 71 cm in length and number 6, 30-grain hunting ammunition were employed in the investigation. Three rounds were fired at the cloth target, with no extension and extensions of 5, 10, and 15 cm in the first stage. The holes left on the cloth target after the rounds were fired were examined. It was observed that all 12 shots fired in total were the same. When the mass entry holes of the pellets were examined, it was found that all of them were very close to each other. In addition, it was determined that the soot distribution diameters on the target were similar in all shots. The results were also evaluated by an ANOVA test, and no statistically significant difference was found (*p* > 0.05). It was understood that extension chokes did not affect results from shots made at the contact shooting distance.

The same shots were fired at distances of 5, 25, and 50 cm. It was observed that as the distance increased, the dispersion diameter of the powder soot widened. Burnt gunpowder particles were also observed on the target. In addition, it was determined that the entrance hole of the pellet was enlarged by approximately 0.65 cm when fired from a distance of 5 cm compared to the shots fired from the contact firing distance. The gas pressure and the flame emerging from the muzzle of the barrel had a wider impact on the target. As a result, a larger entry hole was opened. The effects of extension chokes on pellet distribution and shot residuals were not found to be statistically significant (*p* > 0.05) in the shots fired from 5, 25 and 50 cm distances and it was concluded that they had no effect. At distances of 75 and 100 cm, smoke soot did not affect the target. Due to the increase in the distance, it was observed that there was an increase in the diameters of the pellet entry hole and the distribution diameters of the gunpowder particles on the target. However, this increase in diameter was not statistically significant. It was determined that extension chokes did not affect the diameter of the entrance hole and powder dispersion at a distance of 75 and 100 cm (*p* > 0.05).

Tugcu, [18] shot cattle skin with a semi-automatic pistol from different distances between 0 and 60 cm. At the end of the shots, she reported that shot residues were observed along the trajectory and that they decreased and disappeared as the distance increased. Sarıbey, [19] reported that when he examined the entrance holes of the shots he made with pistols at the cloth ground target, if there were no shot residues around the hole, the shooting distance was more than 50 cm. In rifles, he states that the same situation is valid from a distance of 1 m. Similarly, in this study, combusted gunpowder particles was observed on the target surface at distances up to 50 cm. In all shots up to 100 cm, burnt gunpowder particles were observed on the target.

Knight, [8] states that pellets with single-satellite entrance holes, other than the mass entrance hole, are observed in shots beyond 100 cm. Çelikel, [20] shot at different distances with shotguns with different barrel lengths. As a result of his shots, he observed that the pellet distribution increased as the barrel length shortened. He reported that satellite entry holes occurred in shots at distances greater than 1 m. In this study, no satellite entry hole was observed except for the mass entry hole in the shots fired from the contact distance to a distance of 100 cm. At distances over 100 cm, it was observed that satellite entry holes occurred in addition to the mass entry hole.

In our study, we found that the entrance hole diameter decreased as the choke length increased in shots fired from a distance of 200 cm. In addition, satellite entry holes were also formed outside the central mass entry hole. It was observed that the pellet distribution diameter decreased as the choke length increased. However, it was also observed that the distance to the satellite entrance hole, formed at the farthest point from the central mass, decreased. At a distance of 200 cm, the effects of choke length on pellet distribution were found to be statistically significant (*p* < 0.05).

In our research, we found that the diameter of the pellet entry hole in shots fired at 300 cm was similar to that in shots fired at 200 cm. It was found that the diameter of the entrance hole and the diameter of the pellet distribution decreased as the choke length increased. However, it was observed that the distance from the center to the farthest satellite entry hole decreased with increasing choke length. At 300 cm distance, the effects of choke length on pellet distribution were found to be statistically significant (*p* < 0.05).

In Meric’s thesis [21], he shot with a 12-gauge shotgun with a 71 cm barrel and cartridges containing different types of grains (bulgur, wheat, and vetch seeds). It is stated that the diameter of the central entrance hole decreases after a distance of 100 cm. At a distance of 300 and 500 cm, no significant difference was found between the central entrance hole diameters. He reported that the diameter of the central exit hole was equal to that of the cartridge fuse. In this study, it was reported that there was no statistically significant difference in the diameter of the central entrance hole between shots fired at 300 cm and shots fired at the cartridge fuse diameter.

In our study, it was observed that the farthest satellite entry hole did not change depending on the choke length and irregular situations occurred in all shots fired from a distance of 400 cm. No statistically significant difference was found (*p* > 0.05). However, it was found that the pellet distribution diameters on the target decreased as the choke length increased. The differences between the pellet distribution diameters were statistically significant (*p* < 0.05).

Yucel, [22] shot with a shotgun with a 71 cm barrel, using different numbered cartridges at different distances. As a result of the shots, the central entrance hole is observed up to 2–6 m, and satellite entrance holes are observed beyond 1 m. They also found that the cartridge fuse entered the target through the central entrance hole or the center of pellet dispersion up to a distance of 15 m. Similarly, in this study, it is stated that the cartridge fuse penetrates the target in all shots up to 10 m, and the hole where the fuse enters the target is at the center of the pellet dispersion.

In our study, the characteristics of the central mass entry holes and satellite entry holes at the farthest distance were similar across shots fired from 500, 700, 800, and 1000 cm. It was found that it did not change with the choke length used (*p* > 0.05). At 4 distances, the diameters of the central mass entry holes were found to be similar, approximately equal to the cartridge fuse diameter. It was understood that the distance of the farthest satellite entry holes to the center did not depend on the length of the choke; there was no statistically significant difference (*p* > 0.05). However, the dispersion diameters of the pellets on the cloth target decreased with increasing choke length for shots fired at distances of 500, 700, 800, and 1000 cm, and the differences were statistically significant (*p* < 0.05). It was determined that the choke length affected the pellet dispersion diameters.

Arslan, [23], in his study using 12- and 16-caliber shotguns with a barrel length of 71 cm and number 2 and number 5 pellets, reported that pellet distributions increased statistically significantly at distances of 100 cm or more. It is stated that the pellet distribution diameters decrease in shots fired with a mobile choke.

Deniz, [24] fired 12 calibers with a 55 cm barrel length, with cylindrical, half, and full chokes. He compared the pellet distributions with the shots. It is stated that the pellet distribution increases with distance and decreases with increasing choke level. Similar results were obtained in our study, which was conducted with no choke, mobile choke, and extension choke. As distance increased, pellet distribution increased; as choke length increased, pellet distribution decreased.

Bilgi, [25] reported that in shots fired with shotguns with barrel lengths of 51, 41, and 31 cm from a distance of 150 cm using No. 0 pellets, mass entry holes with several satellite entry holes around them were detected. With 21 and 11 cm barrel lengths, many satellite entry holes were detected. With 51 and 21 cm barrel lengths, mass entry holes with several satellite entrances were detected when shot from a distance of 100 cm with number 9 pellets. When the 11 cm barrel was used, a large number of satellite entry holes were observed, and when shots were fired with a 1 cm barrel, scattered single pellet entry holes without a mass entry hole were detected, and when shots were fired closer than 1 m, no satellite entry holes other than the mass entry hole were detected. It was reported that pellet distribution increased as the barrel of the shotgun became shorter and decreased as it became longer.

In Deniz’s (2009) study, shots were fired at various distances with 12-gauge shotguns with barrel lengths of 55 cm and 71 cm. After a distance of 150 cm, it is stated that the average pellet distribution is higher in shots fired with a 55 cm barrel and that the pellet distribution increases as the barrel length shortens. In our study, the barrel length was increased by using an extension choke. Between 200 and 1000 cm, it was determined that the pellet dispersion decreased with increasing barrel length. The decrease in the amount of pellet dispersion was found to be statistically significant (*p* < 0.05).

Cakir (1997) shot at different distances with different cartridge numbers and found that the cartridge number affected the pellet distribution. He reported that the larger the pellet number, i.e. the smaller the diameter of the pellets, the higher the dispersion. In this study, number 6 cartridges containing approximately 225 pellets were used in all shots. Since different cartridges contain different numbers of pellets, pellet distributions will vary.

Karapirli, [26] conducted a study using a shotgun with a 70 cm barrel and cartridges numbered 0–9. In his study, he placed intermediate targets between the barrel and the target and examined their effects on pellet dispersion. His research found that intermediate targets increased pellet dispersion. In his study with aluminum, tin, and cotton fabric, he reported that as the density of the intermediate target increased, the pellet dispersion increased. In our study, there was no intermediate target between the barrel and the target. After the shots, the pellets hit the fabric target directly. There are no intermediate targets that affect the pellet distribution and the distribution of powder combustion products on the target.

## Conclusion

The extension chokes had no impact on the pellet distributions from the closer shooting distance up to 100 cm, according to a general evaluation. As the choke length increased, the particle dispersion diameters decreased significantly in rounds fired at distances of 200, 300, 400, 500, 700, 800, and 1000 cm. The differences in pellet diameter between rounds fired from a shotgun without choke and those fired from a shotgun with a 15 cm extension choke at all tested ranges were compared.

As a result, it was found that when shot from a distance greater than 100 cm, the particle dispersion diameters decreased as the choke length increased. The rounds fired from the same distance and displayed in the tables had the least diameter breadth, with a 15 cm-long choke. The findings and charts acquired will help calculate the distance because it is believed that the extension chokes used with shotguns are a device that impacts the pellet distributions in shotgun-related injuries and fatalities.

The dispersion of the pellets will be affected by external factors when determining the firing distance. It should be considered that the shooter might move about, which could cause the pellets to miss the target after being fired.

The pellet dispersion will suffer if there are intermediate targets between the target and the weapon. The mistake rate will rise if satellite entry holes on the target are not accurately detected. To avoid getting the wrong answers when calculating the shooting distance, intermediate targets that might affect the shot should be taken into account.

In our study, only No. 6 30 gr cartridges were used in all shots. The cartridge number and pellet diameter must be taken into consideration when determining the firing distance based on the pellet distribution.

In our study, 12-gauge shotguns and extension chokes of 5, 10, and 15 cm were used. Therefore, it is not appropriate to use the data obtained in this study when different types of shotguns and different choke lengths are used. Therefore, it would be useful to conduct similar studies using different types of shotguns, different choke lengths, and different types of shotshells. It is thought that creating standardized charts that include all parameters affecting pellet distributions would be useful. It would be useful to conduct similar studies using different types of shotguns, shot lengths, and cartridge numbers than those used in this study.

## Supplementary Information

Below is the link to the electronic supplementary material.

Supplementary Material 1 (DOCX 402 KB)
