# Supplementary material for: The effect of shotgun barrel extension chokes on pellet dispersion and shooting distance determinations
Source: Int J Legal Med. 2026 Jun 8;140(5):2989–3009. doi: 10.1007/s00414-026-03858-2 (PMC13499784; doi:10.1007/s00414-026-03858-2)
Supplement: Supplementary file 1 [file 414_2026_3858_MOESM1_ESM.docx]

**Table 2** Entry hole diameters based on extension length at adjacent ranges.

| Measurement  Type | Extension  Length | Number  Of  Shots | Arithmetic Mean  (cm) | Standard Deviation | Standard Error | Confidence Interval (%95)  Lower Limit | Confidence Interval (%95)  Upper Limit |
| --- | --- | --- | --- | --- | --- | --- | --- |
| Horizantal | No extension | 3 | 2,37 | 0,058 | 0,034 | 2,31 | 2,44 |
|  | 5 cm | 3 | 2,34 | 0,058 | 0,034 | 2,27 | 2,4 |
|  | 10 cm | 3 | 2,34 | 0,058 | 0,034 | 2,27 | 2,4 |
|  | 15 cm | 3 | 2,34 | 0,058 | 0,034 | 2,27 | 2,4 |
|  | Total | 12 | 2,34 | 0,052 | 0,015 | 2,32 | 2,38 |
| Vertical | No extension | 3 | 2,37 | 0,058 | 0,034 | 2,31 | 2,44 |
|  | 5 cm | 3 | 2,37 | 0,058 | 0,034 | 2,31 | 2,44 |
|  | 10 cm | 3 | 2,34 | 0,058 | 0,034 | 2,27 | 2,4 |
|  | 15 cm | 3 | 2,34 | 0,058 | 0,034 | 2,27 | 2,4 |
|  | Total | 12 | 2,35 | 0,052 | 0,015 | 2,33 | 2,38 |
| Mean | No extension | 3 | 2,37 | 0,052 | 0,021 | 2,33 | 2,41 |
|  | 5 cm | 3 | 2,35 | 0,055 | 0,022 | 2,31 | 2,39 |
|  | 10 cm | 3 | 2,34 | 0,052 | 0,021 | 2,29 | 2,37 |
|  | 15 cm | 3 | 2,34 | 0,052 | 0,021 | 2,29 | 2,37 |
|  | Total | 12 | 2,35 | 0,051 | 0,01 | 2,33 | 2,37 |

**Table 3** Entry Hole Variance Analysis at Adjacent Distances Based on Extension Lengths.

| **ANOVA** | | | | | |
| --- | --- | --- | --- | --- | --- |
| Measurement on Target | |  |  |  |  |
|  | Sums of Squares  **(SS)** | Degrees of Freedom **(df)** | Means Squares **(MS)** | F Value | Significance |
| Between Groups | ,005 | 3 | ,002 | ,556 | ,650 |
| In Groups | ,055 | 20 | ,003 |  |  |
| Total | ,060 | 23 |  |  |  |

**Table 4** Entry Hole Diameters Multiple Comparisons at Adjacent Distances based on Extension Lengths.

| **Multiple Comparison** | | | | | | |
| --- | --- | --- | --- | --- | --- | --- |
| Measurement on Target  Tukey HSD | | |  |  |  |  |
| (I) Extension  Lenght | (J) Extension  Lenght | Mean Difference  (I-J) | Standart  Error | Significance | 95% Confidence Interval | |
|  |  |  |  |  | Lower Limit | Upper Limit |
| No extension | 5 cm | ,01667 | ,03028 | ,945 | -,0681 | ,1014 |
|  | 10 cm | ,03333 | ,03028 | ,693 | -,0514 | ,1181 |
|  | 15 cm | ,03333 | ,03028 | ,693 | -,0514 | ,1181 |
| 5 cm | No extension | -,01667 | ,03028 | ,945 | -,1014 | ,0681 |
|  | 10 cm | ,01667 | ,03028 | ,945 | -,0681 | ,1014 |
|  | 15 cm | ,01667 | ,03028 | ,945 | -,0681 | ,1014 |
| 10 cm | No extension | -,03333 | ,03028 | ,693 | -,1181 | ,0514 |
|  | 5 cm | -,01667 | ,03028 | ,945 | -,1014 | ,0681 |
|  | 15 cm | ,00000 | ,03028 | 1,000 | -,0847 | ,0847 |
| 15 cm | No extension | -,03333 | ,03028 | ,693 | -,1181 | ,0514 |
|  | 5 cm | -,01667 | ,03028 | ,945 | -,1014 | ,0681 |
|  | 10 cm | ,00000 | ,03028 | 1,000 | -,0847 | ,0847 |

**Table 5** Soot Distribution on at adjacent distances based on Extension Lengths.

| Measurement  Type | Extension  Lenght | Number  Of  Shots | Arithmetic Mean  (cm) | Standard Deviation | Standard Error | Confidence Interval (%95)  Lower Limit | Confidence Interval (%95)  Upper Limit |
| --- | --- | --- | --- | --- | --- | --- | --- |
| Horizantal | No extension | 3 | 8,67 | 0,058 | 0,033 | 8,6 | 8,73 |
|  | 5 cm | 3 | 8,67 | 0,058 | 0,033 | 8,6 | 8,73 |
|  | 10 cm | 3 | 8,67 | 0,058 | 0,033 | 8,6 | 8,73 |
|  | 15 cm | 3 | 8,67 | 0,058 | 0,033 | 8,6 | 8,73 |
|  | Total | 12 | 8,67 | 0,05 | 0,014 | 8,64 | 8,7 |
| Vertical | No extension | 3 | 8,74 | 0,21 | 0,12 | 8,5 | 8,97 |
|  | 5 cm | 3 | 8,64 | 0,15 | 0,09 | 8,46 | 8,81 |
|  | 10 cm | 3 | 8,67 | 0,15 | 0,09 | 8,5 | 8,84 |
|  | 15 cm | 3 | 8,64 | 0,15 | 0,09 | 8,46 | 8,81 |
|  | Total | 12 | 8,67 | 0,15 | 0,09 | 8,58 | 8,75 |
| Mean | No extension | 3 | 8,7 | 0,14 | 0,057 | 8,59 | 8,81 |
|  | 5 cm | 3 | 8,65 | 0,1 | 0,041 | 8,57 | 8,73 |
|  | 10 cm | 3 | 8,67 | 0,1 | 0,041 | 8,58 | 8,75 |
|  | 15 cm | 3 | 8,65 | 0,1 | 0,041 | 8,57 | 8,73 |
|  | Total | 12 | 8,67 | 0,11 | 0,022 | 8,62 | 8,71 |

**Table 6** Soot Diameter Variance Analysis at Adjacent Distances Based on Extension Lengths.

| **ANOVA** | | | | | |
| --- | --- | --- | --- | --- | --- |
| Measurement on Target | |  |  |  |  |
|  | Sums of Squares  **(SS)** | Degrees of Freedom **(df)** | Means Squares **(MS)** | F Value | Significance |
| Between Groups | ,010 | 3 | ,003 | ,253 | ,858 |
| In Groups | ,263 | 20 | ,013 |  |  |
| Total | ,273 | 23 |  |  |  |

**Table 7** Soot Diameter Multiple Comparison Analysis at Adjacent Distances Based on Extension Lengths.

| **Multiple Comparison** | | | | | | |
| --- | --- | --- | --- | --- | --- | --- |
| Measurement on Target  Tukey HSD | | |  |  |  |  |
| (I) Extension  Length | (J) Extension  Length | Mean Difference  (I-J) | Standart  Error | Significance | 95% Confidence Interval | |
|  |  |  |  |  | Lower Limit | Upper Limit |
| No extension | 5 cm | ,05000 | ,06625 | ,874 | -,1354 | ,2354 |
|  | 10 cm | ,03333 | ,06625 | ,957 | -,1521 | ,2188 |
|  | 15 cm | ,05000 | ,06625 | ,874 | -,1354 | ,2354 |
| 5 cm | No extension | -,05000 | ,06625 | ,874 | -,2354 | ,1354 |
|  | 10 cm | -,01667 | ,06625 | ,994 | -,2021 | ,1688 |
|  | 15 cm | ,00000 | ,06625 | 1,000 | -,1854 | ,1854 |
| 10 cm | No extension | -,03333 | ,06625 | ,957 | -,2188 | ,1521 |
|  | 5 cm | ,01667 | ,06625 | ,994 | -,1688 | ,2021 |
|  | 15 cm | ,01667 | ,06625 | ,994 | -,1688 | ,2021 |
| 15 cm | No extension | -,05000 | ,06625 | ,874 | -,2354 | ,1354 |
|  | 5 cm | ,00000 | ,06625 | 1,000 | -,1854 | ,1854 |
|  | 10 cm | -,01667 | ,06625 | ,994 | -,2021 | ,1688 |

**Table 8** Entry hole diameters based on extension length at 5cm range.

| Measurement  Type | Extension  Lenght | Number  Of  Shots | Arithmetic Mean  (cm) | Standard Deviation | Standard Error | Confidence Interval (%95)  Lower Limit | Confidence Interval (%95)  Upper Limit |
| --- | --- | --- | --- | --- | --- | --- | --- |
| Horizantal | Şoksuz | 3 | 2,93 | 0,058 | 0,033 | 2,87 | 3 |
|  | 5 cm | 3 | 2,93 | 0,058 | 0,033 | 2,87 | 3 |
|  | 10 cm | 3 | 2,93 | 0,058 | 0,033 | 2,87 | 3 |
|  | 15 cm | 3 | 2,93 | 0,058 | 0,033 | 2,87 | 3 |
|  | Toplam | 12 | 2,93 | 0,049 | 0,014 | 2,91 | 2,96 |
| Vertical | Şoksuz | 3 | 3 | 0,1 | 0,058 | 2,89 | 3,11 |
|  | 5 cm | 3 | 3 | 0,1 | 0,058 | 2,89 | 3,11 |
|  | 10 cm | 3 | 2,93 | 0,115 | 0,067 | 2,8 | 3,06 |
|  | 15 cm | 3 | 2,9 | 0,1 | 0,058 | 2,79 | 3,01 |
|  | Toplam | 12 | 2,96 | 0,1 | 0,029 | 2,9 | 3,01 |
| Mean | Şoksuz | 3 | 2,97 | 0,082 | 0,033 | 2,9 | 3,03 |
|  | 5 cm | 3 | 2,97 | 0,082 | 0,033 | 2,9 | 3,03 |
|  | 10 cm | 3 | 2,93 | 0,082 | 0,033 | 2,87 | 3 |
|  | 15 cm | 3 | 2,92 | 0,075 | 0,031 | 2,86 | 2,98 |
|  | Toplam | 12 | 2,95 | 0,078 | 0,016 | 2,91 | 2,98 |

**Table 9** Entry Hole Variance Analysis at 5cm Distance based on Extension Lengths.

| **ANOVA** | | | | | |
| --- | --- | --- | --- | --- | --- |
| Measurement on Target | |  |  |  |  |
|  | Sums of Squares  **(SS)** | Degrees of Freedom **(df)** | Means Squares **(MS)** | F Value | Significance |
| Between Groups | ,011 | 3 | ,004 | ,584 | ,632 |
| In Groups | ,128 | 20 | ,006 |  |  |
| Total | ,140 | 23 |  |  |  |

**Table 10** Entry Hole Diameters Multiple Comparisons at 5cm Distance based on Extension Lengths.

| **Multiple Comparison** | | | | | | |
| --- | --- | --- | --- | --- | --- | --- |
| Measurement on Target  Tukey HSD | | |  |  |  |  |
| (I) Extension  Length | (J) Extension  Length | Mean Difference  (I-J) | Standard  Error | Significance | 95% Confidence Interval | |
|  |  |  |  |  | Lower Limit | Upper Limit |
| No extension | 5 cm | ,00000 | ,04625 | 1,000 | -,1294 | ,1294 |
|  | 10 cm | ,03333 | ,04625 | ,888 | -,0961 | ,1628 |
|  | 15 cm | ,05000 | ,04625 | ,705 | -,0794 | ,1794 |
| 5 cm | No extension | ,00000 | ,04625 | 1,000 | -,1294 | ,1294 |
|  | 10 cm | ,03333 | ,04625 | ,888 | -,0961 | ,1628 |
|  | 15 cm | ,05000 | ,04625 | ,705 | -,0794 | ,1794 |
| 10 cm | No extension | -,03333 | ,04625 | ,888 | -,1628 | ,0961 |
|  | 5 cm | -,03333 | ,04625 | ,888 | -,1628 | ,0961 |
|  | 15 cm | ,01667 | ,04625 | ,984 | -,1128 | ,1461 |
| 15 cm | No extension | -,05000 | ,04625 | ,705 | -,1794 | ,0794 |
|  | 5 cm | -,05000 | ,04625 | ,705 | -,1794 | ,0794 |
|  | 10 cm | -,01667 | ,04625 | ,984 | -,1461 | ,1128 |

**Table 11** Soot Distribution at 5cm distances based on Extension Lengths

| Measurement  Type | Extension  Length | Number  Of  Shots | Arithmetic Mean  (cm) | Standard Deviation | Standard Error | Confidence Interval (%95)  Lower Limit | Confidence Interval (%95)  Upper Limit |
| --- | --- | --- | --- | --- | --- | --- | --- |
| Horizantal | No extension | 3 | 12,57 | 0,058 | 0,033 | 12,5 | 12,63 |
|  | 5 cm | 3 | 12,53 | 0,058 | 0,033 | 12,47 | 12,6 |
|  | 10 cm | 3 | 12,53 | 0,058 | 0,033 | 12,47 | 12,6 |
|  | 15 cm | 3 | 12,53 | 0,058 | 0,033 | 12,47 | 12,6 |
|  | Total | 12 | 12,54 | 0,051 | 0,015 | 12,51 | 12,57 |
| Vertical | No extension | 3 | 12,53 | 0,416 | 0,24 | 12,06 | 13 |
|  | 5 cm | 3 | 12,5 | 0,2 | 0,115 | 12,27 | 12,73 |
|  | 10 cm | 3 | 12,9 | 0,265 | 0,153 | 12,6 | 13,2 |
|  | 15 cm | 3 | 12,43 | 0,252 | 0,145 | 12,15 | 12,72 |
|  | Total | 12 | 12,59 | 0,315 | 0,091 | 12,41 | 12,77 |
| Mean | No extension | 3 | 12,55 | 0,266 | 0,109 | 12,34 | 12,76 |
|  | 5 cm | 3 | 12,52 | 0,133 | 0,054 | 12,41 | 12,62 |
|  | 10 cm | 3 | 12,72 | 0,264 | 0,108 | 12,51 | 12,93 |
|  | 15 cm | 3 | 12,48 | 0,172 | 0,07 | 12,35 | 12,62 |
|  | Total | 12 | 12,57 | 0,222 | 0,045 | 12,48 | 12,66 |

**Table 12** Soot Diameter Variance Analysis at 5cm Distances Based on Extension Lengths.

| **ANOVA** | | | | | |
| --- | --- | --- | --- | --- | --- |
| Measurement on Target | |  |  |  |  |
|  | Sums of Squares  **(SS)** | Degrees of Freedom **(df)** | Means Squares **(MS)** | F Value | Significance |
| Between Groups | ,193 | 3 | ,064 | 1,371 | ,280 |
| In Groups | ,940 | 20 | ,047 |  |  |
| Total | 1,133 | 23 |  |  |  |

**Table 13** Soot Diameter Multiple Comparison Analysis at 5cm Distances Based on Extension Lengths.

| **Multiple Comparison** | | | | | | |
| --- | --- | --- | --- | --- | --- | --- |
| Measurement on Target  Tukey HSD | | |  |  |  |  |
| (I) Extension  Lenght | (J) Extension  Lenght | Mean Difference  (I-J) | Standart  Error | Significance | 95% Confidence Interval | |
|  |  |  |  |  | Lower Limit | Upper Limit |
| No extension | 5 cm | ,03333 | ,12517 | ,993 | -,3170 | ,3837 |
|  | 10 cm | -,16667 | ,12517 | ,555 | -,5170 | ,1837 |
|  | 15 cm | ,06667 | ,12517 | ,950 | -,2837 | ,4170 |
| 5 cm | No extension | -,03333 | ,12517 | ,993 | -,3837 | ,3170 |
|  | 10 cm | -,20000 | ,12517 | ,402 | -,5503 | ,1503 |
|  | 15 cm | ,03333 | ,12517 | ,993 | -,3170 | ,3837 |
| 10 cm | No extension | ,16667 | ,12517 | ,555 | -,1837 | ,5170 |
|  | 5 cm | ,20000 | ,12517 | ,402 | -,1503 | ,5503 |
|  | 15 cm | ,23333 | ,12517 | ,274 | -,1170 | ,5837 |
| 15 cm | No extension | -,06667 | ,12517 | ,950 | -,4170 | ,2837 |
|  | 5 cm | -,03333 | ,12517 | ,993 | -,3837 | ,3170 |
|  | 10 cm | -,23333 | ,12517 | ,274 | -,5837 | ,1170 |

**Table 14** Pellet Distribution at 5cm distances based on Extension Lengths

| Measurement  Type | Extension  Lenght | Number  Of  Shots | Arithmetic Mean  (cm) | Standard Deviation | Standard Error | Confidence Interval (%95)  Lower Limit | Confidence Interval (%95)  Upper Limit |
| --- | --- | --- | --- | --- | --- | --- | --- |
| Horizantal | No extension | 3 | 4,9 | 0,1 | 0,058 | 4,79 | 5,01 |
|  | 5 cm | 3 | 4,93 | 0,058 | 0,033 | 4,87 | 5 |
|  | 10 cm | 3 | 4,93 | 0,058 | 0,033 | 4,87 | 5 |
|  | 15 cm | 3 | 4,93 | 0,115 | 0,067 | 4,8 | 5,06 |
|  | Total | 12 | 4,93 | 0,075 | 0,022 | 4,88 | 4,97 |
| Vertical | No extension | 3 | 4,9 | 0,2 | 0,115 | 4,67 | 5,13 |
|  | 5 cm | 3 | 5 | 0,2 | 0,115 | 4,77 | 5,23 |
|  | 10 cm | 3 | 4,97 | 0,153 | 0,088 | 4,79 | 5,14 |
|  | 15 cm | 3 | 4,93 | 0,208 | 0,12 | 4,7 | 5,17 |
|  | Total | 12 | 4,95 | 0,168 | 0,048 | 4,86 | 5,04 |
| Mean | No extension | 3 | 4,9 | 0,141 | 0,058 | 4,79 | 5,01 |
|  | 5 cm | 3 | 4,97 | 0,137 | 0,056 | 4,86 | 5,08 |
|  | 10 cm | 3 | 4,95 | 0,105 | 0,043 | 4,87 | 5,03 |
|  | 15 cm | 3 | 4,93 | 0,151 | 0,061 | 4,81 | 5,05 |
|  | Total | 12 | 4,94 | 0,128 | 0,026 | 4,89 | 4,99 |

**Table 15** Pellet Distribution Variance Analysis at 5cm Distance based on Extension Lengths.

| **ANOVA** | | | | | |
| --- | --- | --- | --- | --- | --- |
| Measurement on Target | |  |  |  |  |
|  | Sums of Squares  **(SS)** | Degrees of Freedom **(df)** | Means Squares **(MS)** | F Value | Significance |
| Between Groups | ,015 | 3 | ,005 | ,269 | ,847 |
| In Groups | ,362 | 20 | ,018 |  |  |
| Total | ,376 | 23 |  |  |  |

| **Table 16** Pellet Distribution Multiple Comparison Analysis at 5cm Distance based on Extension Lengths  **Multiple Comparison** | | | | | | |
| --- | --- | --- | --- | --- | --- | --- |
| Measurement on Target  Tukey HSD | | |  |  |  |  |
| (I) Extension  Length | (J) Extension  Length | Mean Difference  (I-J) | Standart  Error | Significance | 95% Confidence Interval | |
|  |  |  |  |  | Lower Limit | Upper Limit |
| No extension | 5 cm | -,06667 | ,07764 | ,826 | -,2840 | ,1506 |
|  | 10 cm | -,05000 | ,07764 | ,916 | -,2673 | ,1673 |
|  | 15 cm | -,03333 | ,07764 | ,973 | -,2506 | ,1840 |
| 5 cm | No extension | ,06667 | ,07764 | ,826 | -,1506 | ,2840 |
|  | 10 cm | ,01667 | ,07764 | ,996 | -,2006 | ,2340 |
|  | 15 cm | ,03333 | ,07764 | ,973 | -,1840 | ,2506 |
| 10 cm | No extension | ,05000 | ,07764 | ,916 | -,1673 | ,2673 |
|  | 5 cm | -,01667 | ,07764 | ,996 | -,2340 | ,2006 |
|  | 15 cm | ,01667 | ,07764 | ,996 | -,2006 | ,2340 |
| 15 cm | No extension | ,03333 | ,07764 | ,973 | -,1840 | ,2506 |
|  | 5 cm | -,03333 | ,07764 | ,973 | -,2506 | ,1840 |
|  | 10 cm | -,01667 | ,07764 | ,996 | -,2340 | ,2006 |

**Table 17** Entry hole diameters based on extension length at 25cm range

| Measurement  Type | Extension  Length | Number  Of  Shots | Arithmetic Mean  (cm) | Standard Deviation | Standard Error | Confidence Interval (%95)  Lower Limit | Confidence Interval (%95)  Upper Limit |
| --- | --- | --- | --- | --- | --- | --- | --- |
| Horizantal | No extension | 3 | 1,97 | 0,058 | 0,033 | 1,9 | 2,03 |
|  | 5 cm | 3 | 2,03 | 0,058 | 0,033 | 1,97 | 2,1 |
|  | 10 cm | 3 | 1,97 | 0,058 | 0,033 | 1,9 | 2,03 |
|  | 15 cm | 3 | 2 | 0,1 | 0,058 | 1,89 | 2,11 |
|  | Total | 12 | 1,99 | 0,067 | 0,019 | 1,95 | 2,03 |
| Vertical | No Extension | 3 | 1,97 | 0,153 | 0,088 | 1,79 | 2,14 |
|  | 5 cm | 3 | 1,97 | 0,058 | 0,033 | 1,9 | 2,03 |
|  | 10 cm | 3 | 1,9 | 0,1 | 0,058 | 1,79 | 2,01 |
|  | 15 cm | 3 | 2 | 0,1 | 0,058 | 1,89 | 2,11 |
|  | Total | 12 | 1,96 | 0,1 | 0,029 | 1,9 | 2,01 |
| Mean | No extension | 3 | 1,97 | 0,103 | 0,042 | 1,88 | 2,05 |
|  | 5 cm | 3 | 2 | 0,063 | 0,026 | 1,95 | 2,05 |
|  | 10 cm | 3 | 1,93 | 0,082 | 0,033 | 1,87 | 2 |
|  | 15 cm | 3 | 2 | 0,089 | 0,037 | 1,93 | 2,07 |
|  | Total | 12 | 1,98 | 0,085 | 0,017 | 1,94 | 2,01 |

**Table 18** Entry Hole Variance Analysis at 2*5cm* distance based on extension Lengths.

| **ANOVA** | | | | | |
| --- | --- | --- | --- | --- | --- |
| Measurement on Target | |  |  |  |  |
|  | Sums of Squares  **(SS)** | Degrees of Freedom **(df)** | Means Squares **(MS)** | F Value | Significance |
| Between Groups | ,018 | 3 | ,006 | ,833 | ,491 |
| In Groups | ,147 | 20 | ,007 |  |  |
| Total | ,165 | 23 |  |  |  |

**Table 19** Entry Hole Diameters Multiple Comparisons at 25cm Distance based on Extension Lengths.

| **Multiple Comparison** | | | | | | |
| --- | --- | --- | --- | --- | --- | --- |
| Measurement on Target  Tukey HSD | | |  |  |  |  |
| (I) Extension  Length | (J) Extension  Length | Mean Difference  (I-J) | Standard  Error | Significance | 95% Confidence Interval | |
|  |  |  |  |  | Lower Limit | Upper Limit |
| No extension | 5 cm | -,03333 | ,04944 | ,906 | -,1717 | ,1050 |
|  | 10 cm | ,03333 | ,04944 | ,906 | -,1050 | ,1717 |
|  | 15 cm | -,03333 | ,04944 | ,906 | -,1717 | ,1050 |
| 5 cm | No extension | ,03333 | ,04944 | ,906 | -,1050 | ,1717 |
|  | 10 cm | ,06667 | ,04944 | ,544 | -,0717 | ,2050 |
|  | 15 cm | ,00000 | ,04944 | 1,000 | -,1384 | ,1384 |
| 10 cm | No extension | -,03333 | ,04944 | ,906 | -,1717 | ,1050 |
|  | 5 cm | -,06667 | ,04944 | ,544 | -,2050 | ,0717 |
|  | 15 cm | -,06667 | ,04944 | ,544 | -,2050 | ,0717 |
| 15 cm | No extension | ,03333 | ,04944 | ,906 | -,1050 | ,1717 |
|  | 5 cm | ,00000 | ,04944 | 1,000 | -,1384 | ,1384 |
|  | 10 cm | ,06667 | ,04944 | ,544 | -,0717 | ,2050 |

**Table 20.** Soot Distribution at 25cm distances based on Extension Lengths

| Measurement  Type | Extension  Length | Number  Of  Shots | Arithmetic Mean  (cm) | Standard Deviation | Standard Error | Confidence Interval (%95)  Lower Limit | Confidence Interval (%95)  Upper Limit |
| --- | --- | --- | --- | --- | --- | --- | --- |
| Horizantal | No extension | 3 | 16,07 | 0,153 | 0,088 | 15,89 | 16,24 |
|  | 5 cm | 3 | 16 | 0,2 | 0,115 | 15,77 | 16,23 |
|  | 10 cm | 3 | 16,1 | 0,1 | 0,058 | 15,99 | 16,21 |
|  | 15 cm | 3 | 16 | 0,265 | 0,153 | 15,7 | 16,3 |
|  | Total | 12 | 16,04 | 0,168 | 0,048 | 15,95 | 16,14 |
| Vertical | No extension | 3 | 15,97 | 0,252 | 0,145 | 15,68 | 16,25 |
|  | 5 cm | 3 | 15,8 | 0,2 | 0,115 | 15,57 | 16,03 |
|  | 10 cm | 3 | 16,13 | 0,379 | 0,219 | 15,7 | 16,56 |
|  | 15 cm | 3 | 16 | 0,1 | 0,058 | 15,89 | 16,11 |
|  | Total | 12 | 15,98 | 0,249 | 0,072 | 15,83 | 16,12 |
| Mean | No extension | 3 | 16,02 | 0,194 | 0,079 | 15,86 | 16,17 |
|  | 5 cm | 3 | 15,9 | 0,21 | 0,086 | 15,73 | 16,07 |
|  | 10 cm | 3 | 16,12 | 0,248 | 0,101 | 15,92 | 16,32 |
|  | 15 cm | 3 | 16 | 0,179 | 0,073 | 15,86 | 16,14 |
|  | Total | 12 | 16,01 | 0,21 | 0,043 | 15,92 | 16,09 |

**Table 21** Soot Diameter Variance Analysis at 25cm Distances Based on Extension Lengths.

| **ANOVA** | | | | | |
| --- | --- | --- | --- | --- | --- |
| Measurement on Target | |  |  |  |  |
|  | Sums of Squares  **(SS)** | Degrees of Freedom **(df)** | Means Squares **(MS)** | F Value | Significance |
| Between Groups | ,142 | 3 | ,047 | 1,077 | ,381 |
| In Groups | ,877 | 20 | ,044 |  |  |
| Total | 1,018 | 23 |  |  |  |

**Table 22** Soot Diameter Multiple Comparison Analysis at 25cm Distances Based on Extension Lengths.

| **Multiple Comparison** | | | | | | |
| --- | --- | --- | --- | --- | --- | --- |
| Measurement on Target  Tukey HSD | | |  |  |  |  |
| (I) Extension  Lenght | (J) Extension  Lenght | Mean Difference  (I-J) | Standart  Error | Significance | 95% Confidence Interval | |
|  |  |  |  |  | Lower Limit | Upper Limit |
| No extension | 5 cm | ,11667 | ,12088 | ,770 | -,2217 | ,4550 |
|  | 10 cm | -,10000 | ,12088 | ,841 | -,4383 | ,2383 |
|  | 15 cm | ,01667 | ,12088 | ,999 | -,3217 | ,3550 |
| 5 cm | No extension | -,11667 | ,12088 | ,770 | -,4550 | ,2217 |
|  | 10 cm | -,21667 | ,12088 | ,306 | -,5550 | ,1217 |
|  | 15 cm | -,10000 | ,12088 | ,841 | -,4383 | ,2383 |
| 10 cm | No extension | ,10000 | ,12088 | ,841 | -,2383 | ,4383 |
|  | 5 cm | ,21667 | ,12088 | ,306 | -,1217 | ,5550 |
|  | 15 cm | ,11667 | ,12088 | ,770 | -,2217 | ,4550 |
| 15 cm | No extension | -,01667 | ,12088 | ,999 | -,3550 | ,3217 |
|  | 5 cm | ,10000 | ,12088 | ,841 | -,2383 | ,4383 |
|  | 10 cm | -,11667 | ,12088 | ,770 | -,4550 | ,2217 |

**Table 23** Pellet Distribution at 25cm distances based on Extension Lengths.

| Measurement  Type | Extension  Length | Number  Of  Shots | Arithmetic Mean  (cm) | Standard Deviation | Standard Error | Confidence Interval (%95)  Lower Limit | Confidence Interval (%95)  Upper Limit |
| --- | --- | --- | --- | --- | --- | --- | --- |
| Horizantal | No extension | 3 | 13,37 | 0,321 | 0,186 | 13 | 13,73 |
|  | 5 cm | 3 | 13,63 | 0,208 | 0,12 | 13,4 | 13,87 |
|  | 10 cm | 3 | 13,23 | 0,252 | 0,145 | 12,95 | 13,52 |
|  | 15 cm | 3 | 13,27 | 0,208 | 0,12 | 13,03 | 13,5 |
|  | Total | 12 | 13,38 | 0,27 | 0,078 | 13,22 | 13,53 |
| Vertical | No extension | 3 | 13,3 | 0,173 | 0,1 | 13,1 | 13,5 |
|  | 5 cm | 3 | 13,47 | 0,451 | 0,26 | 12,96 | 13,98 |
|  | 10 cm | 3 | 13,17 | 0,208 | 0,12 | 12,93 | 13,4 |
|  | 15 cm | 3 | 13,27 | 0,306 | 0,176 | 12,92 | 13,61 |
|  | Total | 12 | 13,3 | 0,283 | 0,082 | 13,14 | 13,46 |
| Mean | No extension | 3 | 13,33 | 0,234 | 0,095 | 13,15 | 13,52 |
|  | 5 cm | 3 | 13,55 | 0,327 | 0,134 | 13,29 | 13,81 |
|  | 10 cm | 3 | 13,2 | 0,21 | 0,086 | 13,03 | 13,37 |
|  | 15 cm | 3 | 13,27 | 0,234 | 0,095 | 13,08 | 13,45 |
|  | Total | 12 | 13,34 | 0,273 | 0,056 | 13,23 | 13,45 |

**Table 24** Pellet Distribution Variance Analysis at 25cm Distance based on Extension Lengths.

| **ANOVA** | | | | | |
| --- | --- | --- | --- | --- | --- |
| Measurement on Target | |  |  |  |  |
|  | Sums of Squares  **(SS)** | Degrees of Freedom **(df)** | Means Squares **(MS)** | F Value | Significance |
| Between Groups | ,415 | 3 | ,138 | 2,123 | ,129 |
| In Groups | 1,302 | 20 | ,065 |  |  |
| Total | 1,716 | 23 |  |  |  |

**Table 25** Pellet Distribution Multiple Comparison Analysis at 25cm Distance based on Extension Lengths

| **Multiple Comparison** | | | | | | |
| --- | --- | --- | --- | --- | --- | --- |
| Measurement on Target  Tukey HSD | | |  |  |  |  |
| (I) Extension  Length | (J) Extension  Length | Mean Difference  (I-J) | Standart  Error | Significance | 95% Confidence Interval | |
|  |  |  |  |  | Lower Limit | Upper Limit |
| No extension | 5 cm | -,21667 | ,14729 | ,472 | -,6289 | ,1956 |
|  | 10 cm | ,13333 | ,14729 | ,802 | -,2789 | ,5456 |
|  | 15 cm | ,06667 | ,14729 | ,968 | -,3456 | ,4789 |
| 5 cm | No extension | ,21667 | ,14729 | ,472 | -,1956 | ,6289 |
|  | 10 cm | ,35000 | ,14729 | ,114 | -,0623 | ,7623 |
|  | 15 cm | ,28333 | ,14729 | ,250 | -,1289 | ,6956 |
| 10 cm | No extension | -,13333 | ,14729 | ,802 | -,5456 | ,2789 |
|  | 5 cm | -,35000 | ,14729 | ,114 | -,7623 | ,0623 |
|  | 15 cm | -,06667 | ,14729 | ,968 | -,4789 | ,3456 |
| 15 cm | No extension | -,06667 | ,14729 | ,968 | -,4789 | ,3456 |
|  | 5 cm | -,28333 | ,14729 | ,250 | -,6956 | ,1289 |
|  | 10 cm | ,06667 | ,14729 | ,968 | -,3456 | ,4789 |

**Table 26** Entry hole diameters based on extension length at 50cm range

| Measurement  Type | Extension  Length | Number  Of  Shots | Arithmetic Mean  (cm) | Standard Deviation | Standard Error | Confidence Interval (%95)  Lower Limit | Confidence Interval (%95)  Upper Limit |
| --- | --- | --- | --- | --- | --- | --- | --- |
| Horizantal | No extension | 3 | 2,03 | 0,058 | 0,033 | 1,97 | 2,1 |
|  | 5 cm | 3 | 2,07 | 0,058 | 0,033 | 2 | 2,13 |
|  | 10 cm | 3 | 2,03 | 0,058 | 0,033 | 1,97 | 2,1 |
|  | 15 cm | 3 | 2,03 | 0,058 | 0,033 | 1,97 | 2,1 |
|  | Total | 12 | 2,04 | 0,051 | 0,015 | 2,01 | 2,07 |
| Vertical | No extension | 3 | 2 | 0,1 | 0,058 | 1,89 | 2,11 |
|  | 5 cm | 3 | 2 | 0,173 | 0,1 | 1,8 | 2,2 |
|  | 10 cm | 3 | 2,03 | 0,153 | 0,088 | 1,86 | 2,21 |
|  | 15 cm | 3 | 2,03 | 0,115 | 0,067 | 1,9 | 2,16 |
|  | Total | 12 | 2,02 | 0,119 | 0,034 | 1,95 | 2,08 |
| Mean | No extension | 3 | 2,02 | 0,075 | 0,031 | 1,96 | 2,08 |
|  | 5 cm | 3 | 2,03 | 0,121 | 0,049 | 1,94 | 2,13 |
|  | 10 cm | 3 | 2,03 | 0,103 | 0,042 | 1,95 | 2,12 |
|  | 15 cm | 3 | 2,03 | 0,082 | 0,033 | 1,97 | 2,1 |
|  | Total | 12 | 2,03 | 0,091 | 0,019 | 1,99 | 2,07 |

**Table 27** Entry Hole Variance Analysis at 50cm Distance based on Extension Lengths.

| **ANOVA** | | | | | |
| --- | --- | --- | --- | --- | --- |
| Measurement on Target | |  |  |  |  |
|  | Sums of Squares  **(SS)** | Degrees of Freedom **(df)** | Means Squares **(MS)** | F Value | Significance |
| Between Groups | ,001 | 3 | ,000 | ,044 | ,987 |
| In Groups | ,188 | 20 | ,009 |  |  |
| Total | ,190 | 23 |  |  |  |

**Table 28** Entry Hole Diameters Multiple Comparisons at 50cm Distance based on Extension Lengths.

| **Multiple Comparison** | | | | | | |
| --- | --- | --- | --- | --- | --- | --- |
| Measurement on Target  Tukey HSD | | |  |  |  |  |
| (I) Extension  Length | (J) Extension  Length | Mean Difference  (I-J) | Standard  Error | Significance | 95% Confidence Interval | |
|  |  |  |  |  | Lower Limit | Upper Limit |
| No extension | 5 cm | -,01667 | ,05603 | ,991 | -,1735 | ,1401 |
|  | 10 cm | -,01667 | ,05603 | ,991 | -,1735 | ,1401 |
|  | 15 cm | -,01667 | ,05603 | ,991 | -,1735 | ,1401 |
| 5 cm | No extension | ,01667 | ,05603 | ,991 | -,1401 | ,1735 |
|  | 10 cm | ,00000 | ,05603 | 1,000 | -,1568 | ,1568 |
|  | 15 cm | ,00000 | ,05603 | 1,000 | -,1568 | ,1568 |
| 10 cm | No extension | ,01667 | ,05603 | ,991 | -,1401 | ,1735 |
|  | 5 cm | ,00000 | ,05603 | 1,000 | -,1568 | ,1568 |
|  | 15 cm | ,00000 | ,05603 | 1,000 | -,1568 | ,1568 |
| 15 cm | No extension | ,01667 | ,05603 | ,991 | -,1401 | ,1735 |
|  | 5 cm | ,00000 | ,05603 | 1,000 | -,1568 | ,1568 |
|  | 10 cm | ,00000 | ,05603 | 1,000 | -,1568 | ,1568 |

**Table 29** Soot Distribution at 50cm distances based on Extension Lengths.

| Measurement  Type | Extension  Length | Number  Of  Shots | Arithmetic Mean  (cm) | Standard Deviation | Standard Error | Confidence Interval (%95)  Lower Limit | Confidence Interval (%95)  Upper Limit |
| --- | --- | --- | --- | --- | --- | --- | --- |
| Horizantal | No extension | 3 | 19,97 | 0,153 | 0,088 | 19,79 | 20,14 |
|  | 5 cm | 3 | 20 | 0,1 | 0,058 | 19,89 | 20,11 |
|  | 10 cm | 3 | 20,03 | 0,208 | 0,12 | 19,8 | 20,27 |
|  | 15 cm | 3 | 20 | 0,1 | 0,058 | 19,89 | 20,11 |
|  | Total | 12 | 20 | 0,128 | 0,037 | 19,93 | 20,07 |
| Vertical | No extension | 3 | 19,93 | 0,208 | 0,12 | 19,7 | 20,17 |
|  | 5 cm | 3 | 19,93 | 0,351 | 0,203 | 19,54 | 20,33 |
|  | 10 cm | 3 | 20,13 | 0,231 | 0,133 | 19,87 | 20,39 |
|  | 15 cm | 3 | 19,93 | 0,231 | 0,133 | 19,67 | 20,19 |
|  | Total | 12 | 19,98 | 0,241 | 0,069 | 19,85 | 20,12 |
| Mean | No extension | 3 | 19,95 | 0,164 | 0,067 | 19,82 | 20,08 |
|  | 5 cm | 3 | 19,97 | 0,234 | 0,095 | 19,78 | 20,15 |
|  | 10 cm | 3 | 20,08 | 0,204 | 0,083 | 19,92 | 20,25 |
|  | 15 cm | 3 | 19,97 | 0,163 | 0,067 | 19,84 | 20,1 |
|  | Total | 12 | 19,99 | 0,189 | 0,039 | 19,92 | 20,07 |

**Table 30** Soot Diameter Variance Analysis at 50cm Distances Based on Extension Lengths.

| **ANOVA** | | | | | |
| --- | --- | --- | --- | --- | --- |
| Measurement on Target | |  |  |  |  |
|  | Sums of Squares  **(SS)** | Degrees of Freedom **(df)** | Means Squares **(MS)** | F Value | Significance |
| Between Groups | ,068 | 3 | ,023 | ,607 | ,618 |
| In Groups | ,750 | 20 | ,038 |  |  |
| Total | ,818 | 23 |  |  |  |

**Table 31** Soot Diameter Multiple Comparison Analysis at 50cm Distances Based on Extension Lengths.

| **Multiple Comparison** | | | | | | |
| --- | --- | --- | --- | --- | --- | --- |
| Measurement on Target  Tukey HSD | | |  |  |  |  |
| (I) Extension  Length | (J) Extension  Length | Mean Difference  (I-J) | Standart  Error | Significance | 95% Confidence Interval | |
|  |  |  |  |  | Lower Limit | Upper Limit |
| No extension | 5 cm | -,01667 | ,11180 | ,999 | -,3296 | ,2963 |
|  | 10 cm | -,13333 | ,11180 | ,638 | -,4463 | ,1796 |
|  | 15 cm | -,01667 | ,11180 | ,999 | -,3296 | ,2963 |
| 5 cm | No extension | ,01667 | ,11180 | ,999 | -,2963 | ,3296 |
|  | 10 cm | -,11667 | ,11180 | ,726 | -,4296 | ,1963 |
|  | 15 cm | ,00000 | ,11180 | 1,000 | -,3129 | ,3129 |
| 10 cm | No extension | ,13333 | ,11180 | ,638 | -,1796 | ,4463 |
|  | 5 cm | ,11667 | ,11180 | ,726 | -,1963 | ,4296 |
|  | 15 cm | ,11667 | ,11180 | ,726 | -,1963 | ,4296 |
| 15 cm | No extension | ,01667 | ,11180 | ,999 | -,2963 | ,3296 |
|  | 5 cm | ,00000 | ,11180 | 1,000 | -,3129 | ,3129 |
|  | 10 cm | -,11667 | ,11180 | ,726 | -,4296 | ,1963 |

**Table 32** Pellet Distribution at 50cm distances based on Extension Lengths.

| Measurement  Type | Extension  Length | Number  Of  Shots | Arithmetic Mean  (cm) | Standard Deviation | Standard Error | Confidence Interval (%95)  Lower Limit | Confidence Interval (%95)  Upper Limit |
| --- | --- | --- | --- | --- | --- | --- | --- |
| Horizantal | No extension | 3 | 17,73 | 0,208 | 0,12 | 17,5 | 17,97 |
|  | 5 cm | 3 | 17,6 | 0,1 | 0,058 | 17,49 | 17,71 |
|  | 10 cm | 3 | 17,63 | 0,153 | 0,088 | 17,46 | 17,81 |
|  | 15 cm | 3 | 17,73 | 0,208 | 0,12 | 17,5 | 17,97 |
|  | Total | 12 | 17,68 | 0,16 | 0,046 | 17,58 | 17,77 |
| Vertical | No extension | 3 | 17,83 | 0,153 | 0,088 | 17,66 | 18,01 |
|  | 5 cm | 3 | 17,77 | 0,321 | 0,186 | 17,4 | 18,13 |
|  | 10 cm | 3 | 17,8 | 0,1 | 0,058 | 17,69 | 17,91 |
|  | 15 cm | 3 | 17,67 | 0,306 | 0,176 | 17,32 | 18,01 |
|  | Total | 12 | 17,77 | 0,215 | 0,062 | 17,65 | 17,89 |
| Mean | No extension | 3 | 17,78 | 0,172 | 0,07 | 17,65 | 17,92 |
|  | 5 cm | 3 | 17,68 | 0,232 | 0,095 | 17,5 | 17,87 |
|  | 10 cm | 3 | 17,72 | 0,147 | 0,06 | 17,6 | 17,83 |
|  | 15 cm | 3 | 17,7 | 0,237 | 0,097 | 17,51 | 17,89 |
|  | Total | 12 | 17,72 | 0,191 | 0,039 | 17,64 | 17,8 |

**Table 33** Pellet Distribution Variance Analysis at 50cm Distance based on Extension Lengths.

| **ANOVA** | | | | | |
| --- | --- | --- | --- | --- | --- |
| Measurement on Target | |  |  |  |  |
|  | Sums of Squares  **(SS)** | Degrees of Freedom **(df)** | Means Squares **(MS)** | F Value | Significance |
| Between Groups | ,035 | 3 | ,012 | ,286 | ,835 |
| In Groups | ,805 | 20 | ,040 |  |  |
| Total | ,840 | 23 |  |  |  |

**Table 34** Pellet Distribution Multiple Comparison Analysis at 50cm Distance based on Extension Lengths

| **Multiple Comparison** | | | | | | |
| --- | --- | --- | --- | --- | --- | --- |
| Measurement on Target  Tukey HSD | | |  |  |  |  |
| (I) Extension  Length | (J) Extension  Length | Mean Difference  (I-J) | Standard  Error | Significance | 95% Confidence Interval | |
|  |  |  |  |  | Lower Limit | Upper Limit |
| No extension | 5 cm | ,10000 | ,11583 | ,823 | -,2242 | ,4242 |
|  | 10 cm | ,06667 | ,11583 | ,938 | -,2575 | ,3909 |
|  | 15 cm | ,08333 | ,11583 | ,888 | -,2409 | ,4075 |
| 5 cm | No extension | -,10000 | ,11583 | ,823 | -,4242 | ,2242 |
|  | 10 cm | -,03333 | ,11583 | ,991 | -,3575 | ,2909 |
|  | 15 cm | -,01667 | ,11583 | ,999 | -,3409 | ,3075 |
| 10 cm | No extension | -,06667 | ,11583 | ,938 | -,3909 | ,2575 |
|  | 5 cm | ,03333 | ,11583 | ,991 | -,2909 | ,3575 |
|  | 15 cm | ,01667 | ,11583 | ,999 | -,3075 | ,3409 |
| 15 cm | No extension | -,08333 | ,11583 | ,888 | -,4075 | ,2409 |
|  | 5 cm | ,01667 | ,11583 | ,999 | -,3075 | ,3409 |
|  | 10 cm | -,01667 | ,11583 | ,999 | -,3409 | ,3075 |

**Table 35** Entry hole diameters based on extension length at 75cm range.

| Measurement  Type | Extension  Length | Number  Of  Shots | Arithmetic Mean  (cm) | Standard Deviation | Standard Error | Confidence Interval (%95)  Lower Limit | Confidence Interval (%95)  Upper Limit |
| --- | --- | --- | --- | --- | --- | --- | --- |
| Horizantal | No extension | 3 | 2,97 | 0,058 | 0,03 | 2,9 | 3,03 |
|  | 5 cm | 3 | 2,97 | 0,058 | 0,03 | 2,9 | 3,03 |
|  | 10 cm | 3 | 3,03 | 0,058 | 0,03 | 2,97 | 3,1 |
|  | 15 cm | 3 | 3 | 0,1 | 0,06 | 2,89 | 3,11 |
|  | Total | 12 | 2,99 | 0,067 | 0,02 | 2,95 | 3,03 |
| Vertical | No extension | 3 | 3,03 | 0,115 | 0,07 | 2,9 | 3,16 |
|  | 5 cm | 3 | 3,03 | 0,058 | 0,03 | 2,97 | 3,1 |
|  | 10 cm | 3 | 3 | 0,1 | 0,06 | 2,89 | 3,11 |
|  | 15 cm | 3 | 3,07 | 0,058 | 0,03 | 3 | 3,13 |
|  | Total | 12 | 3,03 | 0,078 | 0,02 | 2,99 | 3,08 |
| Mean | No extension | 3 | 3 | 0,089 | 0,04 | 2,93 | 3,07 |
|  | 5 cm | 3 | 3 | 0,063 | 0,03 | 2,95 | 3,05 |
|  | 10 cm | 3 | 3,02 | 0,075 | 0,03 | 2,96 | 3,08 |
|  | 15 cm | 3 | 3,03 | 0,082 | 0,03 | 2,97 | 3,1 |
|  | Total | 12 | 3,01 | 0,074 | 0,02 | 2,98 | 3,04 |

**Table 36** Entry Hole Variance Analysis at 75cm Distance based on Extension Lengths.

| **ANOVA** | | | | | |
| --- | --- | --- | --- | --- | --- |
| Measurement on Target | |  |  |  |  |
|  | Sums of Squares  **(SS)** | Degrees of Freedom **(df)** | Means Squares **(MS)** | F Value | Significance |
| Between Groups | ,005 | 3 | ,002 | ,251 | ,860 |
| In Groups | ,122 | 20 | ,006 |  |  |
| Total | ,126 | 23 |  |  |  |

**Table 37** Entry Hole Diameters Multiple Comparisons at 75cm Distance based on Extension Lengths.

| **Multiple Comparison** | | | | | | |
| --- | --- | --- | --- | --- | --- | --- |
| Measurement on Target  Tukey HSD | | |  |  |  |  |
| (I) Extension  Lenght | (J) Extension  Lenght | Mean Difference  (I-J) | Standart  Error | Significance | 95% Confidence Interval | |
|  |  |  |  |  | Lower Limit | Upper Limit |
| No extension | 5 cm | ,00000 | ,04503 | 1,000 | -,1260 | ,1260 |
|  | 10 cm | -,01667 | ,04503 | ,982 | -,1427 | ,1094 |
|  | 15 cm | -,03333 | ,04503 | ,880 | -,1594 | ,0927 |
| 5 cm | No extension | ,00000 | ,04503 | 1,000 | -,1260 | ,1260 |
|  | 10 cm | -,01667 | ,04503 | ,982 | -,1427 | ,1094 |
|  | 15 cm | -,03333 | ,04503 | ,880 | -,1594 | ,0927 |
| 10 cm | No extension | ,01667 | ,04503 | ,982 | -,1094 | ,1427 |
|  | 5 cm | ,01667 | ,04503 | ,982 | -,1094 | ,1427 |
|  | 15 cm | -,01667 | ,04503 | ,982 | -,1427 | ,1094 |
| 15 cm | No extension | ,03333 | ,04503 | ,880 | -,0927 | ,1594 |
|  | 5 cm | ,03333 | ,04503 | ,880 | -,0927 | ,1594 |
|  | 10 cm | ,01667 | ,04503 | ,982 | -,1094 | ,1427 |

**Table 38** Pellet Distribution on at 75cm distances based on Extension Lengths

| Measurement  Type | Extension  Length | Number  Of  Shots | Arithmetic Mean  (cm) | Standard Deviation | Standard Error | Confidence Interval (%95)  Lower Limit | Confidence Interval (%95)  Upper Limit |
| --- | --- | --- | --- | --- | --- | --- | --- |
| Horizantal | No extension | 3 | 22,97 | 0,551 | 0,318 | 22,34 | 23,59 |
|  | 5 cm | 3 | 22,87 | 0,208 | 0,12 | 22,63 | 23,1 |
|  | 10 cm | 3 | 23,17 | 0,252 | 0,145 | 22,88 | 23,45 |
|  | 15 cm | 3 | 22,87 | 0,208 | 0,12 | 22,63 | 23,1 |
|  | Total | 12 | 22,97 | 0,314 | 0,091 | 22,79 | 23,14 |
| Vertical | No extension | 3 | 22,73 | 0,208 | 0,12 | 22,5 | 22,97 |
|  | 5 cm | 3 | 23,1 | 0,361 | 0,208 | 22,69 | 23,51 |
|  | 10 cm | 3 | 23,13 | 0,306 | 0,176 | 22,79 | 23,48 |
|  | 15 cm | 3 | 23,07 | 0,208 | 0,12 | 22,83 | 23,3 |
|  | Total | 12 | 23,01 | 0,291 | 0,084 | 22,84 | 23,17 |
| Mean | No extension | 3 | 22,85 | 0,394 | 0,161 | 22,53 | 23,17 |
|  | 5 cm | 3 | 22,98 | 0,293 | 0,119 | 22,75 | 23,22 |
|  | 10 cm | 3 | 23,15 | 0,251 | 0,102 | 22,95 | 23,35 |
|  | 15 cm | 3 | 22,97 | 0,216 | 0,088 | 22,79 | 23,14 |
|  | Total | 12 | 22,99 | 0,297 | 0,061 | 22,87 | 23,11 |

**Table 39** Pellet Distribution Diameter Variance Analysis at 75cm Distances Based on Extension Lengths.

| **ANOVA** | | | | | |
| --- | --- | --- | --- | --- | --- |
| Measurement on Target | |  |  |  |  |
|  | Sums of Squares  **(SS)** | Degrees of Freedom **(df)** | Means Squares **(MS)** | F Value | Significance |
| Between Groups | ,275 | 3 | ,092 | 1,045 | ,394 |
| In Groups | 1,752 | 20 | ,088 |  |  |
| Total | 2,026 | 23 |  |  |  |

**Table 40** Pellet Distribution Diameter Multiple Comparison Analysis at 75cm Distances Based on Extension Lengths.

| **Multiple Comparison** | | | | | | |
| --- | --- | --- | --- | --- | --- | --- |
| Measurement on Target  Tukey HSD | | |  |  |  |  |
| (I) Extension  Length | (J) Extension  Length | Mean Difference  (I-J) | Standard  Error | Significance | 95% Confidence Interval | |
|  |  |  |  |  | Lower Limit | Upper Limit |
| No extension | 5 cm | -,13333 | ,17086 | ,862 | -,6116 | ,3449 |
|  | 10 cm | -,30000 | ,17086 | ,323 | -,7782 | ,1782 |
|  | 15 cm | -,11667 | ,17086 | ,902 | -,5949 | ,3616 |
| 5 cm | No extension | ,13333 | ,17086 | ,862 | -,3449 | ,6116 |
|  | 10 cm | -,16667 | ,17086 | ,765 | -,6449 | ,3116 |
|  | 15 cm | ,01667 | ,17086 | 1,000 | -,4616 | ,4949 |
| 10 cm | No extension | ,30000 | ,17086 | ,323 | -,1782 | ,7782 |
|  | 5 cm | ,16667 | ,17086 | ,765 | -,3116 | ,6449 |
|  | 15 cm | ,18333 | ,17086 | ,709 | -,2949 | ,6616 |
| 15 cm | No extension | ,11667 | ,17086 | ,902 | -,3616 | ,5949 |
|  | 5 cm | -,01667 | ,17086 | 1,000 | -,4949 | ,4616 |
|  | 10 cm | -,18333 | ,17086 | ,709 | -,6616 | ,2949 |

**Table 41** Entry hole diameters based on extension length at 100cm range

| Measurement  Type | Extension  Length | Number  Of  Shots | Arithmetic Mean  (cm) | Standard Deviation | Standard Error | Confidence Interval (%95)  Lower Limit | Confidence Interval (%95)  Upper Limit |
| --- | --- | --- | --- | --- | --- | --- | --- |
| Horizantal | No extension | 3 | 3,13 | 0,058 | 0,033 | 3,07 | 3,2 |
|  | 5 cm | 3 | 3,17 | 0,058 | 0,033 | 3,1 | 3,23 |
|  | 10 cm | 3 | 3,13 | 0,058 | 0,033 | 3,07 | 3,2 |
|  | 15 cm | 3 | 3,13 | 0,058 | 0,033 | 3,07 | 3,2 |
|  | Total | 12 | 3,14 | 0,051 | 0,015 | 3,11 | 3,17 |
| Vertical | No extension | 3 | 3,1 | 0,1 | 0,058 | 2,99 | 3,21 |
|  | 5 cm | 3 | 3,17 | 0,058 | 0,033 | 3,1 | 3,23 |
|  | 10 cm | 3 | 3,13 | 0,115 | 0,067 | 3 | 3,26 |
|  | 15 cm | 3 | 3,2 | 0,1 | 0,058 | 3,09 | 3,31 |
|  | Total | 12 | 3,15 | 0,09 | 0,026 | 3,1 | 3,2 |
| Mean | No extension | 3 | 3,12 | 0,075 | 0,031 | 3,06 | 3,18 |
|  | 5 cm | 3 | 3,17 | 0,052 | 0,021 | 3,13 | 3,21 |
|  | 10 cm | 3 | 3,13 | 0,082 | 0,033 | 3,07 | 3,2 |
|  | 15 cm | 3 | 3,17 | 0,082 | 0,033 | 3,1 | 3,23 |
|  | Total | 12 | 3,15 | 0,072 | 0,015 | 3,12 | 3,17 |

**Table 42** Entry Hole Variance Analysis at 100cm Distance based on Extension Length*s*.

| **ANOVA** | | | | | |
| --- | --- | --- | --- | --- | --- |
| Measurement on Target | |  |  |  |  |
|  | Sums of Squares  **(SS)** | Degrees of Freedom **(df)** | Means Squares **(MS)** | F Value | Significance |
| Between Groups | ,011 | 3 | ,004 | ,692 | ,567 |
| In Groups | ,108 | 20 | ,005 |  |  |
| Total | ,120 | 23 |  |  |  |

**Table 43** Entry Hole Diameters Multiple Comparisons at 100cm Distance based on Extension Lengths.

| **Multiple Comparison** | | | | | | |
| --- | --- | --- | --- | --- | --- | --- |
| Measurement on Target  Tukey HSD | | |  |  |  |  |
| (I) Extension  Lenght | (J) Extension  Lenght | Mean Difference  (I-J) | Standart  Error | Significance | 95% Confidence Interval | |
|  |  |  |  |  | Lower Limit | Upper Limit |
| No extension | 5 cm | -,05000 | ,04249 | ,648 | -,1689 | ,0689 |
|  | 10 cm | -,01667 | ,04249 | ,979 | -,1356 | ,1023 |
|  | 15 cm | -,05000 | ,04249 | ,648 | -,1689 | ,0689 |
| 5 cm | No extension | ,05000 | ,04249 | ,648 | -,0689 | ,1689 |
|  | 10 cm | ,03333 | ,04249 | ,861 | -,0856 | ,1523 |
|  | 15 cm | ,00000 | ,04249 | 1,000 | -,1189 | ,1189 |
| 10 cm | No extension | ,01667 | ,04249 | ,979 | -,1023 | ,1356 |
|  | 5 cm | -,03333 | ,04249 | ,861 | -,1523 | ,0856 |
|  | 15 cm | -,03333 | ,04249 | ,861 | -,1523 | ,0856 |
| 15 cm | No extension | ,05000 | ,04249 | ,648 | -,0689 | ,1689 |
|  | 5 cm | ,00000 | ,04249 | 1,000 | -,1189 | ,1189 |
|  | 10 cm | ,03333 | ,04249 | ,861 | -,0856 | ,1523 |

**Table 44** Pellet Distribution on at 100cm distances based on Extension Lengths

| Measurement  Type | Extension  Lenght | Number  Of  Shots | Arithmetic Mean  (cm) | Standard Deviation | Standard Error | Confidence Interval (%95)  Lower Limit | Confidence Interval (%95)  Upper Limit |
| --- | --- | --- | --- | --- | --- | --- | --- |
| Horizantal | No extension | 3 | 27,03 | 0,252 | 0,145 | 26,75 | 27,32 |
|  | 5 cm | 3 | 27,07 | 0,153 | 0,088 | 26,89 | 27,24 |
|  | 10 cm | 3 | 27,1 | 0,3 | 0,173 | 26,76 | 27,44 |
|  | 15 cm | 3 | 27,1 | 0,265 | 0,153 | 26,8 | 27,4 |
|  | Total | 12 | 27,08 | 0,214 | 0,062 | 26,95 | 27,2 |
| Vertical | No extension | 3 | 27,13 | 0,252 | 0,145 | 26,85 | 27,42 |
|  | 5 cm | 3 | 26,87 | 0,153 | 0,088 | 26,69 | 27,04 |
|  | 10 cm | 3 | 26,9 | 0,361 | 0,208 | 26,49 | 27,31 |
|  | 15 cm | 3 | 27,03 | 0,252 | 0,145 | 26,75 | 27,32 |
|  | Total | 12 | 26,98 | 0,252 | 0,073 | 26,84 | 27,13 |
| Mean | No extension | 3 | 27,08 | 0,232 | 0,095 | 26,9 | 27,27 |
|  | 5 cm | 3 | 26,97 | 0,175 | 0,071 | 26,83 | 27,11 |
|  | 10 cm | 3 | 27 | 0,316 | 0,129 | 26,75 | 27,25 |
|  | 15 cm | 3 | 27,07 | 0,234 | 0,095 | 26,88 | 27,25 |
|  | Total | 12 | 27,03 | 0,233 | 0,048 | 26,94 | 27,12 |

**Table 45** Pellet Distribution Diameter Variance Analysis at 100cm Distances Based on Extension Lengths.

| **ANOVA** | | | | | |
| --- | --- | --- | --- | --- | --- |
| Measurement on Target | |  |  |  |  |
|  | Sums of Squares  **(SS)** | Degrees of Freedom **(df)** | Means Squares **(MS)** | F Value | Significance |
| Between Groups | ,055 | 3 | ,018 | ,305 | ,822 |
| In Groups | 1,195 | 20 | ,060 |  |  |
| Total | 1,250 | 23 |  |  |  |

**Table 46** Pellet Distribution Diameter Multiple Comparison Analysis at 100cm Distances Based on Extension Lengths.

| **Multiple Comparison** | | | | | | |
| --- | --- | --- | --- | --- | --- | --- |
| Measurement on Target  Tukey HSD | | |  |  |  |  |
| (I) Extension  Lenght | (J) Extension  Lenght | Mean Difference  (I-J) | Standart  Error | Significance | 95% Confidence Interval | |
|  |  |  |  |  | Lower Limit | Upper Limit |
| No extension | 5 cm | ,11667 | ,14113 | ,841 | -,2783 | ,5117 |
|  | 10 cm | ,08333 | ,14113 | ,934 | -,3117 | ,4783 |
|  | 15 cm | ,01667 | ,14113 | ,999 | -,3783 | ,4117 |
| 5 cm | No extension | -,11667 | ,14113 | ,841 | -,5117 | ,2783 |
|  | 10 cm | -,03333 | ,14113 | ,995 | -,4283 | ,3617 |
|  | 15 cm | -,10000 | ,14113 | ,893 | -,4950 | ,2950 |
| 10 cm | No extension | -,08333 | ,14113 | ,934 | -,4783 | ,3117 |
|  | 5 cm | ,03333 | ,14113 | ,995 | -,3617 | ,4283 |
|  | 15 cm | -,06667 | ,14113 | ,964 | -,4617 | ,3283 |
| 15 cm | No extension | -,01667 | ,14113 | ,999 | -,4117 | ,3783 |
|  | 5 cm | ,10000 | ,14113 | ,893 | -,2950 | ,4950 |
|  | 10 cm | ,06667 | ,14113 | ,964 | -,3283 | ,4617 |

**Table 47** Entry hole diameters based on extension length at 200cm range

| Measurement  Type | Extension  Lenght | Number  Of  Shots | Arithmetic Mean  (cm) | Standard Deviation | Standard Error | Confidence Interval (%95)  Lower Limit | Confidence Interval (%95)  Upper Limit |
| --- | --- | --- | --- | --- | --- | --- | --- |
| Horizantal | No extension | 3 | 4,03 | 0,058 | 0,03 | 3,97 | 4,1 |
|  | 5 cm | 3 | 3,9 | 0,1 | 0,06 | 3,79 | 4,01 |
|  | 10 cm | 3 | 3,33 | 0,153 | 0,09 | 3,16 | 3,51 |
|  | 15 cm | 3 | 2,9 | 0,1 | 0,06 | 2,79 | 3,01 |
|  | Total | 12 | 3,54 | 0,483 | 0,14 | 3,27 | 3,82 |
| Vertical | No extension | 3 | 4 | 0,1 | 0,06 | 3,89 | 4,11 |
|  | 5 cm | 3 | 3,93 | 0,058 | 0,03 | 3,87 | 4 |
|  | 10 cm | 3 | 3,33 | 0,231 | 0,13 | 3,07 | 3,59 |
|  | 15 cm | 3 | 2,9 | 0,2 | 0,12 | 2,67 | 3,13 |
|  | Total | 12 | 3,54 | 0,493 | 0,14 | 3,26 | 3,82 |
| Mean | No extension | 3 | 4,02 | 0,075 | 0,03 | 3,96 | 4,08 |
|  | 5 cm | 3 | 3,92 | 0,075 | 0,03 | 3,86 | 3,98 |
|  | 10 cm | 3 | 3,33 | 0,175 | 0,07 | 3,19 | 3,47 |
|  | 15 cm | 3 | 2,9 | 0,141 | 0,06 | 2,79 | 3,01 |
|  | Total | 12 | 3,54 | 0,477 | 0,1 | 3,35 | 3,73 |

**Table 48** Entry Hole Variance Analysis at 200cm Distance based on Extension Lengths.

| **ANOVA** | | | | | |
| --- | --- | --- | --- | --- | --- |
| Measurement on Target | |  |  |  |  |
|  | Sums of Squares  **(SS)** | Degrees of Freedom **(df)** | Means Squares **(MS)** | F Value | Significance |
| Between Groups | 4,928 | 3 | 1,643 | 105,986 | ,000 |
| In Groups | ,310 | 20 | ,016 |  |  |
| Total | 5,238 | 23 |  |  |  |

**Table 49** Entry Hole Diameters Multiple Comparisons at 200cm Distance based on Extension Lengths.

| **Multiple Comparison** | | | | | | |
| --- | --- | --- | --- | --- | --- | --- |
| Measurement on Target  Tukey HSD | | |  |  |  |  |
| (I) Extension  Lenght | (J) Extension  Lenght | Mean Difference  (I-J) | Standart  Error | Significance | 95% Confidence Interval | |
|  |  |  |  |  | Lower Limit | Upper Limit |
| No extension | 5 cm | ,10000 | ,07188 | ,519 | -,1012 | ,3012 |
|  | 10 cm | ,68333* | ,07188 | ,000 | ,4821 | ,8845 |
|  | 15 cm | 1,11667* | ,07188 | ,000 | ,9155 | 1,3179 |
| 5 cm | No extension | -,10000 | ,07188 | ,519 | -,3012 | ,1012 |
|  | 10 cm | ,58333* | ,07188 | ,000 | ,3821 | ,7845 |
|  | 15 cm | 1,01667* | ,07188 | ,000 | ,8155 | 1,2179 |
| 10 cm | No extension | -,68333* | ,07188 | ,000 | -,8845 | -,4821 |
|  | 5 cm | -,58333* | ,07188 | ,000 | -,7845 | -,3821 |
|  | 15 cm | ,43333* | ,07188 | ,000 | ,2321 | ,6345 |
| 15 cm | No extension | -1,11667* | ,07188 | ,000 | -1,3179 | -,9155 |
|  | 5 cm | -1,01667* | ,07188 | ,000 | -1,2179 | -,8155 |
|  | 10 cm | -,43333* | ,07188 | ,000 | -,6345 | -,2321 |

**Table 50**  Pellet Distribution at 200cm distances based on Extension Lengths

| Measurement  Type | Extension  Lenght | Number  Of  Shots | Arithmetic Mean  (cm) | Standard Deviation | Standard Error | Confidence Interval (%95)  Lower Limit | Confidence Interval (%95)  Upper Limit |
| --- | --- | --- | --- | --- | --- | --- | --- |
| Horizantal | No extension | 3 | 8,4 | 0,1 | 0,058 | 8,29 | 8,51 |
|  | 5 cm | 3 | 8,1 | 0,1 | 0,058 | 7,99 | 8,21 |
|  | 10 cm | 3 | 7,8 | 0,1 | 0,058 | 7,69 | 7,91 |
|  | 15 cm | 3 | 7,5 | 0,1 | 0,058 | 7,39 | 7,61 |
|  | Total | 12 | 7,95 | 0,361 | 0,104 | 7,75 | 8,15 |
| Vertical | No extension | 3 | 8,47 | 0,153 | 0,088 | 8,29 | 8,64 |
|  | 5 cm | 3 | 7,97 | 0,058 | 0,033 | 7,9 | 8,03 |
|  | 10 cm | 3 | 7,8 | 0,1 | 0,058 | 7,69 | 7,91 |
|  | 15 cm | 3 | 7,43 | 0,153 | 0,088 | 7,26 | 7,61 |
|  | Total | 12 | 7,92 | 0,402 | 0,116 | 7,69 | 8,14 |
| Mean | No extension | 3 | 8,43 | 0,121 | 0,049 | 8,34 | 8,53 |
|  | 5 cm | 3 | 8,03 | 0,103 | 0,042 | 7,95 | 8,12 |
|  | 10 cm | 3 | 7,8 | 0,089 | 0,037 | 7,73 | 7,87 |
|  | 15 cm | 3 | 7,47 | 0,121 | 0,049 | 7,37 | 7,56 |
|  | Total | 12 | 7,93 | 0,374 | 0,076 | 7,78 | 8,08 |

**Table 51** Pellet Distribution Diameter Variance Analysis at 200cm Distances Based on Extension Lengths.

| **ANOVA** | | | | | |
| --- | --- | --- | --- | --- | --- |
| Measurement on Target | |  |  |  |  |
|  | Sums of Squares  **(SS)** | Degrees of Freedom **(df)** | Means Squares **(MS)** | F Value | Significance |
| Between Groups | 2,973 | 3 | ,991 | 82,593 | ,000 |
| In Groups | ,240 | 20 | ,012 |  |  |
| Total | 3,213 | 23 |  |  |  |

**Table 52** Pellet Distribution Diameter Multiple Comparison Analysis at 200cm Distances Based on Extension Lengths.

| **Multiple Comparison** | | | | | | |
| --- | --- | --- | --- | --- | --- | --- |
| Measurement on Target  Tukey HSD | | |  |  |  |  |
| (I) Extension  Lenght | (J) Extension  Lenght | Mean Difference  (I-J) | Standart  Error | Significance | 95% Confidence Interval | |
|  |  |  |  |  | Lower Limit | Upper Limit |
| No extension | 5 cm | ,40000* | ,06325 | ,000 | ,2230 | ,5770 |
|  | 10 cm | ,63333* | ,06325 | ,000 | ,4563 | ,8104 |
|  | 15 cm | ,96667* | ,06325 | ,000 | ,7896 | 1,1437 |
| 5 cm | No extension | -,40000* | ,06325 | ,000 | -,5770 | -,2230 |
|  | 10 cm | ,23333* | ,06325 | ,007 | ,0563 | ,4104 |
|  | 15 cm | ,56667* | ,06325 | ,000 | ,3896 | ,7437 |
| 10 cm | No extension | -,63333* | ,06325 | ,000 | -,8104 | -,4563 |
|  | 5 cm | -,23333* | ,06325 | ,007 | -,4104 | -,0563 |
|  | 15 cm | ,33333* | ,06325 | ,000 | ,1563 | ,5104 |
| 15 cm | No extension | -,96667* | ,06325 | ,000 | -1,1437 | -,7896 |
|  | 5 cm | -,56667* | ,06325 | ,000 | -,7437 | -,3896 |
|  | 10 cm | -,33333* | ,06325 | ,000 | -,5104 | -,1563 |

**Table 53** The farthest satellite entry holes at 200cm distance on Extension Lenghts

| Extension  Lenght | Number  Of  Shots | Arithmetic Mean  (cm) | Standard Deviation | Standard Error | Confidence Interval (%95)  Lower Limit | Confidence Interval (%95)  Upper Limit |
| --- | --- | --- | --- | --- | --- | --- |
| No extension | 3 | 4,83 | 0,153 | 0,088 | 4,66 | 5,01 |
| 5 cm | 3 | 4,53 | 0,058 | 0,033 | 4,47 | 4,6 |
| 10 cm | 3 | 4,3 | 0,1 | 0,058 | 4,19 | 4,41 |
| 15 cm | 3 | 4,1 | 0,1 | 0,058 | 3,99 | 4,21 |
| Total | 12 | 4,44 | 0,3 | 0,087 | 4,27 | 4,61 |

**Table 54** Variance Analysis of the farthest satellite entry holes at 200cm distance based on Extension Lengths.

| **ANOVA** | | | | | |
| --- | --- | --- | --- | --- | --- |
| Measurement on Target | |  |  |  |  |
|  | Sums of Squares  **(SS)** | Degrees of Freedom **(df)** | Means Squares **(MS)** | F Value | Significance |
| Between Groups | ,896 | 3 | ,299 | 25,595 | ,000 |
| In Groups | ,093 | 8 | ,012 |  |  |
| Total | ,989 | 11 |  |  |  |

**Table 55** Satellite Entry Holes Multiple Comparison Analysis at 200cm Distances Based on Extension Lengths.

| **Multiple Comparison** | | | | | | |
| --- | --- | --- | --- | --- | --- | --- |
| Measurement on Target  Tukey HSD | | |  |  |  |  |
| (I) Extension  Length | (J) Extension  Length | Mean Difference  (I-J) | Standard  Error | Significance | 95% Confidence Interval | |
|  |  |  |  |  | Lower Limit | Upper Limit |
| No extension | 5 cm | ,30000* | ,08819 | ,038 | ,0176 | ,5824 |
|  | 10 cm | ,53333* | ,08819 | ,001 | ,2509 | ,8158 |
|  | 15 cm | ,73333* | ,08819 | ,000 | ,4509 | 1,0158 |
| 5 cm | No extension | -,30000* | ,08819 | ,038 | -,5824 | -,0176 |
|  | 10 cm | ,23333 | ,08819 | ,110 | -,0491 | ,5158 |
|  | 15 cm | ,43333* | ,08819 | ,005 | ,1509 | ,7158 |
| 10 cm | No extension | -,53333* | ,08819 | ,001 | -,8158 | -,2509 |
|  | 5 cm | -,23333 | ,08819 | ,110 | -,5158 | ,0491 |
|  | 15 cm | ,20000 | ,08819 | ,185 | -,0824 | ,4824 |
| 15 cm | No extension | -,73333* | ,08819 | ,000 | -1,0158 | -,4509 |
|  | 5 cm | -,43333* | ,08819 | ,005 | -,7158 | -,1509 |
|  | 10 cm | -,20000 | ,08819 | ,185 | -,4824 | ,0824 |

**Table 56** Entry hole diameters based on extension length at 300cm range.

| Measurement  Type | Extension  Lenght | Number  Of  Shots | Arithmetic Mean  (cm) | Standard Deviation | Standard Error | Confidence Interval (%95)  Lower Limit | Confidence Interval (%95)  Upper Limit |
| --- | --- | --- | --- | --- | --- | --- | --- |
| Horizantal | No extension | 3 | 3,97 | 0,153 | 0,09 | 3,79 | 4,14 |
|  | 5 cm | 3 | 3,13 | 0,208 | 0,12 | 2,9 | 3,37 |
|  | 10 cm | 3 | 2,33 | 0,058 | 0,03 | 2,27 | 2,4 |
|  | 15 cm | 3 | 1,73 | 0,058 | 0,03 | 1,67 | 1,8 |
|  | Total | 12 | 2,79 | 0,886 | 0,26 | 2,29 | 3,29 |
| Vertical | No extension | 3 | 3,9 | 0,1 | 0,06 | 3,79 | 4,01 |
|  | 5 cm | 3 | 3,2 | 0,173 | 0,1 | 3 | 3,4 |
|  | 10 cm | 3 | 2,33 | 0,153 | 0,09 | 2,16 | 2,51 |
|  | 15 cm | 3 | 1,73 | 0,115 | 0,07 | 1,6 | 1,86 |
|  | Total | 12 | 2,79 | 0,87 | 0,25 | 2,3 | 3,28 |
| Mean | No extension | 3 | 3,93 | 0,121 | 0,05 | 3,84 | 4,03 |
|  | 5 cm | 3 | 3,17 | 0,175 | 0,07 | 3,03 | 3,31 |
|  | 10 cm | 3 | 2,33 | 0,103 | 0,04 | 2,25 | 2,42 |
|  | 15 cm | 3 | 1,73 | 0,082 | 0,03 | 1,67 | 1,8 |
|  | Total | 12 | 2,79 | 0,859 | 0,18 | 2,45 | 3,14 |

**Table 57** Entry Hole Variance Analysis at 300cm Distance based on Extension Lengths.

| **ANOVA** | | | | | |
| --- | --- | --- | --- | --- | --- |
| Measurement on Target | |  |  |  |  |
|  | Sums of Squares  **(SS)** | Degrees of Freedom **(df)** | Means Squares **(MS)** | F Value | Significance |
| Between Groups | 16,645 | 3 | 5,548 | 354,149 | ,000 |
| In Groups | ,313 | 20 | ,016 |  |  |
| Total | 16,958 | 23 |  |  |  |

| **Table 58** Entry Hole Diameters Multiple Comparisons at 300cm Distance based on Extension Lengths..  **Multiple Comparison** | | | | | | |
| --- | --- | --- | --- | --- | --- | --- |
| Measurement on Target  Tukey HSD | | |  |  |  |  |
| (I) Extension  Lenght | (J) Extension  Lenght | Mean Difference  (I-J) | Standart  Error | Significance | 95% Confidence Interval | |
|  |  |  |  |  | Lower Limit | Upper Limit |
| No extension | 5 cm | ,76667* | ,07226 | ,000 | ,5644 | ,9689 |
|  | 10 cm | 1,60000* | ,07226 | ,000 | 1,3977 | 1,8023 |
|  | 15 cm | 2,20000* | ,07226 | ,000 | 1,9977 | 2,4023 |
| 5 cm | No extension | -,76667* | ,07226 | ,000 | -,9689 | -,5644 |
|  | 10 cm | ,83333* | ,07226 | ,000 | ,6311 | 1,0356 |
|  | 15 cm | 1,43333* | ,07226 | ,000 | 1,2311 | 1,6356 |
| 10 cm | No extension | -1,60000* | ,07226 | ,000 | -1,8023 | -1,3977 |
|  | 5 cm | -,83333* | ,07226 | ,000 | -1,0356 | -,6311 |
|  | 15 cm | ,60000* | ,07226 | ,000 | ,3977 | ,8023 |
| 15 cm | No extension | -2,20000* | ,07226 | ,000 | -2,4023 | -1,9977 |
|  | 5 cm | -1,43333* | ,07226 | ,000 | -1,6356 | -1,2311 |
|  | 10 cm | -,60000* | ,07226 | ,000 | -,8023 | -,3977 |

**Table 59** Pellet Distribution on at 300cm distances based on Extension Lengths.

| Measurement  Type | Extension  Lenght | Number  Of  Shots | Arithmetic Mean  (cm) | Standard Deviation | Standard Error | Confidence Interval (%95)  Lower Limit | Confidence Interval (%95)  Upper Limit |
| --- | --- | --- | --- | --- | --- | --- | --- |
| Horizantal | No extension | 3 | 14,2 | 0,608 | 0,351 | 13,51 | 14,89 |
|  | 5 cm | 3 | 12,4 | 0,4 | 0,231 | 11,95 | 12,85 |
|  | 10 cm | 3 | 12,2 | 0,361 | 0,208 | 11,79 | 12,61 |
|  | 15 cm | 3 | 10,77 | 0,252 | 0,145 | 10,48 | 11,05 |
|  | Total | 12 | 12,39 | 1,324 | 0,382 | 11,64 | 13,14 |
| Vertical | No extension | 3 | 14 | 0,2 | 0,115 | 13,77 | 14,23 |
|  | 5 cm | 3 | 12,6 | 0,1 | 0,058 | 12,49 | 12,71 |
|  | 10 cm | 3 | 12 | 0,1 | 0,058 | 11,89 | 12,11 |
|  | 15 cm | 3 | 10,97 | 0,252 | 0,145 | 10,68 | 11,25 |
|  | Total | 12 | 12,39 | 1,156 | 0,334 | 11,74 | 13,05 |
| Mean | No extension | 3 | 14,1 | 0,42 | 0,171 | 13,76 | 14,44 |
|  | 5 cm | 3 | 12,5 | 0,283 | 0,115 | 12,27 | 12,73 |
|  | 10 cm | 3 | 12,1 | 0,261 | 0,106 | 11,89 | 12,31 |
|  | 15 cm | 3 | 10,87 | 0,25 | 0,102 | 10,67 | 11,07 |
|  | Total | 12 | 12,39 | 1,215 | 0,248 | 11,91 | 12,88 |

**Table 60** Pellet Distribution Diameter Variance Analysis at 300cm Distances Based on Extension Lengths.

| **ANOVA** | | | | | |
| --- | --- | --- | --- | --- | --- |
| Measurement on Target | |  |  |  |  |
|  | Sums of Squares  **(SS)** | Degrees of Freedom **(df)** | Means Squares **(MS)** | F Value | Significance |
| Between Groups | 32,045 | 3 | 10,682 | 110,500 | ,000 |
| In Groups | 1,933 | 20 | ,097 |  |  |
| Total | 33,978 | 23 |  |  |  |

**Çizelge 61** Pellet Distribution Diameter Multiple Comparison Analysis at 300cm Distances Based on Extension Lengths.

| **Multiple Comparison** | | | | | | |
| --- | --- | --- | --- | --- | --- | --- |
|  | | |  |  |  |  |
| Measurement on Target  Tukey HSD | | |  |  |  |  |
| (I) Extension  Lenght | (J) Extension  Lenght | Mean Difference  (I-J) | Standart  Error | Significance | 95% Confidence Interval | |
|  |  |  |  |  | Lower Limit | Upper Limit |
| No extension | 5 cm | 1,60000* | ,17951 | ,000 | 1,0976 | 2,1024 |
|  | 10 cm | 2,00000* | ,17951 | ,000 | 1,4976 | 2,5024 |
|  | 15 cm | 3,23333* | ,17951 | ,000 | 2,7309 | 3,7358 |
| 5 cm | No extension | -1,60000* | ,17951 | ,000 | -2,1024 | -1,0976 |
|  | 10 cm | ,40000 | ,17951 | ,150 | -,1024 | ,9024 |
|  | 15 cm | 1,63333* | ,17951 | ,000 | 1,1309 | 2,1358 |
| 10 cm | No extension | -2,00000* | ,17951 | ,000 | -2,5024 | -1,4976 |
|  | 5 cm | -,40000 | ,17951 | ,150 | -,9024 | ,1024 |
|  | 15 cm | 1,23333* | ,17951 | ,000 | ,7309 | 1,7358 |
| 15 cm | No extension | -3,23333* | ,17951 | ,000 | -3,7358 | -2,7309 |
|  | 5 cm | -1,63333* | ,17951 | ,000 | -2,1358 | -1,1309 |
|  | 10 cm | -1,23333* | ,17951 | ,000 | -1,7358 | -,7309 |

**Table 62** The farthest satellite entry holes at 300cm distance based on Extension Lenghts

| Extension  Lenght | Number  Of  Shots | Arithmetic Mean  (cm) | Standard Deviation | Standard Error | Confidence Interval (%95)  Lower Limit | Confidence Interval (%95)  Upper Limit |
| --- | --- | --- | --- | --- | --- | --- |
| No extension | 3 | 8,8 | 0,3 | 0,173 | 8,46 | 9,14 |
| 5 cm | 3 | 7,67 | 0,208 | 0,12 | 7,43 | 7,9 |
| 10 cm | 3 | 7,33 | 0,153 | 0,088 | 7,16 | 7,51 |
| 15 cm | 3 | 7,1 | 0,1 | 0,058 | 6,99 | 7,21 |
| Total | 12 | 7,73 | 0,703 | 0,203 | 7,33 | 8,12 |

**Çizelge 63** Satellite Entry Hole Variance Analysis at 300cm Distances Based on Extension Lengths.

| **ANOVA** | | | | | |
| --- | --- | --- | --- | --- | --- |
| Measurement on Target | |  |  |  |  |
|  | Sums of Squares  **(SS)** | Degrees of Freedom **(df)** | Means Squares **(MS)** | F Value | Significance |
| Between Groups | 5,109 | 3 | 1,703 | 40,873 | ,000 |
| In Groups | ,333 | 8 | ,042 |  |  |
| Total | 5,442 | 11 |  |  |  |

**Table 64** The farthest satellite entry holes Multiple Comparison Analysis at 300cm Distances Based on Extension Lengths.

| **Multible Comparison** | | | | | | |
| --- | --- | --- | --- | --- | --- | --- |
| Measurement on Target  Tukey HSD | | |  |  |  |  |
| (I) Extension  Length | (J) Extension  Length | Mean Difference  (I-J) | Standard  Error | Significance | 95% Confidence Interval | |
|  |  |  |  |  | Lower Limit | Upper Limit |
| No extension | 5 cm | 1,13333* | ,16667 | ,001 | ,5996 | 1,6671 |
|  | 10 cm | 1,46667* | ,16667 | ,000 | ,9329 | 2,0004 |
|  | 15 cm | 1,70000* | ,16667 | ,000 | 1,1663 | 2,2337 |
| 5 cm | No extension | -1,13333* | ,16667 | ,001 | -1,6671 | -,5996 |
|  | 10 cm | ,33333 | ,16667 | ,264 | -,2004 | ,8671 |
|  | 15 cm | ,56667* | ,16667 | ,038 | ,0329 | 1,1004 |
| 10 cm | No extension | -1,46667* | ,16667 | ,000 | -2,0004 | -,9329 |
|  | 5 cm | -,33333 | ,16667 | ,264 | -,8671 | ,2004 |
|  | 15 cm | ,23333 | ,16667 | ,533 | -,3004 | ,7671 |
| 15 cm | No extension | -1,70000* | ,16667 | ,000 | -2,2337 | -1,1663 |
|  | 5 cm | -,56667* | ,16667 | ,038 | -1,1004 | -,0329 |
|  | 10 cm | -,23333 | ,16667 | ,533 | -,7671 | ,3004 |

**Çizelge 65** Entry hole diameters based on extension length at 400cm range

| Measurement  Type | Extension  Lenght | Number  Of  Shots | Arithmetic Mean  (cm) | Standard Deviation | Standard Error | Confidence Interval (%95)  Lower Limit | Confidence Interval (%95)  Upper Limit |
| --- | --- | --- | --- | --- | --- | --- | --- |
| Horizantal | No extension | 3 | 2,33 | 0,058 | 0,03 | 2,27 | 2,4 |
|  | 5 cm | 3 | 1,97 | 0,058 | 0,03 | 1,9 | 2,03 |
|  | 10 cm | 3 | 1,8 | 0,1 | 0,06 | 1,69 | 1,91 |
|  | 15 cm | 3 | 1,57 | 0,058 | 0,03 | 1,5 | 1,63 |
|  | Total | 12 | 1,92 | 0,298 | 0,09 | 1,75 | 2,09 |
| Vertical | No extension | 3 | 2,33 | 0,058 | 0,03 | 2,27 | 2,4 |
|  | 5 cm | 3 | 1,97 | 0,115 | 0,07 | 1,84 | 2,1 |
|  | 10 cm | 3 | 1,73 | 0,058 | 0,03 | 1,67 | 1,8 |
|  | 15 cm | 3 | 1,57 | 0,115 | 0,07 | 1,44 | 1,7 |
|  | Total | 12 | 1,9 | 0,31 | 0,09 | 1,72 | 2,08 |
| Mean | No extension | 3 | 2,33 | 0,052 | 0,02 | 2,29 | 2,37 |
|  | 5 cm | 3 | 1,97 | 0,082 | 0,03 | 1,9 | 2,03 |
|  | 10 cm | 3 | 1,77 | 0,082 | 0,03 | 1,7 | 1,83 |
|  | 15 cm | 3 | 1,57 | 0,082 | 0,03 | 1,5 | 1,63 |
|  | Total | 12 | 1,91 | 0,298 | 0,06 | 1,79 | 2,03 |

**Çizelge 66** Entry Hole Variance Analysis at 400cm Distance based on Extension Lengths.

| **ANOVA** | | | | | |
| --- | --- | --- | --- | --- | --- |
| Measurement on Target | |  |  |  |  |
|  | Sums of Squares  **(SS)** | Degrees of Freedom **(df)** | Means Squares **(MS)** | F Value | Significance |
| Between Groups | 1,925 | 3 | ,642 | 113,235 | ,000 |
| In Groups | ,113 | 20 | ,006 |  |  |
| Total | 2,038 | 23 |  |  |  |

**Çizelge 67** Entry Hole Diameters Multiple Comparisons at 400cm Distance based on Extension Lengths.

| **Multiple Comparison** | | | | | | |
| --- | --- | --- | --- | --- | --- | --- |
| Measurement on Target  Tukey HSD | | |  |  |  |  |
| (I) Extension  Lenght | (J) Extension  Lenght | Mean Difference  (I-J) | Standart  Error | Significance | 95% Confidence Interval | |
|  |  |  |  |  | Lower Limit | Upper Limit |
| No extension | 5 cm | ,36667* | ,04346 | ,000 | ,2450 | ,4883 |
|  | 10 cm | ,56667* | ,04346 | ,000 | ,4450 | ,6883 |
|  | 15 cm | ,76667* | ,04346 | ,000 | ,6450 | ,8883 |
| 5 cm | No extension | -,36667* | ,04346 | ,000 | -,4883 | -,2450 |
|  | 10 cm | ,20000* | ,04346 | ,001 | ,0784 | ,3216 |
|  | 15 cm | ,40000* | ,04346 | ,000 | ,2784 | ,5216 |
| 10 cm | No extension | -,56667* | ,04346 | ,000 | -,6883 | -,4450 |
|  | 5 cm | -,20000* | ,04346 | ,001 | -,3216 | -,0784 |
|  | 15 cm | ,20000* | ,04346 | ,001 | ,0784 | ,3216 |
| 15 cm | No extension | -,76667* | ,04346 | ,000 | -,8883 | -,6450 |
|  | 5 cm | -,40000* | ,04346 | ,000 | -,5216 | -,2784 |
|  | 10 cm | -,20000* | ,04346 | ,001 | -,3216 | -,0784 |

**Çizelge 68** Pellet Distribution on at 400cm distances based on Extension Lengths.

| Measurement  Type | Extension  Lenght | Number  Of  Shots | Arithmetic Mean  (cm) | Standard Deviation | Standard Error | Confidence Interval (%95)  Lower Limit | Confidence Interval (%95)  Upper Limit |
| --- | --- | --- | --- | --- | --- | --- | --- |
| Horizantal | No extension | 3 | 20,9 | 0,361 | 0,208 | 20,49 | 21,31 |
|  | 5 cm | 3 | 18,1 | 0,1 | 0,058 | 17,99 | 18,21 |
|  | 10 cm | 3 | 17,2 | 0,265 | 0,153 | 16,9 | 17,5 |
|  | 15 cm | 3 | 16,17 | 0,306 | 0,176 | 15,82 | 16,51 |
|  | Total | 12 | 18,09 | 1,853 | 0,535 | 17,04 | 19,14 |
| Vertical | No extension | 3 | 21,1 | 0,3 | 0,173 | 20,76 | 21,44 |
|  | 5 cm | 3 | 18,03 | 0,153 | 0,088 | 17,86 | 18,21 |
|  | 10 cm | 3 | 17,13 | 0,208 | 0,12 | 16,9 | 17,37 |
|  | 15 cm | 3 | 15,97 | 0,153 | 0,088 | 15,79 | 16,14 |
|  | Total | 12 | 18,06 | 1,996 | 0,576 | 16,93 | 19,19 |
| Mean | No extension | 3 | 21 | 0,316 | 0,129 | 20,75 | 21,25 |
|  | 5 cm | 3 | 18,07 | 0,121 | 0,049 | 17,97 | 18,16 |
|  | 10 cm | 3 | 17,17 | 0,216 | 0,088 | 16,99 | 17,34 |
|  | 15 cm | 3 | 16,07 | 0,242 | 0,099 | 15,87 | 16,26 |
|  | Total | 12 | 18,08 | 1,883 | 0,384 | 17,32 | 18,83 |

**Table 69** Pellet Distribution Diameter Variance Analysis at 400cm Distances Based on Extension Lengths.

| **ANOVA** | | | | | |
| --- | --- | --- | --- | --- | --- |
| Measurement on Target | |  |  |  |  |
|  | Sums of Squares  **(SS)** | Degrees of Freedom **(df)** | Means Squares **(MS)** | F Value | Significance |
| Between Groups | 80,485 | 3 | 26,828 | 487,788 | ,000 |
| In Groups | 1,100 | 20 | ,055 |  |  |
| Total | 81,585 | 23 |  |  |  |

**Table 70** Pellet Distribution Diameter Multiple Comparison Analysis at 400cm Distances Based on Extension Lengths.

| **Multiple Comparison** | | | | | | |
| --- | --- | --- | --- | --- | --- | --- |
| Measurement on Target  Tukey HSD | | |  |  |  |  |
| (I) Extension  Lenght | (J) Extension  Lenght | Mean Difference  (I-J) | Standart  Error | Significance | 95% Confidence Interval | |
|  |  |  |  |  | Lower Limit | Upper Limit |
| No extension | 5 cm | 2,93333* | ,13540 | ,000 | 2,5544 | 3,3123 |
|  | 10 cm | 3,83333* | ,13540 | ,000 | 3,4544 | 4,2123 |
|  | 15 cm | 4,93333* | ,13540 | ,000 | 4,5544 | 5,3123 |
| 5 cm | No extension | -2,93333* | ,13540 | ,000 | -3,3123 | -2,5544 |
|  | 10 cm | ,90000* | ,13540 | ,000 | ,5210 | 1,2790 |
|  | 15 cm | 2,00000* | ,13540 | ,000 | 1,6210 | 2,3790 |
| 10 cm | No extension | -3,83333* | ,13540 | ,000 | -4,2123 | -3,4544 |
|  | 5 cm | -,90000* | ,13540 | ,000 | -1,2790 | -,5210 |
|  | 15 cm | 1,10000* | ,13540 | ,000 | ,7210 | 1,4790 |
| 15 cm | No extension | -4,93333* | ,13540 | ,000 | -5,3123 | -4,5544 |
|  | 5 cm | -2,00000* | ,13540 | ,000 | -2,3790 | -1,6210 |
|  | 10 cm | -1,10000* | ,13540 | ,000 | -1,4790 | -,7210 |

**Table 71** The farthest satellite entry holes at 400cm distance based on Extension Lenghts.

| Extension  Lenght | Number  Of  Shots | Arithmetic Mean  (cm) | Standard Deviation | Standard Error | Confidence Interval (%95)  Lower Limit | Confidence Interval (%95)  Upper Limit |
| --- | --- | --- | --- | --- | --- | --- |
| No Extension | 3 | 12,2 | 1,179 | 0,681 | 10,87 | 13,53 |
| 5 cm | 3 | 11,2 | 0,361 | 0,208 | 10,79 | 11,61 |
| 10 cm | 3 | 10,97 | 0,681 | 0,393 | 10,2 | 11,74 |
| 15 cm | 3 | 11,07 | 0,513 | 0,296 | 10,49 | 11,65 |
| Total | 12 | 11,36 | 0,821 | 0,237 | 10,89 | 11,82 |

**Table 72** The farthest satellite entry holes Variance Analysis at 400cm Distances Based on Extension Lengths.

| **ANOVA** | | | | | |
| --- | --- | --- | --- | --- | --- |
| Measurement on Target | |  |  |  |  |
|  | Sums of Squares  **(SS)** | Degrees of Freedom **(df)** | Means Squares **(MS)** | F Value | Significance |
| Between Groups | 2,916 | 3 | ,972 | 1,730 | ,238 |
| In Groups | 4,493 | 8 | ,562 |  |  |
| Total | 7,409 | 11 |  |  |  |

**Table 73** The farthest satellite entry holes Multiple Comparison Analysis at 400cm Distances Based on Extension Lengths.

| **Multiple Comparison** | | | | | | |
| --- | --- | --- | --- | --- | --- | --- |
| Measurement on Target  Tukey HSD | | |  |  |  |  |
| (I) Extension  Lenght | (J) Extension  Lenght | Mean Difference  (I-J) | Standart  Error | Significance | 95% Confidence Interval | |
|  |  |  |  |  | Lower Limit | Upper Limit |
| No extension | 5 cm | 1,00000 | ,61192 | ,414 | -,9596 | 2,9596 |
|  | 10 cm | 1,23333 | ,61192 | ,259 | -,7262 | 3,1929 |
|  | 15 cm | 1,13333 | ,61192 | ,319 | -,8262 | 3,0929 |
| 5 cm | No extension | -1,00000 | ,61192 | ,414 | -2,9596 | ,9596 |
|  | 10 cm | ,23333 | ,61192 | ,980 | -1,7262 | 2,1929 |
|  | 15 cm | ,13333 | ,61192 | ,996 | -1,8262 | 2,0929 |
| 10 cm | No extension | -1,23333 | ,61192 | ,259 | -3,1929 | ,7262 |
|  | 5 cm | -,23333 | ,61192 | ,980 | -2,1929 | 1,7262 |
|  | 15 cm | -,10000 | ,61192 | ,998 | -2,0596 | 1,8596 |
| 15 cm | No extension | -1,13333 | ,61192 | ,319 | -3,0929 | ,8262 |
|  | 5 cm | -,13333 | ,61192 | ,996 | -2,0929 | 1,8262 |
|  | 10 cm | ,10000 | ,61192 | ,998 | -1,8596 | 2,0596 |

**Table 74** Entry hole diameters based on extension length at 500cm range

| Measurement  Type | Extension  Lenght | Number  Of  Shots | Arithmetic Mean  (cm) | Standard Deviation | Standard Error | Confidence Interval (%95)  Lower Limit | Confidence Interval (%95)  Upper Limit |
| --- | --- | --- | --- | --- | --- | --- | --- |
| Horizantal | No extension | 3 | 2,4 | 0,1 | 0,06 | 2,29 | 2,51 |
|  | 5 cm | 3 | 2,23 | 0,115 | 0,07 | 2,1 | 2,36 |
|  | 10 cm | 3 | 2,33 | 0,153 | 0,09 | 2,16 | 2,51 |
|  | 15 cm | 3 | 2,13 | 0,153 | 0,09 | 1,96 | 2,31 |
|  | Total | 12 | 2,28 | 0,154 | 0,04 | 2,19 | 2,36 |
| Vertical | No extension | 3 | 2,57 | 0,153 | 0,09 | 2,39 | 2,74 |
|  | 5 cm | 3 | 2,33 | 0,115 | 0,07 | 2,2 | 2,46 |
|  | 10 cm | 3 | 2,17 | 0,115 | 0,07 | 2,04 | 2,3 |
|  | 15 cm | 3 | 2,33 | 0,058 | 0,03 | 2,27 | 2,4 |
|  | Total | 12 | 2,35 | 0,178 | 0,05 | 2,25 | 2,45 |
| Mean | No extension | 3 | 2,48 | 0,147 | 0,06 | 2,37 | 2,6 |
|  | 5 cm | 3 | 2,28 | 0,117 | 0,05 | 2,19 | 2,38 |
|  | 10 cm | 3 | 2,25 | 0,152 | 0,06 | 2,13 | 2,37 |
|  | 15 cm | 3 | 2,23 | 0,151 | 0,06 | 2,11 | 2,35 |
|  | Total | 12 | 2,31 | 0,168 | 0,03 | 2,25 | 2,38 |

**Table 75** Entry Hole Variance Analysis at 500cm Distance based on Extension Lengths.

| **ANOVA** | | | | | |
| --- | --- | --- | --- | --- | --- |
| Measurement on Target | |  |  |  |  |
|  | Sums of Squares  **(SS)** | Degrees of Freedom **(df)** | Means Squares **(MS)** | F Value | Significance |
| Between Groups | ,241 | 3 | ,080 | 3,971 | ,023 |
| In Groups | ,405 | 20 | ,020 |  |  |
| Total | ,646 | 23 |  |  |  |

**Table 76** Entry Hole Diameters Multiple Comparisons at 500cm Distance based on Extension Lengths..

| **Multiple Comparison** | | | | | | |
| --- | --- | --- | --- | --- | --- | --- |
| Measurement on Target  Tukey HSD | | |  |  |  |  |
| (I) Extension  Lenght | (J) Extension  Lenght | Mean Difference  (I-J) | Standart  Error | Significance | 95% Confidence Interval | |
|  |  |  |  |  | Lower Limit | Upper Limit |
| No extension | 5 cm | ,20000 | ,08216 | ,103 | -,0300 | ,4300 |
|  | 10 cm | ,23333* | ,08216 | ,046 | ,0034 | ,4633 |
|  | 15 cm | ,25000* | ,08216 | ,030 | ,0200 | ,4800 |
| 5 cm | No extension | -,20000 | ,08216 | ,103 | -,4300 | ,0300 |
|  | 10 cm | ,03333 | ,08216 | ,977 | -,1966 | ,2633 |
|  | 15 cm | ,05000 | ,08216 | ,928 | -,1800 | ,2800 |
| 10 cm | No extension | -,23333* | ,08216 | ,046 | -,4633 | -,0034 |
|  | 5 cm | -,03333 | ,08216 | ,977 | -,2633 | ,1966 |
|  | 15 cm | ,01667 | ,08216 | ,997 | -,2133 | ,2466 |
| 15 cm | No extension | -,25000* | ,08216 | ,030 | -,4800 | -,0200 |
|  | 5 cm | -,05000 | ,08216 | ,928 | -,2800 | ,1800 |
|  | 10 cm | -,01667 | ,08216 | ,997 | -,2466 | ,2133 |

**Table 77** Pellet Distribution on at 500cm distances based on Extension Lengths.

| Measurement  Type | Extension  Lenght | Number  Of  Shots | Arithmetic Mean  (cm) | Standard Deviation | Standard Error | Confidence Interval (%95)  Lower Limit | Confidence Interval (%95)  Upper Limit |
| --- | --- | --- | --- | --- | --- | --- | --- |
| Horizantal | No extension | 3 | 25,8 | 1,249 | 0,721 | 24,39 | 27,21 |
|  | 5 cm | 3 | 23,07 | 1,305 | 0,754 | 21,59 | 24,54 |
|  | 10 cm | 3 | 21,33 | 0,862 | 0,498 | 20,36 | 22,31 |
|  | 15 cm | 3 | 19,77 | 0,404 | 0,233 | 19,31 | 20,22 |
|  | Total | 12 | 22,49 | 2,495 | 0,72 | 21,08 | 23,9 |
| Vertical | No extension | 3 | 25,33 | 0,252 | 0,145 | 25,05 | 25,62 |
|  | 5 cm | 3 | 23,33 | 1,069 | 0,617 | 22,12 | 24,54 |
|  | 10 cm | 3 | 21,87 | 0,666 | 0,384 | 21,11 | 22,62 |
|  | 15 cm | 3 | 20,07 | 0,252 | 0,145 | 19,78 | 20,35 |
|  | Total | 12 | 22,65 | 2,095 | 0,605 | 21,46 | 23,84 |
| Mean | No extension | 3 | 25,57 | 0,845 | 0,345 | 24,89 | 26,24 |
|  | 5 cm | 3 | 23,2 | 1,077 | 0,44 | 22,34 | 24,06 |
|  | 10 cm | 3 | 21,6 | 0,748 | 0,306 | 21 | 22,2 |
|  | 15 cm | 3 | 19,92 | 0,343 | 0,14 | 19,64 | 20,19 |
|  | Total | 12 | 22,57 | 2,255 | 0,46 | 21,67 | 23,47 |

**Table 78** Pellet Distribution Diameter Variance Analysis at 500cm Distances Based on Extension Lengths.

| **ANOVA** | | | | | |
| --- | --- | --- | --- | --- | --- |
| Measurement on Target | |  |  |  |  |
|  | Sums of Squares  **(SS)** | Degrees of Freedom **(df)** | Means Squares **(MS)** | F Value | Significance |
| Between Groups | 104,148 | 3 | 34,716 | 54,407 | ,000 |
| In Groups | 12,762 | 20 | ,638 |  |  |
| Total | 116,910 | 23 |  |  |  |

**Table 79** Pellet Distribution Diameter Multiple Comparison Analysis at 500cm Distances Based on Extension Lengths..

| **Multiple Comparison** | | | | | | |
| --- | --- | --- | --- | --- | --- | --- |
| Measurement on Target  Tukey HSD | | |  |  |  |  |
| (I) Extension  Lenght | (J) Extension  Lenght | Mean Difference  (I-J) | Standart  Error | Significance | 95% Confidence Interval | |
|  |  |  |  |  | Lower Limit | Upper Limit |
| No extension | 5 cm | 2,36667* | ,46119 | ,000 | 1,0758 | 3,6575 |
|  | 10 cm | 3,96667* | ,46119 | ,000 | 2,6758 | 5,2575 |
|  | 15 cm | 5,65000* | ,46119 | ,000 | 4,3592 | 6,9408 |
| 5 cm | No extension | -2,36667* | ,46119 | ,000 | -3,6575 | -1,0758 |
|  | 10 cm | 1,60000* | ,46119 | ,012 | ,3092 | 2,8908 |
|  | 15 cm | 3,28333* | ,46119 | ,000 | 1,9925 | 4,5742 |
| 10 cm | No extension | -3,96667* | ,46119 | ,000 | -5,2575 | -2,6758 |
|  | 5 cm | -1,60000* | ,46119 | ,012 | -2,8908 | -,3092 |
|  | 15 cm | 1,68333* | ,46119 | ,008 | ,3925 | 2,9742 |
| 15 cm | No extension | -5,65000* | ,46119 | ,000 | -6,9408 | -4,3592 |
|  | 5 cm | -3,28333* | ,46119 | ,000 | -4,5742 | -1,9925 |
|  | 10 cm | -1,68333* | ,46119 | ,008 | -2,9742 | -,3925 |

**Table 80** The farthest satellite entry holes at 500cm distance based on Extension Lenghts.

| Extension  Lenght | Number  Of  Shots | Arithmetic Mean  (cm) | Standard Deviation | Standard Error | Confidence Interval (%95)  Lower Limit | Confidence Interval (%95)  Upper Limit |
| --- | --- | --- | --- | --- | --- | --- |
| No Extension | 3 | 15,73 | 0,586 | 0,338 | 15,07 | 16,4 |
| 5 cm | 3 | 13,6 | 1,825 | 1,054 | 11,54 | 15,66 |
| 10 cm | 3 | 14,7 | 0,436 | 0,252 | 14,21 | 15,19 |
| 15 cm | 3 | 14,57 | 1,531 | 0,884 | 12,83 | 16,3 |
| Total | 12 | 14,65 | 1,324 | 0,382 | 13,9 | 15,4 |

**Table 81** The farthest satellite entry holes Variance Analysis at 500cm Distances Based on Extension Lengths.

| **ANOVA** | | | | | |
| --- | --- | --- | --- | --- | --- |
| Measurement on Target | |  |  |  |  |
|  | Sums of Squares  **(SS)** | Degrees of Freedom **(df)** | Means Squares **(MS)** | F Value | Significance |
| Between Groups | 6,857 | 3 | 2,286 | 1,473 | ,293 |
| In Groups | 12,413 | 8 | 1,552 |  |  |
| Total | 19,270 | 11 |  |  |  |

**Table 82** The farthest satellite entry holes Multiple Comparison Analysis at 500cm Distances Based on Extension Lengths

| **Multiple Comparison** | | | | | | |
| --- | --- | --- | --- | --- | --- | --- |
| Measurement on Target  Tukey HSD | | |  |  |  |  |
| (I) Extension  Lenght | (J) Extension  Lenght | Mean Difference  (I-J) | Standard  Error | Significance | 95% Confidence Interval | |
|  |  |  |  |  | Lower Limit | Upper Limit |
| No extension | 5 cm | 2,13333 | 1,01708 | ,232 | -1,1237 | 5,3904 |
|  | 10 cm | 1,03333 | 1,01708 | ,745 | -2,2237 | 4,2904 |
|  | 15 cm | 1,16667 | 1,01708 | ,673 | -2,0904 | 4,4237 |
| 5 cm | No extension | -2,13333 | 1,01708 | ,232 | -5,3904 | 1,1237 |
|  | 10 cm | -1,10000 | 1,01708 | ,710 | -4,3570 | 2,1570 |
|  | 15 cm | -,96667 | 1,01708 | ,780 | -4,2237 | 2,2904 |
| 10 cm | No extension | -1,03333 | 1,01708 | ,745 | -4,2904 | 2,2237 |
|  | 5 cm | 1,10000 | 1,01708 | ,710 | -2,1570 | 4,3570 |
|  | 15 cm | ,13333 | 1,01708 | ,999 | -3,1237 | 3,3904 |
| 15 cm | No extension | -1,16667 | 1,01708 | ,673 | -4,4237 | 2,0904 |
|  | 5 cm | ,96667 | 1,01708 | ,780 | -2,2904 | 4,2237 |
|  | 10 cm | -,13333 | 1,01708 | ,999 | -3,3904 | 3,1237 |

**Table 83** Entry hole diameters based on extension length at 700cm range

| Measurement  Type | Extension  Lenght | Number  Of  Shots | Arithmetic Mean  (cm) | Standard Deviation | Standard Error | Confidence Interval (%95)  Lower Limit | Confidence Interval (%95)  Upper Limit |
| --- | --- | --- | --- | --- | --- | --- | --- |
| Horizantal | No extension | 3 | 2,2 | 0,1 | 0,06 | 2,09 | 2,31 |
|  | 5 cm | 3 | 2,17 | 0,058 | 0,03 | 2,1 | 2,23 |
|  | 10 cm | 3 | 2,17 | 0,115 | 0,07 | 2,04 | 2,3 |
|  | 15 cm | 3 | 2,2 | 0,1 | 0,06 | 2,09 | 2,31 |
|  | Total | 12 | 2,18 | 0,083 | 0,02 | 2,14 | 2,23 |
| Vertical | No extension | 3 | 2,4 | 0,2 | 0,12 | 2,17 | 2,63 |
|  | 5 cm | 3 | 2,33 | 0,058 | 0,03 | 2,27 | 2,4 |
|  | 10 cm | 3 | 2,17 | 0,058 | 0,03 | 2,1 | 2,23 |
|  | 15 cm | 3 | 2,2 | 0,173 | 0,1 | 2 | 2,4 |
|  | Total | 12 | 2,28 | 0,154 | 0,04 | 2,19 | 2,36 |
| Mean | No extension | 3 | 2,3 | 0,179 | 0,07 | 2,16 | 2,44 |
|  | 5 cm | 3 | 2,25 | 0,105 | 0,04 | 2,17 | 2,33 |
|  | 10 cm | 3 | 2,17 | 0,082 | 0,03 | 2,1 | 2,23 |
|  | 15 cm | 3 | 2,2 | 0,126 | 0,05 | 2,1 | 2,3 |
|  | Total | 12 | 2,23 | 0,13 | 0,03 | 2,18 | 2,28 |

**Table 84** Entry Hole Variance Analysis at 700cm Distance based on Extension Lengths.

| **ANOVA** | | | | | |
| --- | --- | --- | --- | --- | --- |
| Measurement on Target | |  |  |  |  |
|  | Sums of Squares  **(SS)** | Degrees of Freedom **(df)** | Means Squares **(MS)** | F Value | Significance |
| Between Groups | ,061 | 3 | ,020 | 1,244 | ,320 |
| In Groups | ,328 | 20 | ,016 |  |  |
| Total | ,390 | 23 |  |  |  |

**Table 85** Entry Hole Diameters Multiple Comparisons at 700cm Distance based on Extension Lengths.

| **Multiple Comparison** | | | | | | |
| --- | --- | --- | --- | --- | --- | --- |
| Measurement on Target  Tukey HSD | | |  |  |  |  |
| (I) Extension  Length | (J) Extension  Length | Mean Difference  (I-J) | Standard  Error | Significance | 95% Confidence Interval | |
|  |  |  |  |  | Lower Limit | Upper Limit |
| No extension | 5 cm | ,05000 | ,07397 | ,905 | -,1570 | ,2570 |
|  | 10 cm | ,13333 | ,07397 | ,301 | -,0737 | ,3404 |
|  | 15 cm | ,10000 | ,07397 | ,542 | -,1070 | ,3070 |
| 5 cm | No extension | -,05000 | ,07397 | ,905 | -,2570 | ,1570 |
|  | 10 cm | ,08333 | ,07397 | ,678 | -,1237 | ,2904 |
|  | 15 cm | ,05000 | ,07397 | ,905 | -,1570 | ,2570 |
| 10 cm | No extension | -,13333 | ,07397 | ,301 | -,3404 | ,0737 |
|  | 5 cm | -,08333 | ,07397 | ,678 | -,2904 | ,1237 |
|  | 15 cm | -,03333 | ,07397 | ,969 | -,2404 | ,1737 |
| 15 cm | No extension | -,10000 | ,07397 | ,542 | -,3070 | ,1070 |
|  | 5 cm | -,05000 | ,07397 | ,905 | -,2570 | ,1570 |
|  | 10 cm | ,03333 | ,07397 | ,969 | -,1737 | ,2404 |

**Table 86** Pellet Distribution on at 700cm distances based on Extension Lengths.

| Measurement  Type | Extension  Lenght | Number  Of  Shots | Arithmetic Mean  (cm) | Standard Deviation | Standard Error | Confidence Interval (%95)  Lower Limit | Confidence Interval (%95)  Upper Limit |
| --- | --- | --- | --- | --- | --- | --- | --- |
| Horizantal | No extension | 3 | 37,5 | 1,127 | 0,651 | 36,22 | 38,78 |
|  | 5 cm | 3 | 33,3 | 0,458 | 0,265 | 32,78 | 33,82 |
|  | 10 cm | 3 | 31,27 | 0,503 | 0,291 | 30,7 | 31,84 |
|  | 15 cm | 3 | 29,03 | 0,153 | 0,088 | 28,86 | 29,21 |
|  | Total | 12 | 32,78 | 3,305 | 0,954 | 30,91 | 34,64 |
| Vertical | No extension | 3 | 37,67 | 1,358 | 0,784 | 36,13 | 39,2 |
|  | 5 cm | 3 | 33,67 | 0,513 | 0,296 | 33,09 | 34,25 |
|  | 10 cm | 3 | 31,57 | 0,757 | 0,437 | 30,71 | 32,42 |
|  | 15 cm | 3 | 29,1 | 1,127 | 0,651 | 27,82 | 30,38 |
|  | Total | 12 | 33 | 3,389 | 0,978 | 31,08 | 34,92 |
| Mean | No extension | 3 | 37,58 | 1,12 | 0,457 | 36,69 | 38,48 |
|  | 5 cm | 3 | 33,48 | 0,479 | 0,196 | 33,1 | 33,87 |
|  | 10 cm | 3 | 31,42 | 0,598 | 0,244 | 30,94 | 31,9 |
|  | 15 cm | 3 | 29,07 | 0,72 | 0,294 | 28,49 | 29,64 |
|  | Total | 12 | 32,89 | 3,276 | 0,669 | 31,58 | 34,2 |

**Table 87** Pellet Distribution Diameter Variance Analysis at 700cm Distances Based on Extension Lengths.

| **ANOVA** | | | | | |
| --- | --- | --- | --- | --- | --- |
| Measurement on Target | |  |  |  |  |
|  | Sums of Squares  **(SS)** | Degrees of Freedom **(df)** | Means Squares **(MS)** | F Value | Significance |
| Between Groups | 235,008 | 3 | 78,336 | 132,792 | ,000 |
| In Groups | 11,798 | 20 | ,590 |  |  |
| Total | 246,806 | 23 |  |  |  |

**Table 88** Pellet Distribution Diameter Multiple Comparison Analysis at 700cm Distances Based on Extension Lengths..

| **Multiple Comparison** | | | | | | |
| --- | --- | --- | --- | --- | --- | --- |
| Measurement on Target  Tukey HSD | | |  |  |  |  |
| (I) Extension  Lenght | (J) Extension  Lenght | Mean Difference  (I-J) | Standard  Error | Significance | 95% Confidence Interval | |
|  |  |  |  |  | Lower Limit | Upper Limit |
| No extension | 5 cm | 4,10000* | ,44344 | ,000 | 2,8588 | 5,3412 |
|  | 10 cm | 6,16667* | ,44344 | ,000 | 4,9255 | 7,4078 |
|  | 15 cm | 8,51667* | ,44344 | ,000 | 7,2755 | 9,7578 |
| 5 cm | No extension | -4,10000* | ,44344 | ,000 | -5,3412 | -2,8588 |
|  | 10 cm | 2,06667* | ,44344 | ,001 | ,8255 | 3,3078 |
|  | 15 cm | 4,41667* | ,44344 | ,000 | 3,1755 | 5,6578 |
| 10 cm | No extension | -6,16667* | ,44344 | ,000 | -7,4078 | -4,9255 |
|  | 5 cm | -2,06667* | ,44344 | ,001 | -3,3078 | -,8255 |
|  | 15 cm | 2,35000* | ,44344 | ,000 | 1,1088 | 3,5912 |
| 15 cm | No extension | -8,51667* | ,44344 | ,000 | -9,7578 | -7,2755 |
|  | 5 cm | -4,41667* | ,44344 | ,000 | -5,6578 | -3,1755 |
|  | 10 cm | -2,35000* | ,44344 | ,000 | -3,5912 | -1,1088 |

**Çizelge 89** The farthest satellite entry holes at 700cm distance based on Extension Lenghts.

| Extension  Lenght | Number  Of  Shots | Arithmetic Mean  (cm) | Standard Deviation | Standard Error | Confidence Interval (%95)  Lower Limit | Confidence Interval (%95)  Upper Limit |
| --- | --- | --- | --- | --- | --- | --- |
| No Extension | 3 | 24,5 | 3,381 | 1,952 | 20,67 | 28,33 |
| 5 cm | 3 | 22,67 | 1,041 | 0,601 | 21,49 | 23,84 |
| 10 cm | 3 | 24,27 | 4,594 | 2,652 | 19,07 | 29,46 |
| 15 cm | 3 | 26,87 | 2,25 | 1,299 | 24,32 | 29,41 |
| Total | 12 | 24,58 | 3,08 | 0,889 | 22,83 | 26,32 |

**Table 90** The farthest satellite entry holes Variance Analysis at 700cm Distances Based on Extension Lengths.

| **ANOVA** | | | | | |
| --- | --- | --- | --- | --- | --- |
| Measurement on Target | |  |  |  |  |
|  | Sums of Squares  **(SS)** | Degrees of Freedom **(df)** | Means Squares **(MS)** | F Value | Significance |
| Between Groups | 26,982 | 3 | 8,994 | ,930 | ,469 |
| In Groups | 77,360 | 8 | 9,670 |  |  |
| Total | 104,342 | 11 |  |  |  |

**Table 91** The farthest satellite entry holes Multiple Comparison Analysis at 700cm Distances Based on Extension Lengths.

| **Multiple Comparison** | | | | | | |
| --- | --- | --- | --- | --- | --- | --- |
| Measurement on Target  Tukey HSD | | |  |  |  |  |
| (I) Extension  Lenght | (J) Extension  Lenght | Mean Difference  (I-J) | Standard  Error | Significance | 95% Confidence Interval | |
|  |  |  |  |  | Lower Limit | Upper Limit |
| No extension | 5 cm | 1,83333 | 2,53903 | ,886 | -6,2975 | 9,9642 |
|  | 10 cm | ,23333 | 2,53903 | 1,000 | -7,8975 | 8,3642 |
|  | 15 cm | -2,36667 | 2,53903 | ,789 | -10,4975 | 5,7642 |
| 5 cm | No extension | -1,83333 | 2,53903 | ,886 | -9,9642 | 6,2975 |
|  | 10 cm | -1,60000 | 2,53903 | ,919 | -9,7309 | 6,5309 |
|  | 15 cm | -4,20000 | 2,53903 | ,404 | -12,3309 | 3,9309 |
| 10 cm | No extension | -,23333 | 2,53903 | 1,000 | -8,3642 | 7,8975 |
|  | 5 cm | 1,60000 | 2,53903 | ,919 | -6,5309 | 9,7309 |
|  | 15 cm | -2,60000 | 2,53903 | ,741 | -10,7309 | 5,5309 |
| 15 cm | No extension | 2,36667 | 2,53903 | ,789 | -5,7642 | 10,4975 |
|  | 5 cm | 4,20000 | 2,53903 | ,404 | -3,9309 | 12,3309 |
|  | 10 cm | 2,60000 | 2,53903 | ,741 | -5,5309 | 10,7309 |

**Table 92** Entry hole diameters based on extension length at 800cm range

| Measurement  Type | Extension  Lenght | Number  Of  Shots | Arithmetic Mean  (cm) | Standard Deviation | Standard Error | Confidence Interval (%95)  Lower Limit | Confidence Interval (%95)  Upper Limit |
| --- | --- | --- | --- | --- | --- | --- | --- |
| Horizantal | No extension | 3 | 2,23 | 0,153 | 0,09 | 2,06 | 2,41 |
|  | 5 cm | 3 | 2,13 | 0,153 | 0,09 | 1,96 | 2,31 |
|  | 10 cm | 3 | 2,2 | 0,1 | 0,06 | 2,09 | 2,31 |
|  | 15 cm | 3 | 2,3 | 0,1 | 0,06 | 2,19 | 2,41 |
|  | Total | 12 | 2,22 | 0,127 | 0,04 | 2,14 | 2,29 |
| Vertical | No extension | 3 | 2,23 | 0,153 | 0,09 | 2,06 | 2,41 |
|  | 5 cm | 3 | 2,33 | 0,058 | 0,03 | 2,27 | 2,4 |
|  | 10 cm | 3 | 2,2 | 0,2 | 0,12 | 1,97 | 2,43 |
|  | 15 cm | 3 | 2,17 | 0,058 | 0,03 | 2,1 | 2,23 |
|  | Total | 12 | 2,23 | 0,13 | 0,04 | 2,16 | 2,31 |
| Mean | No extension | 3 | 2,23 | 0,137 | 0,06 | 2,12 | 2,34 |
|  | 5 cm | 3 | 2,23 | 0,151 | 0,06 | 2,11 | 2,35 |
|  | 10 cm | 3 | 2,2 | 0,141 | 0,06 | 2,09 | 2,31 |
|  | 15 cm | 3 | 2,23 | 0,103 | 0,04 | 2,15 | 2,32 |
|  | Total | 12 | 2,23 | 0,126 | 0,03 | 2,17 | 2,28 |

**Table 93** Entry Hole Variance Analysis at 800cm Distance based on Extension Lengths.

| **ANOVA** | | | | | |
| --- | --- | --- | --- | --- | --- |
| Measurement on Target | |  |  |  |  |
|  | Sums of Squares  **(SS)** | Degrees of Freedom **(df)** | Means Squares **(MS)** | F Value | Significance |
| Between Groups | ,005 | 3 | ,002 | ,093 | ,963 |
| In Groups | ,360 | 20 | ,018 |  |  |
| Total | ,365 | 23 |  |  |  |

**Table 94** Entry Hole Diameters Multiple Comparisons at 800cm Distance based on Extension Lengths..

| **Multiple Comparison** | | | | | | |
| --- | --- | --- | --- | --- | --- | --- |
| Measurement on Target  Tukey HSD | | |  |  |  |  |
| (I) Extension  Lenght | (J) Extension  Lenght | Mean Difference  (I-J) | Standard  Error | Significance | 95% Confidence Interval | |
|  |  |  |  |  | Lower Limit | Upper Limit |
| No extension | 5 cm | ,00000 | ,07746 | 1,000 | -,2168 | ,2168 |
|  | 10 cm | ,03333 | ,07746 | ,973 | -,1835 | ,2501 |
|  | 15 cm | ,00000 | ,07746 | 1,000 | -,2168 | ,2168 |
| 5 cm | No extension | ,00000 | ,07746 | 1,000 | -,2168 | ,2168 |
|  | 10 cm | ,03333 | ,07746 | ,973 | -,1835 | ,2501 |
|  | 15 cm | ,00000 | ,07746 | 1,000 | -,2168 | ,2168 |
| 10 cm | No extension | -,03333 | ,07746 | ,973 | -,2501 | ,1835 |
|  | 5 cm | -,03333 | ,07746 | ,973 | -,2501 | ,1835 |
|  | 15 cm | -,03333 | ,07746 | ,973 | -,2501 | ,1835 |
| 15 cm | No extension | ,00000 | ,07746 | 1,000 | -,2168 | ,2168 |
|  | 5 cm | ,00000 | ,07746 | 1,000 | -,2168 | ,2168 |
|  | 10 cm | ,03333 | ,07746 | ,973 | -,1835 | ,2501 |

**Çizelge 95** Pellet Distribution on at 800cm distances based on Extension Lengths.

| Measurement  Type | Extension  Lenght | Number  Of  Shots | Arithmetic Mean  (cm) | Standard Deviation | Standard Error | Confidence Interval (%95)  Lower Limit | Confidence Interval (%95)  Upper Limit |
| --- | --- | --- | --- | --- | --- | --- | --- |
| Horizantal | No extension | 3 | 44,87 | 0,702 | 0,406 | 44,07 | 45,66 |
|  | 5 cm | 3 | 42,93 | 0,153 | 0,088 | 42,76 | 43,11 |
|  | 10 cm | 3 | 41,33 | 0,586 | 0,338 | 40,67 | 42 |
|  | 15 cm | 3 | 36,47 | 0,751 | 0,433 | 35,62 | 37,32 |
|  | Total | 12 | 41,4 | 3,289 | 0,949 | 39,54 | 43,26 |
| Vertical | No extension | 3 | 46,1 | 0,2 | 0,115 | 45,87 | 46,33 |
|  | 5 cm | 3 | 42,33 | 0,153 | 0,088 | 42,16 | 42,51 |
|  | 10 cm | 3 | 40,93 | 0,551 | 0,318 | 40,31 | 41,56 |
|  | 15 cm | 3 | 36,33 | 0,503 | 0,291 | 35,76 | 36,9 |
|  | Total | 12 | 41,43 | 3,665 | 1,058 | 39,35 | 43,5 |
| Mean | No extension | 3 | 45,48 | 0,818 | 0,334 | 44,83 | 46,14 |
|  | 5 cm | 3 | 42,63 | 0,356 | 0,145 | 42,35 | 42,92 |
|  | 10 cm | 3 | 41,13 | 0,554 | 0,226 | 40,69 | 41,58 |
|  | 15 cm | 3 | 36,4 | 0,576 | 0,235 | 35,94 | 36,86 |
|  | Total | 12 | 41,41 | 3,406 | 0,695 | 40,05 | 42,78 |

**Çizelge 96** Pellet Distribution Diameter Variance Analysis at 800cm Distances Based on Extension Lengths.

| **ANOVA** | | | | | |
| --- | --- | --- | --- | --- | --- |
| Measurement on Target | |  |  |  |  |
|  | Sums of Squares  **(SS)** | Degrees of Freedom **(df)** | Means Squares **(MS)** | F Value | Significance |
| Between Groups | 259,591 | 3 | 86,530 | 241,200 | ,000 |
| In Groups | 7,175 | 20 | ,359 |  |  |
| Total | 266,766 | 23 |  |  |  |

**Table 97** Pellet Distribution Diameter Multiple Comparison Analysis at 800cm Distances Based on Extension Lengths..

| **Multiple Comparison** | | | | | | |
| --- | --- | --- | --- | --- | --- | --- |
| Measurement on Target  Tukey HSD | | |  |  |  |  |
| (I) Extension  Lenght | (J) Extension  Lenght | Mean Difference  (I-J) | Standard  Error | Significance | 95% Confidence Interval | |
|  |  |  |  |  | Lower Limit | Upper Limit |
| No extension | 5 cm | 2,85000* | ,34581 | ,000 | 1,8821 | 3,8179 |
|  | 10 cm | 4,35000* | ,34581 | ,000 | 3,3821 | 5,3179 |
|  | 15 cm | 9,08333* | ,34581 | ,000 | 8,1154 | 10,0512 |
| 5 cm | No extension | -2,85000* | ,34581 | ,000 | -3,8179 | -1,8821 |
|  | 10 cm | 1,50000* | ,34581 | ,002 | ,5321 | 2,4679 |
|  | 15 cm | 6,23333* | ,34581 | ,000 | 5,2654 | 7,2012 |
| 10 cm | No extension | -4,35000* | ,34581 | ,000 | -5,3179 | -3,3821 |
|  | 5 cm | -1,50000* | ,34581 | ,002 | -2,4679 | -,5321 |
|  | 15 cm | 4,73333* | ,34581 | ,000 | 3,7654 | 5,7012 |
| 15 cm | No extension | -9,08333* | ,34581 | ,000 | -10,0512 | -8,1154 |
|  | 5 cm | -6,23333* | ,34581 | ,000 | -7,2012 | -5,2654 |
|  | 10 cm | -4,73333* | ,34581 | ,000 | -5,7012 | -3,7654 |

**Table 98** The farthest satellite entry holes at 800cm distance based on Extension Lenghts.

| Extension  Lenght | Number  Of  Shots | Arithmetic Mean  (cm) | Standard Deviation | Standard Error | Confidence Interval (%95)  Lower Limit | Confidence Interval (%95)  Upper Limit |
| --- | --- | --- | --- | --- | --- | --- |
| No extension | 3 | 30,93 | 2,173 | 1,255 | 28,47 | 33,39 |
| 5 cm | 3 | 30,33 | 2,483 | 1,433 | 27,52 | 33,14 |
| 10 cm | 3 | 30,73 | 1,069 | 0,617 | 29,52 | 31,94 |
| 15 cm | 3 | 29,33 | 2,954 | 1,705 | 25,99 | 32,68 |
| Total | 12 | 30,33 | 2,046 | 0,591 | 29,18 | 31,49 |

**Table 99** The farthest satellite entry holes Variance Analysis at 800cm Distances Based on Extension Lengths.

| **ANOVA** | | | | | |
| --- | --- | --- | --- | --- | --- |
| Measurement on Target | |  |  |  |  |
|  | Sums of Squares  **(SS)** | Degrees of Freedom **(df)** | Means Squares **(MS)** | F Value | Significance |
| Between Groups | 4,560 | 3 | 1,520 | ,293 | ,830 |
| In Groups | 41,507 | 8 | 5,188 |  |  |
| Total | 46,067 | 11 |  |  |  |

**Table 100** The farthest satellite entry holes Multiple Comparison Analysis at 800cm Distances Based on Extension Lengths.

| **Multiple Comparison** | | | | | | |
| --- | --- | --- | --- | --- | --- | --- |
| Measurement on Target  Tukey HSD | | |  |  |  |  |
| (I) Extension  Lenght | (J) Extension  Lenght | Mean Difference  (I-J) | Standard  Error | Significance | 95% Confidence Interval | |
|  |  |  |  |  | Lower Limit | Upper Limit |
| No extension | 5 cm | ,60000 | 1,85981 | ,988 | -5,3558 | 6,5558 |
|  | 10 cm | ,20000 | 1,85981 | 1,000 | -5,7558 | 6,1558 |
|  | 15 cm | 1,60000 | 1,85981 | ,825 | -4,3558 | 7,5558 |
| 5 cm | No extension | -,60000 | 1,85981 | ,988 | -6,5558 | 5,3558 |
|  | 10 cm | -,40000 | 1,85981 | ,996 | -6,3558 | 5,5558 |
|  | 15 cm | 1,00000 | 1,85981 | ,947 | -4,9558 | 6,9558 |
| 10 cm | No extension | -,20000 | 1,85981 | 1,000 | -6,1558 | 5,7558 |
|  | 5 cm | ,40000 | 1,85981 | ,996 | -5,5558 | 6,3558 |
|  | 15 cm | 1,40000 | 1,85981 | ,873 | -4,5558 | 7,3558 |
| 15 cm | No extension | -1,60000 | 1,85981 | ,825 | -7,5558 | 4,3558 |
|  | 5 cm | -1,00000 | 1,85981 | ,947 | -6,9558 | 4,9558 |
|  | 10 cm | -1,40000 | 1,85981 | ,873 | -7,3558 | 4,5558 |

**Table 101** Entry hole diameters based on extension length at 1000cm range

| Measurement  Type | Extension  Lenght | Number  Of  Shots | Arithmetic Mean  (cm) | Standard Deviation | Standard Error | Confidence Interval (%95)  Lower Limit | Confidence Interval (%95)  Upper Limit |
| --- | --- | --- | --- | --- | --- | --- | --- |
| Horizantal | No extension | 3 | 2,17 | 0,058 | 0,03 | 2,1 | 2,23 |
|  | 5 cm | 3 | 2,17 | 0,115 | 0,07 | 2,04 | 2,3 |
|  | 10 cm | 3 | 2,23 | 0,115 | 0,07 | 2,1 | 2,36 |
|  | 15 cm | 3 | 2,17 | 0,058 | 0,03 | 2,1 | 2,23 |
|  | Total | 12 | 2,18 | 0,083 | 0,02 | 2,14 | 2,23 |
| Vertical | No extension | 3 | 2,17 | 0,115 | 0,07 | 2,04 | 2,3 |
|  | 5 cm | 3 | 2,2 | 0,1 | 0,06 | 2,09 | 2,31 |
|  | 10 cm | 3 | 2,23 | 0,058 | 0,03 | 2,17 | 2,3 |
|  | 15 cm | 3 | 2,23 | 0,115 | 0,07 | 2,1 | 2,36 |
|  | Total | 12 | 2,21 | 0,09 | 0,03 | 2,16 | 2,26 |
| Mean | No extension | 3 | 2,17 | 0,082 | 0,03 | 2,1 | 2,23 |
|  | 5 cm | 3 | 2,18 | 0,098 | 0,04 | 2,1 | 2,26 |
|  | 10 cm | 3 | 2,23 | 0,082 | 0,03 | 2,17 | 2,3 |
|  | 15 cm | 3 | 2,2 | 0,089 | 0,04 | 2,13 | 2,27 |
|  | Total | 12 | 2,2 | 0,086 | 0,02 | 2,16 | 2,23 |

**Table 102** Entry Hole Variance Analysis at 1000cm Distance based on Extension Lengths.

| **ANOVA** | | | | | |
| --- | --- | --- | --- | --- | --- |
| Measurement on Target | |  |  |  |  |
|  | Sums of Squares  **(SS)** | Degrees of Freedom **(df)** | Means Squares **(MS)** | F Value | Significance |
| Between Groups | ,015 | 3 | ,005 | ,627 | ,606 |
| In Groups | ,155 | 20 | ,008 |  |  |
| Total | ,170 | 23 |  |  |  |

**Table 103** Entry Hole Diameters Multiple Comparisons at 1000cm Distance based on Extension Lengths.

| **Multiple Comparison** | | | | | | |
| --- | --- | --- | --- | --- | --- | --- |
| Measurement on Target  Tukey HSD | | |  |  |  |  |
| (I) Extension  Lenght | (J) Extension  Lenght | Mean Difference  (I-J) | Standard  Error | Significance | 95% Confidence Interval | |
|  |  |  |  |  | Lower Limit | Upper Limit |
| No extension | 5 cm | -,01667 | ,05083 | ,987 | -,1589 | ,1256 |
|  | 10 cm | -,06667 | ,05083 | ,566 | -,2089 | ,0756 |
|  | 15 cm | -,03333 | ,05083 | ,912 | -,1756 | ,1089 |
| 5 cm | No extension | ,01667 | ,05083 | ,987 | -,1256 | ,1589 |
|  | 10 cm | -,05000 | ,05083 | ,760 | -,1923 | ,0923 |
|  | 15 cm | -,01667 | ,05083 | ,987 | -,1589 | ,1256 |
| 10 cm | No extension | ,06667 | ,05083 | ,566 | -,0756 | ,2089 |
|  | 5 cm | ,05000 | ,05083 | ,760 | -,0923 | ,1923 |
|  | 15 cm | ,03333 | ,05083 | ,912 | -,1089 | ,1756 |
| 15 cm | No extension | ,03333 | ,05083 | ,912 | -,1089 | ,1756 |
|  | 5 cm | ,01667 | ,05083 | ,987 | -,1256 | ,1589 |
|  | 10 cm | -,03333 | ,05083 | ,912 | -,1756 | ,1089 |

**Table 104** Pellet Distribution on at 1000cm distances based on Extension Lengths.

| Measurement  Type | Extension  Lenght | Number  Of  Shots | Arithmetic Mean  (cm) | Standard Deviation | Standard Error | Confidence Interval (%95)  Lower Limit | Confidence Interval (%95)  Upper Limit |
| --- | --- | --- | --- | --- | --- | --- | --- |
| Horizantal | No extension | 3 | 53,17 | 0,777 | 0,448 | 52,29 | 54,05 |
|  | 5 cm | 3 | 48,77 | 1,877 | 1,084 | 46,64 | 50,89 |
|  | 10 cm | 3 | 46,6 | 0,265 | 0,153 | 46,3 | 46,9 |
|  | 15 cm | 3 | 42,67 | 1,305 | 0,754 | 41,19 | 44,14 |
|  | Total | 12 | 47,8 | 4,094 | 1,182 | 45,48 | 50,12 |
| Vertical | No extension | 3 | 51,93 | 2,593 | 1,497 | 49 | 54,87 |
|  | 5 cm | 3 | 47,23 | 3,287 | 1,898 | 43,51 | 50,95 |
|  | 10 cm | 3 | 45,93 | 0,153 | 0,088 | 45,76 | 46,11 |
|  | 15 cm | 3 | 42,13 | 1,35 | 0,78 | 40,61 | 43,66 |
|  | Total | 12 | 46,81 | 4,112 | 1,187 | 44,48 | 49,13 |
| Mean | No extension | 3 | 52,55 | 1,84 | 0,751 | 51,08 | 54,02 |
|  | 5 cm | 3 | 48 | 2,537 | 1,036 | 45,97 | 50,03 |
|  | 10 cm | 3 | 46,27 | 0,413 | 0,169 | 45,94 | 46,6 |
|  | 15 cm | 3 | 42,4 | 1,223 | 0,499 | 41,42 | 43,38 |
|  | Total | 12 | 47,3 | 4,044 | 0,826 | 45,69 | 48,92 |

**Table 105** Pellet Distribution Diameter Variance Analysis at 1000cm Distances Based on Extension Lengths.

| **ANOVA** | | | | | |
| --- | --- | --- | --- | --- | --- |
| Measurement on Target | |  |  |  |  |
|  | Sums of Squares  **(SS)** | Degrees of Freedom **(df)** | Means Squares **(MS)** | F Value | Significance |
| Between Groups | 318,781 | 3 | 106,260 | 36,993 | ,000 |
| In Groups | 57,448 | 20 | 2,872 |  |  |
| Total | 376,230 | 23 |  |  |  |

**Table 106** Pellet Distribution Diameter Multiple Comparison Analysis at 1000cm Distances Based on Extension Lengths..

| **Çoklu Karşılaştırma** | | | | | | |
| --- | --- | --- | --- | --- | --- | --- |
| Measurement on Target  Tukey HSD | | |  |  |  |  |
| (I) Extension  Lenght | (J) Extension  Lenght | Mean Difference  (I-J) | Standard  Error | Significance | 95% Confidence Interval | |
|  |  |  |  |  | Lower Limit | Upper Limit |
| No extension | 5 cm | 4,55000* | ,97851 | ,001 | 1,8112 | 7,2888 |
|  | 10 cm | 6,28333* | ,97851 | ,000 | 3,5446 | 9,0221 |
|  | 15 cm | 10,15000* | ,97851 | ,000 | 7,4112 | 12,8888 |
| 5 cm | No extension | -4,55000* | ,97851 | ,001 | -7,2888 | -1,8112 |
|  | 10 cm | 1,73333 | ,97851 | ,316 | -1,0054 | 4,4721 |
|  | 15 cm | 5,60000* | ,97851 | ,000 | 2,8612 | 8,3388 |
| 10 cm | No extension | -6,28333* | ,97851 | ,000 | -9,0221 | -3,5446 |
|  | 5 cm | -1,73333 | ,97851 | ,316 | -4,4721 | 1,0054 |
|  | 15 cm | 3,86667* | ,97851 | ,004 | 1,1279 | 6,6054 |
| 15 cm | No extension | -10,15000* | ,97851 | ,000 | -12,8888 | -7,4112 |
|  | 5 cm | -5,60000* | ,97851 | ,000 | -8,3388 | -2,8612 |
|  | 10 cm | -3,86667* | ,97851 | ,004 | -6,6054 | -1,1279 |

**Table 107** The farthest satellite entry holes at 1000cm distance based on Extension Lenghts.

| Extension  Lenght | Number  Of  Shots | Arithmetic Mean  (cm) | Standard Deviation | Standard Error | Confidence Interval (%95)  Lower Limit | Confidence Interval (%95)  Upper Limit |
| --- | --- | --- | --- | --- | --- | --- |
| No extension | 3 | 33 | 4,453 | 2,571 | 27,96 | 38,04 |
| 5 cm | 3 | 29,13 | 1,401 | 0,809 | 27,55 | 30,72 |
| 10 cm | 3 | 31,13 | 1,274 | 0,736 | 29,69 | 32,58 |
| 15 cm | 3 | 30,07 | 1,756 | 1,014 | 28,08 | 32,05 |
| Total | 12 | 30,83 | 2,659 | 0,768 | 29,33 | 32,34 |

**Table 108** The farthest satellite entry holes Variance Analysis at 1000cm Distances Based on Extension Lengths.

| **ANOVA** | | | | | |
| --- | --- | --- | --- | --- | --- |
| Measurement on Target | |  |  |  |  |
|  | Sums of Squares  **(SS)** | Degrees of Freedom **(df)** | Means Squares **(MS)** | F Value | Significance |
| Between Groups | 24,787 | 3 | 8,262 | 1,247 | ,355 |
| In Groups | 53,000 | 8 | 6,625 |  |  |
| Total | 77,787 | 11 |  |  |  |

**Table 109** The farthest satellite entry holes Multiple Comparison Analysis at 1000cm Distances Based on Extension Lengths

| **Multiple Comparison** | | | | | | |
| --- | --- | --- | --- | --- | --- | --- |
| Measurement on Target  Tukey HSD | | |  |  |  |  |
| (I) Extension  Lenght | (J) Extension  Lenght | Mean Difference  (I-J) | Standard  Error | Significance | 95% Confidence Interval | |
|  |  |  |  |  | Lower Limit | Upper Limit |
| No extension | 5 cm | 3,86667 | 2,10159 | ,323 | -2,8634 | 10,5967 |
|  | 10 cm | 1,86667 | 2,10159 | ,811 | -4,8634 | 8,5967 |
|  | 15 cm | 2,93333 | 2,10159 | ,535 | -3,7967 | 9,6634 |
| 5 cm | No extension | -3,86667 | 2,10159 | ,323 | -10,5967 | 2,8634 |
|  | 10 cm | -2,00000 | 2,10159 | ,779 | -8,7300 | 4,7300 |
|  | 15 cm | -,93333 | 2,10159 | ,969 | -7,6634 | 5,7967 |
| 10 cm | No extension | -1,86667 | 2,10159 | ,811 | -8,5967 | 4,8634 |
|  | 5 cm | 2,00000 | 2,10159 | ,779 | -4,7300 | 8,7300 |
|  | 15 cm | 1,06667 | 2,10159 | ,955 | -5,6634 | 7,7967 |
| 15 cm | No extension | -2,93333 | 2,10159 | ,535 | -9,6634 | 3,7967 |
|  | 5 cm | ,93333 | 2,10159 | ,969 | -5,7967 | 7,6634 |
|  | 10 cm | -1,06667 | 2,10159 | ,955 | -7,7967 | 5,6634 |
